# Gender-specific differences in hypothalamus–pituitary–adrenal axis activity during childhood: a systematic review and meta-analysis

**DOI:** 10.1186/s13293-016-0123-5

**Published:** 2017-01-19

**Authors:** Bibian van der Voorn, Jonneke J. Hollanders, Johannes C. F. Ket, Joost Rotteveel, Martijn J. J. Finken

**Affiliations:** 1 Department of Pediatric Endocrinology, VU University Medical Center, Postbus 7057, 1007 MB Amsterdam, The Netherlands; 2 Medical Library, Vrije Universiteit, De Boelelaan 1103, 1081 HV Amsterdam, The Netherlands

**Keywords:** Glucocorticoid, Stress hormone, Infant, Pediatric, Sex characteristics

## Abstract

**Background:**

Gender-specific differences in hypothalamus–pituitary–adrenal (HPA) axis activity have been postulated to emerge during puberty. We conducted a systematic review and meta-analysis to test the hypothesis that gender-specific differences in HPA axis activity are already present in childhood.

**Methods:**

From inception to January 2016, PubMed and EMBASE.com were searched for studies that assessed non-stimulated cortisol in serum or saliva or cortisol in 24-h urine in healthy males and females aged ≤18 years. Studies that conform with the Preferred Reporting Items for Systematic Reviews and Meta-Analysis (PRISMA) statement were reported. Standardized mean differences (95% CIs) were calculated and analyzed using fixed-effect meta-analysis stratified for age: <8 years (prepubertal) and 8–18 years (peri-/postpubertal). For comparison, we ran the same analyses using random-effects models.

**Results:**

Two independent assessors selected 413 out of 6158 records (7%) for full-text screening, of which 79 articles were included. Of these, 58 (with data on 16,551 subjects) were included in the meta-analysis. Gender differences in cortisol metabolism differed per age group. Boys aged <8 years had 0.18 (0.06; 0.30) nmol/L higher serum and 0.21 (0.05; 0.37) nmol/L higher salivary cortisol levels, while between 8 and 18 years, boys had 0.34 (0.28; 0.40) nmol/L lower serum and 0.42 (0.38; 0.47) nmol/L lower salivary cortisol levels. In 24-h urine, cortisol was consistently higher in boys, being 0.34 (0.05; 0.64) and 0.32 (0.17; 0.47) μg/24 h higher in the <8- and 8–18-year groups, respectively. However, gender-differences in serum cortisol <8 years and between 8 and 18 years were absent when using random-effects models.

**Conclusions:**

Gender differences in cortisol metabolism are already present in childhood, with higher salivary cortisol in boys aged <8 years compared to girls. This pattern was reversed after the age of 8 years. In contrast, the gender-specific difference in cortisol production as assessed through 24-h urine did not change with age. Although differences were small, and analyses of gender differences in serum cortisol were inconclusive, they might contribute to gender-specific origins of health and disease.

**Electronic supplementary material:**

The online version of this article (doi:10.1186/s13293-016-0123-5) contains supplementary material, which is available to authorized users.

## Background

The hypothalamus–pituitary–adrenal (HPA) and hypothalamus–pituitary–gonadal (HPG) axes are closely connected. Animal studies demonstrated that corticotropin releasing hormone (CRH) inhibits the HPG axis at all levels, while testosterone inhibits the HPA axis at the hypothalamic level. Additionally, estrogens stimulate the HPA axis at both the hypothalamic and adrenal levels. Moreover, CRH levels were dependent on the phase of the menstrual cycle, with the highest concentrations occurring during the follicular phase [1, 2].

Human studies suggested that estrogens decrease the hepatic A-ring reduction of cortisol, albeit not in the short term [3], and increase the production of corticosteroid-binding globulin (CBG), thereby affecting the bioavailability of cortisol [1, 4, 5]. The latter is being enhanced by the use of oral contraceptives. Furthermore, HPA-axis responses to acute psychological stress were different depending on the phase of the menstrual cycle [2, 4].

Due to an increase in sex steroid concentrations, gender differences in HPA axis activity have been postulated to emerge during puberty [6, 7]. However, more recent evidence suggests that gender differences in HPA axis activity are already present early in life [1, 8, 9]. Putative mediators of these prepubertal gender differences are the postnatal reproductive hormone surge, also known as mini-puberty [10], and sex-specific effects of styles in parental care, such as psychosocial stress reactivity to maternal over-controlling behavior [11]. However, physiological gender differences in cortisol concentrations during childhood have not been studied yet.

Therefore, the question was raised whether gender differences in unstimulated HPA axis activity emerge during puberty or whether they are already present earlier in life. Accordingly, we conducted a systematic review and meta-analysis with the hypothesis that gender-specific differences in unstimulated HPA axis activity are present in early life and are subsequently influenced by puberty.

## Methods

### Search strategy

From inception up to 14 January 2016, PubMed and EMBASE.com were searched (by BvdV and JCFK) for studies that reported non-stimulated cortisol in serum or saliva or cortisol in 24-h urine for healthy boys and girls aged ≤18 years separately. Additional file 1 presents the full search strategy, which was based on the following index terms or free-text words: “cortisol” or “glucocorticoid”, and “sex difference” or “sexual characteristics”, and “child” or “adolescent”. Studies in children with (psycho) pathology, on synthetic glucocorticoids, or with risk for abnormal HPA axis activity (e.g., a history of maltreatment) were excluded. An English language restriction was applied for abstracts of published articles. No restrictions for year of publication or study design, apart from reviews and case reports, were applied. The review protocol was based on the Preferred Reporting Items for Systematic Reviews and Meta-Analysis (PRISMA) statement.

### Data collection

Two independent assessors (BvdV and JJH) screened 6158 titles and abstracts without consideration of outcomes. Studies were not assessed blindly. Disagreement between assessors was discussed until consensus was reached. When gender differences were analyzed without reporting on cortisol levels for boys and girls separately or when data were only presented in graphs, authors were requested for additional quantitative data. Data were stratified into two age groups: <8 years (prepubertal) and between 8 and 18 years (peri-/postpubertal). Ideally, stratification would have been based on pubertal staging according to Tanner. Unfortunately, only a minority of the included studies reported on the subjects’ Tanner stages. Because pubertal onset before age 8 years is considered to be pathologic [12], we chose 8 years as cut off for stratification. When articles reported on serial cortisol measurements, we included only data on the youngest assessment age. When cortisol levels were reported prepubertally as well as peri-/postpubertally within the same individual, we included one sampling moment for each stratified group. When articles reported on the same study population, we included the article with the lowest bias risk. When articles reported on dynamic tests of HPA axis activity, we only included baseline cortisol. We only included the control subjects of case-control studies. If known, we excluded female subjects on oral contraceptives. When gender differences were described but not quantified, the articles were included in the descriptive analysis rather than the meta-analysis.

### Meta-analysis

When necessary, we converted serum and salivary cortisol levels into nanomole per liter (nmol/L) and 24-h urine cortisol levels into microgram per 24 h (μg/24 h). When means ± SDs were not reported, the SD was calculated based on the following assumptions: the 95% CI is 3.92 SDs wide (2 × 1.96); the inter-quartile range is 1.35 SDs wide; the range is 4 SDs wide; the SD is the SE multiplied by the square root of the sample size [13]. To assess parametricity, we assumed that a normal distribution extends no more than 2 SDs from the mean [14], i.e., when normally distributed, the mean minus 2 SDs should be >0 nmol/L. Data analyses were performed using Review Manager (RevMan) version 5.3.5, 2014. For each study, the standardized mean gender difference (95% CI) in cortisol concentration was calculated by combining the SD with the sample size. Subsequently, fixed-effect meta-analyses were performed first, which assumes that the effect estimate of the group differences was fixed across studies. Second, the results of these analyses were compared with random-effects meta-analysis, which weigh studies of variable sample sizes more equally. We reported any source of bias from each included article conform the PRISMA statement and assessed selection, performance, detection, and other biases (Additional file 2). Bias was assessed as low, unclear, or high. A sensitivity analysis was done by excluding studies that had ≥1 high bias risks. Heterogeneity of the data was assessed by the *I* ^2^ statistic, with significance defined as *I* ^2^ > 50%. Publication bias was assessed through funnel plots.

## Results

**Fig. 1:**
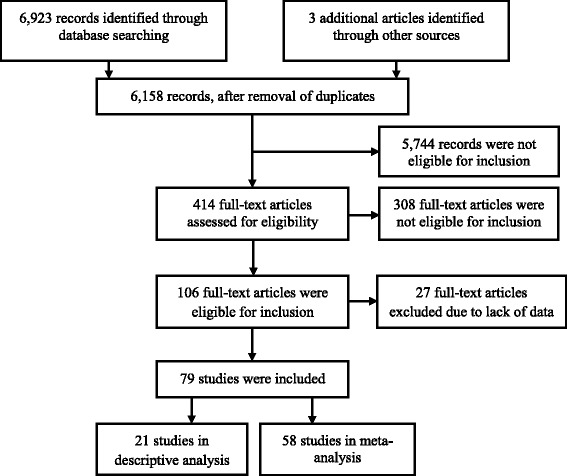
This flow chart presents the different phases of the systematic review and meta-analysis, conform the PRISMA-statement. (www.prisma-statement.org)

Figure 1 shows the flowchart of the descriptive analysis and meta-analysis. Of the 6158 titles and abstracts, 414 (7%) were eligible for full-text screening, from which 79 articles (19%) were included. Thirty-one authors of articles with insufficient quantitative data were contacted, of whom 12 responded: six provided the necessary quantitative data, five did not have access to the raw data anymore, and one was not willing to participate. Two articles reported the cortisol production rate assessed through 24-h serum sampling, which hampered inclusion in the meta-analysis. The authors of 27 articles that only provided gender-specific data in figures were contacted, but could not be reached. Subsequently, these articles were excluded. Finally, 21 articles were included only in the descriptive analysis, and 58 articles (with data on 16,551 subjects) had sufficient data for inclusion in the meta-analysis.

### Description of included studies

**Fig. 2:**
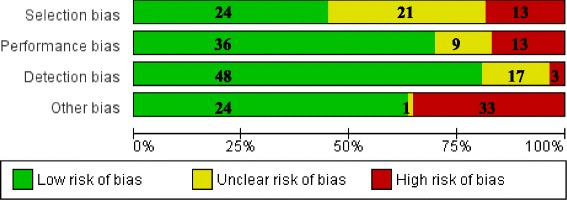
Risk of bias graph presenting a summary of the judgements of the accessors concerning risk of bias across all studies included in the meta-analysis. Bias risk is presented as percentage of total studies (*n* = 58)

Studies were conducted in Europe (*n* = 36), North America (*n* = 37), Asia (*n* = 3), South America (*n* = 2), or Africa (*n* = 1) and were published between 1973 and 2016. Sample sizes ranged from 11 to 2824 subjects, with seven studies having a sample size >500 subjects. Study designs were as follows: randomized placebo-controlled (*n* = 2), prospective observational (*n* = 29), non-randomized intervention, i.e., stress tests (*n* = 15), cross-sectional (*n* = 16), longitudinal (*n* = 11), and case-control (*n* = 6). All studies that assessed serum or salivary cortisol used immunoassays, except for one that used high-performance liquid chromatography (HPLC). Studies that assessed 24-h urine cortisol used immunoassays (*n* = 4), gas chromatography–mass spectrometry (*n* = 3), HPLC (*n* = 1), and liquid chromatography–UV detection (*n* = 1). Twenty-two studies (28%) did not collect morning samples, of which 11 did not report the time of collection and 11 described specifically that samples were collected in the afternoon. Additional file 3 presents the data extracted from the articles included in the meta-analysis. Three out of 21 studies (14%) included in the descriptive analysis had no high bias risk (Table 1), while 16 out of 58 studies (28%) included in the meta-analysis had no high bias risk (Fig. 2).

**Table 1:** Summary of studies included in the descriptive analysis

Group	First author (year)	*N* (% girls)	Age (years)	Sample protocol	Assay	Result	Bias^a^
Saliva <8 years	Klug (2000) [35]	119 (46%)	0	3-point day curve	Immunoassay	No gender differences	2
	Eiden (2015) [36]	257 (?)	0.75	Laboratory temperament assessment	Immunoassay	No gender differences	3
	Plusquellec (2011) [37]	466 (?)	1.6 ± 0. 1	Morning sample	Immunoassay	No gender differences	2
	Spinrad (2009) [38]	84 (49%)	4.5	Preschool laboratory assessment	Immunoassay	No gender differences	2
	Hatzinger (2007) [15]	102 (42%)	4.9 ± 0. 4	CAR	Immunoassay	Cortisol levels were lower in boys at awakening (*P* < 0.1)	1
Saliva 8–18 years	Safarzadeh (2005) [39]	100 (58%)	6–14	Morning sample	Immunoassay	No gender differences	1
	Isaksson (2015) [40]	68 (50%)	9	Morning sample	Immunoassay	No gender differences	2
	Kjölhede (2014) [41]	231 (50%)	9.5 ± 1.5	Morning sample	Immunoassay	No gender differences	1
	Vaillancourt (2008) [8]	154 (52%)	12.3 ± 0.8	Six samples standardized across time and day	Immunoassay	On Saturday morning, boys had significantly lower morning levels. On Monday and Thursday, no significant gender differences were found.	1
	Gunnar (2009) [42]	82 (49%)	9–15	TSST	Immunoassay	No gender differences	1
Serum <8 years	Fadalti (1999) [43]	72 (49%)	0–2	Morning sample	Immunoassay	No gender differences	0
	Ballerini (2010) [44]	319 (45%)	0–5	Surplus serum	Immunoassay	No gender differences	2
	Parker (1978) [45]	106 (43%)	2–12	Morning sample	Immunoassay	No gender differences	2
Serum 8–18 years	Kulasingam (2010) [46]	419 (?)	0 – 15	Surplus serum	Immunoassay	No gender differences	3
	Soldin (2005) [47]	376 (?)	0–18	Surplus serum	Immunoassay	No gender differences	1
	Karbasy (2015) [48]	711 (?)	0–19	?	Immunoassay	No gender differences	1
	Fadalti (1999) [43]	82 (49%)	6–18	Morning sample	Immunoassay	No gender differences	0
	Barra (2015) [49]	120 (45%)	12.4 ± 3	Morning sample	Immunoassay	No gender differences	1
	Chalew (1997) [50]	15 (73%)	12.7 ± 2.2	24-h blood withdrawal	Immunoassay	No gender differences	1
	Linder (1990) [51]	82 (58%)	8–17	24-h blood withdrawal	HPL	No gender differences.	0
Urine <8 years	–						
Urine 8–18 years	Dorn (1996) [52]	20 (55%)	15.2 ± 1.1	24-h urine sample	Immunoassay	No gender differences	1

^a^Number of high risks of bias out of four bias categories (selection, performance, detection, and other biases)

### Gender-specific differences

#### Descriptive analysis

Table 1 summarizes the data on the 21 studies included in the descriptive analysis. The majority (90%) of these studies reported no significant gender differences in cortisol levels. Before age 8 years, one study [15] found significantly lower salivary cortisol levels for boys at awakening. Between ages 8 and 18 years, one study [8] found significantly lower morning salivary cortisol levels in boys.

#### Meta-analysis

**Fig. 3:**
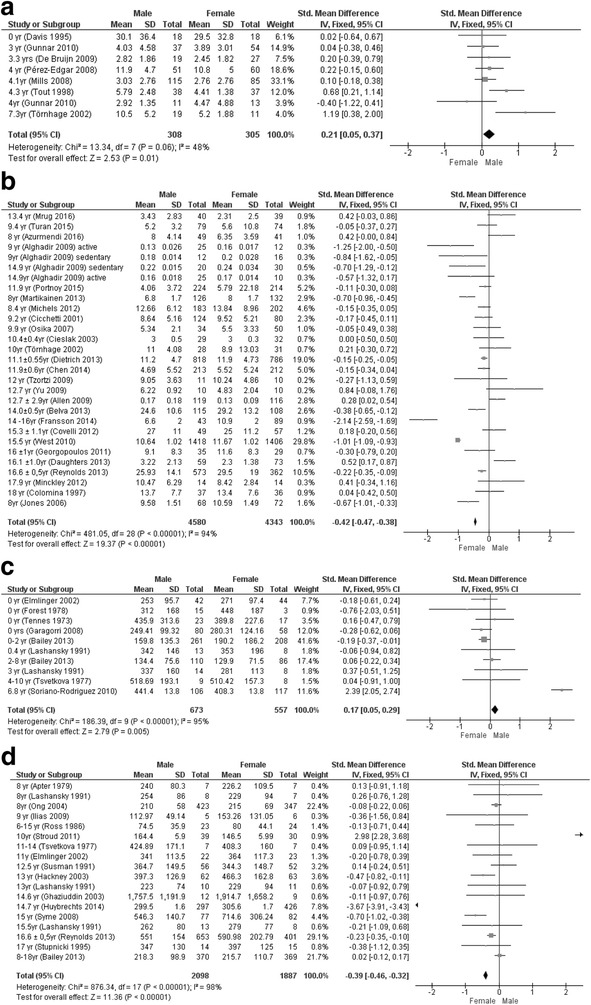
Forest plots of gender differences per subgroup. **a** Salivary cortisol (nmol/L) <8 years of age. **b** Salivary cortisol (nmol/L) 8–18 years of age. **c** Serum cortisol (nmol/L) <8 years of age. **d** Serum cortisol (nmol/L) 8–18 years of age

**Fig. 4:**
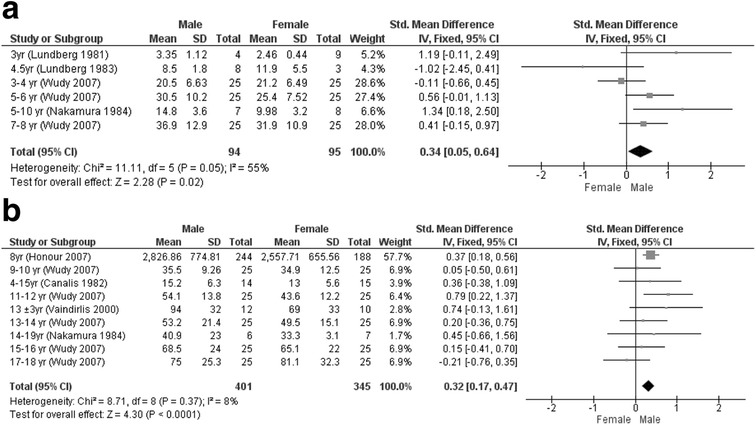
Forest plots of gender differences per subgroup. **a** 24-h urine cortisol (μg/24 h) <8 years of age. **b** 24-h urine cortisol (μg/24 h) 8–18 years of age

Nine articles (16%) did not report mean and SD values, which were therefore calculated. Figure 3 shows the results of the fixed-effect meta-analysis of serum and salivary cortisol. Compared to girls, boys <8 years had 0.21 (0.05; 0.37) nmol/L (*P* = 0.01, *I* ^2^ = 48%) higher salivary and 0.18 (0.06; 0.30) nmol/L (*P* < 0.01, *I* ^2^ = 94%) higher serum cortisol levels. Between ages 8 and 18 years, boys had 0.42 (0.38; 0.47) nmol/L (*P* < 0.01, *I* ^2^ = 94%) lower salivary and 0.34 (0.28; 0.40) nmol/L (*P* < 0.01, *I* ^2^ = 97%) lower serum cortisol levels. In contrast, free cortisol in 24-h urine was 0.34 (0.05; 0.64) μg/24 h (*P* = 0.02, *I* ^2^ = 55%) higher in boys aged <8 years and 0.32 (0.17; 0.47) μg/24 h (*P* < 0.01, *I* ^2^ = 8%) higher in boys between ages 8 and 18 years (Fig. 4). The sensitivity analyses did not significantly change the results, although it decreased the heterogeneity: boys <8 years had 0.40 (0.11; 0.69) nmol/L (*P* < 0.01, *I* ^2^ = 55%) higher salivary, 0.45 (0.30; 0.61) nmol/L (*P* < 0.01, *I* ^2^ = 94%) higher serum, and 0.28 (−0.04; 0.61) μg/24 h (*P* = 0.08, *I* ^2^ = 33%) higher 24-h urine cortisol; boys 8–18 years had 0.20 (0.13; 0.26) nmol/L (*P* < 0.01, *I* ^2^ = 47%) lower salivary, 0.10 (0.02; 0.18) nmol/L (*P* = 0.01, *I* ^2^ = 33%) lower serum, and 0.24 (0.02; 0.47) μg/24 h (*P* = 0.04, *I* ^2^ = 24%) higher 24-h urine cortisol.

Additional file 4 shows the results of the comparison between fixed-effect vs. random-effects meta-analyses. When analyzed by the random-effects method, the effect estimates of serum cortisol <8 years and between 8 and 18 years became non-significant (*P* = 0.46 and *P* = 0.62, respectively). This also applied to salivary cortisol <8 years (*P* = 0.06) and urinary cortisol <8 years (*P* = 0.12), although trends in the same direction were observed.

Funnel plots showed no evidence of publication bias (Additional file 5).

## Discussion

The results from this meta-analysis suggest that gender-specific differences in HPA axis activity are already present early in life. They also support previous observations which show that cortisol metabolism diverges between genders at pubertal age. Before age 8 years, cortisol in both serum and saliva was higher in boys compared to girls, at least in fixed-effect meta-analysis. These patterns were reversed after age 8 years. In contrast, gender differences in 24-h urine cortisol remained consistent with age, with higher cortisol levels in urine for boys before and after age 8 years (Additional file 6).

Total serum cortisol and free salivary cortisol reflect the balance between cortisol production and degradation, i.e., the bioavailability. Our meta-analysis suggests that puberty induces gender-specific changes in the bioavailability of cortisol, as reflected by similar changes in both total serum and free salivary cortisol levels, at least in fixed-effect models. Even though associations were absent for total serum cortisol in random-effect models, the change in free salivary cortisol could not be explained by an estrogen-induced increase in the production of CBG [4]. Moreover, the gender difference in cortisol in 24-hr urine (i.e., non-metabolized, free cortisol, representing cortisol production rate) remained consistent with age. Consequently, sex-hormone dependent effects on the hepatic metabolism of cortisol are more likely to explain our observations. Cortisol is metabolized reversibly by 11βHSD2, and irreversibly by α- and β-ring reductases and CYP3A. Animal studies showed a lower bioavailability of glucocorticoids in females due to decreased 11βHSD1 [16–18] and relatively increased 11βHSD2 activity [18], as compared to males. In addition, previous observations in humans suggest that estrogens could alter hepatic cortisol metabolism through increased CYP3A activity [19, 20] and decreased A-ring reduction [3, 21]. In contrast, sex-specificity in the activities of 11βHSD isozymes is debated in humans [3, 21, 22]. Since analyses of gender-specific differences in total serum cortisol were inconclusive in random-effects models (Additional file 4) and only one of the included studied had assessed CBG levels next to cortisol, we cannot exclude a gender-specific influence of CBG [4] on the serum cortisol level.

The HPA axis set point can be modified through an altered balance between mineralocorticoid and glucocorticoid receptor expression [23]. Animal studies have suggested that patterns in receptor expression develop in a gender-specific manner from birth onwards [24]. In humans, behavioral patterns that impact a child’s stress vulnerability have been associated with gender-specific changes in cortisol levels from age 1.5 years onwards [11, 25]. Therefore, even in our sample of normal children, gender-specific effects of stress exposure could be an explanation for our results [9].

Even subtle disturbances in HPA axis activity have been associated with cardiovascular disease and its risk factors [26–28]. Cardiovascular disease susceptibility is gender-specific [7, 29], which has been suggested to be due to gender differences in HPA axis activity, stress vulnerability, and responsivity [4, 30–32]. Early in life, developmental plasticity offers the child the capacity to change his HPA axis set point based on stress experiences [9, 33]. This ability offers opportunities to withstand early-life challenges, but it has also been suggested to affect disease risk later in life. Accordingly, although the gender differences found in our study were small, these patterns might contribute to gender-specific origins of health and disease [9].

The major strength of this study is our systematic approach and the effort to contact all authors of eligible publications, enabling us to include the data on 16,551 healthy children. Moreover, articles with a lack of quantitative data were included in our descriptive analysis with the aim to be as complete as possible. The large sample size enabled us to perform a sensitivity analysis, which decreased the heterogeneity between studies. Furthermore, we accounted for this heterogeneity by calculating standardized mean differences, based on the intervention effects relative to the variability observed [13]. Additionally, we chose fixed-effect meta-analysis, because the studies with a large sample size were most likely conducted with greater methodological accuracy [13]. Fixed-effect meta-analysis has the advantage of increasing the impact of large studies on the effect estimate. For comparison, results of random-effects meta-analyses, which put more weight on studies with small sample sizes, were also included (Additional file 4).

A limitation of this study is that only a subset of studies (16%) considered gender differences as the primary outcome. In addition, in 22 studies (28%), samples were not collected specifically during mornings. Both could have led to a selection or performance bias, which we accounted for in our sensitivity analysis. Furthermore, 21 articles with data on 3985 subjects could not be included in the meta-analysis due to lack of gender-stratified quantitative data, while most of these articles reported no significant gender differences. However, funnel plots of the articles included in the meta-analysis were not suggestive of publication bias. Instead, the plots seem to indicate that most articles reported on the nonexistence of gender differences, which might be a result of the common idea that gender differences are nonexistent at this early age. Nonetheless, our meta-analysis shows that significant gender differences are already present early in life. Another limitation is that almost all studies that reported on salivary or serum cortisol used immunoassays. Due to its superior specificity, liquid chromatography–tandem mass spectrometry is the method of choice for steroid hormone analysis [34]. Furthermore, we stratified studies based on the mean age or age range of the study group. Since study samples differed in age range, we have probably included some subjects <8 years of age in the 8–18-year groups, and vice versa. An overview of the age ranges of studies included in the meta-analysis is presented in Additional file 6. Moreover, only a minority of the included studies assessed Tanner’s pubertal staging. Therefore, we were unable to address the question at which maturational stage the direction of the gender-specific dimorphism in cortisol changes.

## Conclusions

In conclusion, gender differences in HPA axis activity are present early in life, with higher salivary cortisol concentrations in boys. A gender-specific evolution of cortisol metabolism is suggested to be induced by puberty, resulting in lower bioavailability of cortisol in boys. Although results from random-effects analyses were inconclusive for serum cortisol, the gender difference in cortisol production seems to be consistent between genders and age. Future research should take gender differences in HPA axis activity into account, regardless of age. Whether gender differences in stress-induced cortisol levels also exist is unknown and remains to be explored.

## Additional files

Additional file 1: Search strategy (DOCX 21 kb)
Additional file 2: Risk of bias of studies included in the meta-analysis (DOCX 191 kb)
Additional file 3: Extracted data of studies included in the meta-analysis (DOCX 2550 kb)
Additional file 4: Comparison fixed-effect analysis vs. random-effects analysis. (DOCX 202 kb)
Additional file 5: Funnel plots. (DOCX 65 kb)
Additional file 6: Overview of age ranges of studies included in meta-analysis. (DOCX 20 kb)

## Abbreviations

CBG: Corticosteroid-binding globulin
HPA axis: Hypothalamus–pituitary–adrenal axis
HPLC: High-performance liquid chromatography
PRISMA: Preferred Reporting Items for Systematic Reviews and Meta-Analysis
RevMan: Review manager
