# Supplementary material for: Gender-specific differences in hypothalamus–pituitary–adrenal axis activity during childhood: a systematic review and meta-analysis
Source: Biol Sex Differ. 2017 Jan 19;8:3. doi: 10.1186/s13293-016-0123-5 (PMC5244584; doi:10.1186/s13293-016-0123-5)
Supplement: Additional file 1: — Search strategy (DOCX 21 kb) [file 13293_2016_123_MOESM1_ESM.docx]

**Additional file 1: Search strategy**

| **Table S1A.** Search strategy for PubMed (14 January 2016) | | |
| --- | --- | --- |
| **Search** | **Query** | **Records (*n*)*** |
| **#1** | "Hydrocortisone"[Mesh] OR "Glucocorticoids"[Mesh] OR "11-beta-Hydroxysteroid Dehydrogenases"[Mesh] OR "Tetrahydrocortisone"[Mesh] OR "Tetrahydrocortisol"[Mesh] OR cortisol*[tiab] OR hydrocortison*[tiab] OR epicortisol*[tiab] OR cortifair*[tiab] OR cortril*[tiab] OR glucocorticoid*[tiab] OR beta hydroxysteroid dehydrogenase*[tiab] OR 11 oxoreductase*[tiab] OR 11 oxidoreductase*[tiab] OR 11 hydroxysteroid dehydrogenase*[tiab] OR 11b hydroxysteroid dehydrogenase*[tiab] OR 11 reductase*[tiab] OR 11beta hydroxysteroid dehydrogenase*[tiab] OR tetrahydrocortiso*[tiab] OR “hsd11b2”[tiab] OR “11bhsd2”[tiab] OR “11betahsd2”[tiab] OR 11beta hsd*[tiab] OR hydroxycortisol*[tiab] OR "Circadian Rhythm"[Mesh] OR “twenty four hour”[tiab] OR circadian*[tiab] OR diurnal*[tiab] OR nyctohemeral*[tiab] | **250,920** |
| **#2** | child*[tw] OR schoolchild*[tw] OR infan*[tw] OR adolescen*[tw] OR pediatri*[tw] OR paediatr*[tw] OR neonat*[tw] OR boy[tw] OR boys[tw] OR boyhood[tw] OR girl[tw] OR girls[tw] OR girlhood[tw] OR youth[tw] OR youths[tw] OR baby[tw] OR babies[tw] OR toddler*[tw] OR "Mental Disorders Diagnosed in Childhood"[MeSH] OR teen[tw] OR teens[tw] OR teenager*[tw] OR newborn*[tw] OR postneonat*[tw] OR postnat*[tw] OR perinat*[tw] OR puberty[tw] OR preschool*[tw] OR suckling*[tw] OR picu[tw] OR nicu[tw] OR "Arthritis, Juvenile"[Mesh] OR "Myoclonic Epilepsy, Juvenile"[Mesh] OR "Leukemia, Myelomonocytic, Juvenile"[Mesh] OR "Xanthogranuloma, Juvenile"[Mesh] OR "Juvenile Delinquency"[Mesh] OR "Corneal Dystrophy, Juvenile Epithelial of Meesmann"[Mesh] | **3,664,351** |
| **#3** | "Sex Characteristics"[Mesh] OR "Sex Factors"[Mesh] OR sex characteristic*[tiab] OR sex difference*[tiab] OR sex dimorphism*[tiab] OR sexual dimorphism*[tiab] OR sexual difference*[tiab] OR sexual characteristic*[tiab] OR sex factor*[tiab] OR sexual factor*[tiab] OR sexual dimorphi*[tiab] OR sex influenc*[tiab] OR sexual influenc*[tiab] OR gender*[tiab] | **451,894** |
| **#4** | (#1 AND #2 AND #3) | **2,643** |
| Mesh = Medical subject headings; tiab = words in title OR abstract; tw = words in title, abstract, MeSH and other content related fields | | |

| **Supplementary Table 1B.** Search strategy for Embase.com (14 January 2016) | | |
| --- | --- | --- |
| **Search** | **Query** | **Records (*n*)*** |
| **#1** | 'hydrocortisone'/exp OR 'glucocorticoid'/de OR '11beta hydroxysteroid dehydrogenase'/exp OR 'tetrahydrocortisone'/exp OR 'tetrahydrocortisol'/exp OR cortisol*:ab,ti OR hydrocortison*:ab,ti OR epicortisol*:ab,ti OR cortifair*:ab,ti OR cortril*:ab,ti OR glucocorticoid*:ab,ti OR ('beta hydroxysteroid' NEAR/3 dehydrogenase*):ab,ti OR (11 NEXT/1 oxoreductase*):ab,ti OR (11 NEXT/1 oxidoreductase*):ab,ti OR ('11 hydroxysteroid' NEXT/1 dehydrogenase*):ab,ti OR ('11b hydroxysteroid' NEXT/1 dehydrogenase*):ab,ti OR (11 NEXT/1 reductase*):ab,ti OR ('11beta hydroxysteroid' NEXT/1 dehydrogenase*):ab,ti OR tetrahydrocortiso*:ab,ti OR 'hsd11b2':ab,ti OR '11bhsd2':ab,ti OR '11betahsd2':ab,ti OR (11beta NEXT/1 hsd*):ab,ti OR hydroxycortisol*:ab,ti OR ('trier social stress' NEXT/1 test*):ab,ti OR tsst:ab,ti OR (stress NEAR/3 hormone*):ab,ti OR (stress NEAR/3 marker*):ab,ti | **235,221** |
| **#2** | adolescen*:ab,ti OR 'adolescence'/exp OR 'adolescent coping orientation for problem experiences'/exp OR 'adolescent development'/exp OR 'adolescent disease'/exp OR 'adolescent health'/exp OR 'adolescent parent'/exp OR 'adolescent pregnancy'/exp OR 'adolescent smoking'/exp OR 'adolescent'/exp OR 'adolescent-family inventory of life events and changes'/exp OR babies:ab,ti OR baby:ab,ti OR 'birth weight'/exp OR boy:ab,ti OR boyhood:ab,ti OR boys:ab,ti OR 'brazelton neonatal behavioral assessment scale'/exp OR 'child abuse'/exp OR 'child advocacy'/exp OR 'child behavior checklist'/exp OR 'child behavior'/exp OR 'child care'/exp OR 'child death'/exp OR 'child health care'/exp OR 'child health'/exp OR 'child nutrition'/exp OR 'child parent relation'/exp OR 'child psychology'/exp OR 'child restraint system'/exp OR 'child safety'/exp OR 'child welfare'/exp OR child*:ab,ti OR 'child'/exp OR 'childhood disease'/exp OR 'childhood mortality'/exp OR 'childhood'/exp OR girl:ab,ti OR girlhood:ab,ti OR girls:ab,ti OR 'high risk infant'/exp OR infan*:ab,ti OR 'infant disease'/exp OR 'infant mortality'/exp OR 'infant nutrition'/exp OR 'infant welfare'/exp OR 'infanticide'/exp OR 'infantile diarrhea'/exp OR 'infantile hypotonia'/exp OR 'juvenile delinquency'/exp OR neonat*:ab,ti OR 'neonatal weight loss'/exp OR 'newborn disease'/exp OR 'newborn morbidity'/exp OR 'newborn period'/exp OR newborn*:ab,ti OR 'newborn'/exp OR nicu:ab,ti OR 'only child'/exp OR paediatr*:ab,ti OR pediatri*:de,ab,ti OR 'pediatric advanced life support'/exp OR 'pediatric anesthesia'/exp OR 'pediatric cardiology'/exp OR 'pediatric hospital'/exp OR 'pediatric intensive care nursing'/exp OR 'pediatric nurse practitioner'/exp OR 'pediatric nursing'/exp OR 'pediatric rehabilitation'/exp OR 'pediatric surgery'/exp OR 'newborn hypoxia'/exp OR 'pediatric ward'/exp OR 'pediatrics'/exp OR perinat*:ab,ti OR 'perinatal development'/exp OR 'perinatal period'/exp OR 'persistent hyperinsulinemic hypoglycemia of infancy'/exp OR picu:ab,ti OR postnat*:ab,ti OR 'postnatal care'/exp OR 'postnatal development'/exp OR 'postnatal growth'/exp OR postneonat*:ab,ti OR preschool*:ab,ti OR puberty:ab,ti OR 'runaway behavior'/exp OR 'school child':ab,ti OR schoolchild*:ab,ti OR 'severe myoclonic epilepsy in infancy'/exp OR suckling*:ab,ti OR teen:ab,ti OR teenager*:ab,ti OR teens:ab,ti OR toddler*:ab,ti OR 'transient hypogammaglobulinemia of infancy'/exp OR youth:ab,ti OR youths:ab,ti | **4,477,134** |
| **#3** | 'sex difference'/exp OR 'sex ratio'/exp OR ('boy'/exp AND 'girl'/exp) OR (sex NEAR/3 characteristic*):ab,ti OR (sex NEAR/3 difference*):ab,ti OR (sex NEAR/3 dimorphism*):ab,ti OR (sexual NEAR/3 dimorphism*):ab,ti OR (sexual NEAR/3 difference*):ab,ti OR (sexual NEAR/3 characteristic*):ab,ti OR (sex NEAR/3 factor*):ab,ti OR (sexual NEAR/3 factor*):ab,ti OR (sexual NEAR/3 dimorphi*):ab,ti OR (sex NEAR/3 influenc*):ab,ti OR (sexual NEAR/3 influenc*):ab,ti OR gender*:ab,ti OR (boy*:ab,ti AND girl*:ab,ti) OR sex:ab,ti | **1,014,014** |
| **#4** | (#1 AND #2 AND #3) | **4,280** |
| /exp = EMtree keyword with explosion; /de = EMtree keyword without explosion; :ab,ti = words in title or abstract; NEXT/x = words in that order next to each other, x places apart; NEAR/x = words near to each other, x places apart | | |
