# Supplementary material for: Gender-specific differences in hypothalamus–pituitary–adrenal axis activity during childhood: a systematic review and meta-analysis
Source: Biol Sex Differ. 2017 Jan 19;8:3. doi: 10.1186/s13293-016-0123-5 (PMC5244584; doi:10.1186/s13293-016-0123-5)
Supplement: Additional file 2: — Risk of bias of studies included in the meta-analysis (DOCX 191 kb) [file 13293_2016_123_MOESM2_ESM.docx]

**Additional Tabel 2. Risk of bias of included studies**

**A.** Serum <8 yr **B.** Serum 8–18 yr **C.** Saliva <8 yr **D.** Saliva 8–18 yr **E.** Urine <8 yr **F.** Urine 8–18 yr

Risk of selection bias included: participants’ age range, and sex-specific differences in participation or baseline characteristics. Risk of performance bias included: time of sample collection, protocol transparency, and sex-specific differences in protocol compliance. Risk of detection bias included: sex-specific differences in assay methods. Non-parametric distribution of the data was recorded as a risk of other biases. Bias could be assessed as low (i.e., unlikely to alter the results), unclear (i.e., raises doubt about results) or high (i.e., weakens confidence in results).

| A. | | | | |
| --- | --- | --- | --- | --- |
|  | Selection bias | Performance bias | Detection bias | Other bias |
| Bailey 2013 | + | - | + | + |
| Elmlinger 2002 | ? | ? | + | + |
| Forest 1978 | ? | ? | + | + |
| Garagorri 2008 | + | + | + | + |
| Lashansky 1991 | ? | + | ? | + |
| Soriano-Rodriguez 2010 | + | ? | + | + |
| Tennes 1973 | ? | + | - | - |
| Tsvetkova 1977 | ? | + | + | + |
| Colored squares indicate: Green: low risk, Yellow: Unclear risk, Red: High risk of bias. | | | | |

| B. | | | | |
| --- | --- | --- | --- | --- |
|  | Selection bias | Performance bias | Detection bias | Other bias |
| Apter 1979 | ? | ? | + | - |
| Bailey 2013 | + | - | + | + |
| Elmlinger 2002 | ? | ? | + | + |
| Ghaziuddin 2003 | + | + | + | - |
| Hackney 200 | - | + | + | + |
| Huybrechts 2014 | - | + | + | + |
| Ilias 2009 | - | + | + | - |
| Lashansky 1991 | ? | + | ? | + |
| Ong 2004 | + | + | + | + |
| Reynolds 2013 | ? | + | + | + |
| Ross 1986 | - | - | + | - |
| Stroud 2011 | + | - | + | + |
| Stupnicki 1995 | - | - | + | + |
| Susman 1991 | ? | + | + | + |
| Syme 2008 | - | ? | ? | + |
| Tsvetkova 1977 | ? | + | + | + |
| Colored squares indicate: Green: low risk, Yellow: Unclear risk, Red: High risk of bias. | | | | |

| C. | | | | |
| --- | --- | --- | --- | --- |
|  | Selection bias | Performance bias | Detection bias | Other bias |
| Davis 1995 | ? | - | + | + |
| De Bruijn 2009 | + | - | + | - |
| Gunnar 2010 | + | + | ? | - |
| Mills 2008 | ? | - | + | - |
| Pérez-Edgar 2008 | + | + | + | + |
| Törnhage 2002 | ? | + | + | - |
| Tout 1998 | + | + | + | + |
| Colored squares indicate: Green: low risk, Yellow: Unclear risk, Red: High risk of bias | | | | |

| D. | | | | |
| --- | --- | --- | --- | --- |
|  | Selection bias | Performance bias | Detection bias | Other bias |
| Alghadir 2009 | + | + | + | - |
| Allen 2009 | + | - | + | + |
| Azurmendi 2016 | ? | + | - | - |
| Belva 2013 | + | + | + | + |
| Chen 2014 | ? | + | + | + |
| Cicchetti 2001 | - | + | + | - |
| Cieslak 2003 | + | - | + | + |
| Colomina 1997 | - | + | + | - |
| Covelli 2012 | - | + | + | - |
| Daughters 2013 | + | - | + | + |
| Dietrich 2013 | ? | ? | + | + |
| Fransson 2014 | + | + | + | - |
| Georgopoulos 2011 | - | ? | - | - |
| Jones 2006 | ? | + | + | + |
| Martikainen 2013 | - | + | + | + |
| Michels 2012 | + | + | + | + |
| Minckley 2012 | ? | + | + | ? |
| Mrug 2016 | - | + | + | - |
| Osika 2007 | + | + | + | - |
| Portnoy | + | - | + | - |
| Reynolds 2013 | ? | + | + | + |
| Törnhage 2002 | ? | + | + | - |
| Turan 2015 | + | - | ? | - |
| Tzortzi 2009 | + | + | + | + |
| West 2010 | - | + | + | - |
| Yu 2009 | ? | - | + | + |
| Colored squares indicate: Green: low risk,  Yellow: Unclear risk, Red: High risk of bias | | | | |

| E. | | | | |
| --- | --- | --- | --- | --- |
|  | Selection bias | Performance bias | Detection bias | Other bias |
| Lundberg 1981 | ? | + | + | - |
| Lundberg 1983 | ? | + | ? | - |
| Nakamura 1984 | - | ? | + | + |
| Wudy 2007 | + | + | + | + |
| Colored squares indicate: Green: low risk,  Yellow: Unclear risk, Red: High risk of bias | | | | |

| F. | | | | |
| --- | --- | --- | --- | --- |
|  | Selection bias | Performance bias | Detection bias | Other bias |
| Canalis 1982 | ? | ? | ? | + |
| Honour 2007 | + | + | + | - |
| Nakamura 1984 | - | ? | + | + |
| Vaindirlis 2000 | ? | + | ? | + |
| Wudy 2007 | + | + | + | + |
| Colored squares indicate: Green: low risk,  Yellow: Unclear risk, Red: High risk of bias | | | | |

| Alghadir 2015 [1] | | |
| --- | --- | --- |
| Risk of bias | Judgement | Support for judgement |
| Selection bias | Low risk | community based sample with clear exclusion criteria. |
| Performance bias | Low risk | clearly described protocol |
| Detection bias | High risk | “Cortisol levels (pg/ml)were measured in the saliva samples of participants using immunoassay technique. This was carried out according to the instructions of the cortisol ELISA kit (Diagnostics Biochem Canada, Inc.).” I emailed Dr. Gabr to check the unit of cortisol, since pg/mL is not often used and these salivary cortisol levels are very low. |
| Other bias | High risk | non-parametric distributed data. |

**Argumentation for our risk of bias judgement for each article separately, in alphabetical order**

| Allen 2009 [2] | | |
| --- | --- | --- |
| Risk of bias | Judgement | Support for judgement |
| Selection bias | Low risk | “Participants were recruited from the greater Los Angeles, California, area through mass mailings, posted advertisements, and classroom presentations.” Inclusion appears to be random therefore, though baseline characteristics are not described for males and females seperately |
| Performance bias | High risk | “Each pressure and heat pain task included 4 trials presented separately in counterbalanced order (setting and site of exposure) across participants.” “For the pressure and heat pain tasks, we used 2 anatomic sites, to avoid local sensitization or habituation, and we used 2 magnitudes of stimulus, to elicit greater variation in pain response (…)They were instructed to continue with each task for as long as they could” Nonetheless, time of collection was not described. |
| Detection bias | Low risk | “ Laboratory analysis was performed in a Worth-man laboratory. Quantitative determination of salivary cortisol was performed using an enzyme-linked immunosorbent assay kit (#1-0102/1-0112, Salimetrics), and blood spot cortisol was deter-mined by radioimmunoassay (Bio-Analysis Inc., Santa Monica, California).” |
| Other bias | Low risk | Cortisol levels are non-parametrically distributed, however it is a large sample size (bigger than 100 in each group) [3] |

| Apter 1979 [4] | | |
| --- | --- | --- |
| Risk of bias | Judgement | Support for judgement |
| Selection bias | Unclear risk | Initially 200 girls and 80 boys, 7-17 years old, took part, from which 140 girls and 67 boys took part in a second examination, and 44 boys in a third examination, at approximately one-year intervals. [5] Baseline characteristics of the participants are not described in this article, neither in Apter et al. 1978 [5] |
| Performance bias | Unclear risk | not described in this article, neither in reference 3 of Apter et al. 1978 [5] |
| Detection bias | Low risk | **“**The sample is first extracted with diethyl ether/ethyl acetate (1 : 1, by vol.), then chromatographed on a highly lipophilic derivative of Sephadex (hydroxyalkoxypropyl Sephadex, Lipidex) in light petroleum/chloroform (1 : 1, by vol.), and finally cortisol is measured by radioimmunoassay using a cortisol-21-BSA antiserum. ” [6] |
| Other bias | High risk | Table 1 and 2 give the mean concentration of all samples analyzed in age groups in a cross-sectional manner. |

| Azurmendi 2016 [7] | | |
| --- | --- | --- |
| Risk of bias | Judgement | Support for judgement |
| Selection bias | Unclear risk | little information is given on the subjects |
| Performance bias | Low risk | “Pearson correlations were performed to explore the relationship between the hormone levels obtained in sample A and sample B. Since positive correlations were found for all hormones, the means of the two values were calculated in order to obtain a single measure for each hormone and child at each age. “ |
| Detection bias | High risk | “All samples were assayed using an enzyme immunoassay kit (Salimetrics, State Collage, PA).” The unit of measurement of cortisol was not given. Dr. Azurmendi was e-mailed to confirm if it was μg/dL; no reply. |
| Other bias | High risk | non-parametric distribution. |

| Bailey 2013 [8] | | |
| --- | --- | --- |
| Risk of bias | Judgement | Support for judgement |
| Selection bias | Low risk | “Because the goal was to obtain samples from healthy infants and children, the recruitment of study participants took place in the wider community (schools, churches, community centers) in the multiethnic population of the greater Toronto area (...)additional samples from apparently healthy/metabolically stable children were collected from participants younger than 1 year to ensure a sufficiently large sample size. The samples for the group <14 days old were obtained from neonates in the maternity ward of Women’s College Hospital in Toronto who had been deemed healthy and were being sent home” [9]  “All samples analyzed were matched by age, sex, and ethnicity so as to generate equivalent groups for comparison and to produce an ethnically diverse group.“ |
| Performance bias | High risk | clearly presented study algorithm. However, timing of collection varied from 9:00h to 22:00h. |
| Detection bias | Low risk | “Serum samples for the aforementioned analytes were analyzed on the Abbott ARCHITECT i2000 system” |
| Other bias | Low risk | Cortisol levels are non-parametrically distributed, however it is a large sample size (bigger than 100 in each group) [3] |

| Belva 2013 [10] | | |
| --- | --- | --- |
| Risk of bias | Judgement | Support for judgement |
| Selection bias | Low risk | “Due to practical reasons (informed consent obtained after the scheduled visit or absence of children at the visit because of illness) only 223 children could be examined during the study period. Response rate varied between 50% (agreed/eligible; 278/553) and 68% (agreed/reached; 278/410). Demographic and clinical data of the refusals were comparable with those of the participating children” [11] |
| Performance bias | Low risk | “No difference in cortisol level was observed between samples obtained in spring and summer (8.9 µg/l) versus autumn and winter (8.4 µg/l) (p = 0.3).” “Adjustment for current characteristics, early life factors or maternal characteristics did not alter the results ( table 2 ).” “In 3 (3%) SC males the saliva sample was insufficient to assess the cortisol concentration.” |
| Detection bias | Low risk | **“**Salivary cortisol was measured by a commercial RIA for serum (GammaCoat TM Cortisol 125 I RIA)” |
| Other bias | Low risk | None |

| Canalis 1982 [12] | | |
| --- | --- | --- |
| Risk of bias | Judgement | Support for judgement |
| Selection bias | Unclear risk | Recruitment of subjects is not specified, other than that they were volunteers. Moreover, no baseline characteristics are known, and the subjects were from a wide age range, 4-15 years. |
| Performance bias | Unclear risk | Method of 24 hr urine collection not described |
| Detection bias | Unclear risk | "Cortisol was extracted from urine, chromatographically seperated, and identefied from its retention time as compared with cortisol standards (...) Cortisol was quantified by measuring its absorbance at 254 nm, as monitored in the effluent." |
| Other bias | Low risk | None |

| Chen 2014 [13] | | |
| --- | --- | --- |
| Risk of bias | Judgement | Support for judgement |
| Selection bias | Unclear risk | **“**Participants were recruited by advertisements within the city of Philadelphia and contiguous suburbs.” We do not know in which media they advertised. Concerning the baseline characteristics described in table 1: pubertal stage, BMI and income seem to differ between boys and girls. |
| Performance bias | Low risk | Study design extensively reported in Liu et al., 2013. [14]  **“**Of the 446 available participants, 21 had missing data on key measures (i.e., harsh discipline or saliva samples) and were therefore excluded from the analysis.” |
| Detection bias | Low risk | **“**Enzyme immunoassay (Salimetrics, State College, PA). ” |
| Other bias | Low risk | The mean and SD cortisol concentrations seems to be non-normally distributed and should be described in median and IQ ranges. However this is a large sample size. |

| Cicchetti 2001 [15] | | |
| --- | --- | --- |
| Risk of bias | Judgement | Support for judgement |
| Selection bias | High risk | “Non-maltreated low-income disadvantaged children (…) In order to obtain a demographically comparable comparison group, non-maltreated children were recruited from families receiving public assistance (…) The children with missing data did not differ from the larger group on any demographic indicators or cortisol variables.” Moreover, the age range of study subjects is likely to include both prepubertal and pubertal children: age 9.24 ± 2.33 yrs. |
| Performance bias | Low risk | “Cortisol assays were conducted without awareness of the maltreatment status of participating children.” |
| Detection bias | Low risk | “The saliva samples were assayed in duplicate using a high-sensitivity enzyme immunoassay (Salimetrics, State College, PA). In each assay batch, analytical controls representing low and high cortisol levels were included. The test has a lower limit of sensitivity of .007 µg/dL, and average intraassay and interassay coefficients of variation of 4.13 and 8.89, respectively. Method accuracy, determined by spike recovery, and linearity, determined by serial dilution, are 105% and 95%. ” |
| Other bias | High risk | Cortisol levels are non-parametrically distributed |

| Cieslak 2003 [16] | | |
| --- | --- | --- |
| Risk of bias | Judgement | Support for judgement |
| Selection bias | Low risk | “Subjects were recruited from three schools in Southwestern Ontario. Subjects came from classes of students from randomly selected schools that agreed to participate(...)Of the initially recruited subject cohort, 80% returned a signed parental consent form.” In addition, a clear overview of baseline characteristics was provided. |
| Performance bias | High risk | “All fifth grade students enrolled in the selected schools were provided with a project package containing a study description and a parental consent form.” Compliance to the protocol was not described, neither was timing of sampling. |
| Detection bias | Low risk | “Cortisol levels were assessed by using a DPC coat-a-count cortisol kit. Total plasma concentrations of cortisol were measured in duplicate by commercial solid-phase 125I radio-immunoassay kits. 125 I-labeled cortisol competes for anti-body sites for cortisol within the sample. The antibody is bound to the wall of the polypropylene tube, so when the supernatant is decanted, the antibody-bound fraction of the radiolabeled cortisol is still present. The amount of cortisol present in the sample is measured by a gamma counter.” |
| Other bias | Low risk | None |

| Colomina 1997 [17] | | |
| --- | --- | --- |
| Risk of bias | Judgement | Support for judgement |
| Selection bias | High risk | Subjects were part of a cohort that started at age 10. 579 subjects were then recruited, 304 agreed to participate at age 18. No baseline characteristics between affected and control groups, for example with regard to SES, education level, etc. were described. Though this cohort subsided from an area “with a rather high average socio-economic status.” |
| Performance bias | Low risk | All assessed in the same way |
| Detection bias | Low risk | “Salivary cortisol concentrations were determined using the “magic cortisol” RIA (Ciba-Corning, Gieben, Germany) modified by Kirschbaum, Strasburger et al. with a sensitivity of 0.1μg/dL. Each sample was measured in duplicate and averaged.” |
| Other bias | High risk | Cortisol levels are non-parametrically distributed |

| Covelli 2012 [18] | | |
| --- | --- | --- |
| Risk of bias | Judgement | Support for judgement |
| Selection bias | Low risk | "Participants were recruited from a historically African American high school (9^th^-12th grades) with student population of 1000, located in an urban, low socioeconomic community in Florida (...) One hundred sixteen students (77%) participated, and of these, 106 (92%) 49 males and 57 females completed the study (...) Students were representative of the general student population and not particular academic tracts." |
| Performance bias | Low risk | "All specimens were collected in the morning between 8 and 10 am (...) All specimens were collected in the morning between 8 and 10 am. On the day before testing, participants were instructed not to eat a major meal within 60 minutes before sample collection (...)Ten participants had incomplete data related to class or school attrition, and their data were excluded from analysis." |
| Detection bias | Low risk | **“**Cortisol was measured by radioimmunoassay using a polyclonal rabbit anticortisol antiserum. The assay is highly specific for cortisol in that the antiserum binds corticosterone 2.2% , 11-deoxycortisol 1.3 % , cortisone 0.6%, and progesterone 0.02% relative to cortisol. “ |
| Other bias | High risk | Cortisol levels are non-parametrically distributed |

| Daughters 2013 [19] | | |
| --- | --- | --- |
| Risk of bias | Judgement | Support for judgement |
| Selection bias | Low risk | “recruited via newspaper advertisements and letters sent to guardians of all high school students in the local county (...)The racial/ethnic background of participants were in line with the US Census Bureau statistics for Prince George’s County, Maryland (U.S. Bureau of the Census et al. 2010).” |
| Performance bias | High risk | Protocol clearly described. “Eighteen adolescents were excluded from analyses due to either the use of corticosteroids (n=14) or regular smoking in the past 30 days (n=4).” However samples were not collected in the morning: 3-5pm. |
| Detection bias | Low risk | “ Samples were analysed professionally off-site using salivary enzyme-immunoassay (EIA) technology by The University of Trier, Germany cortisol laboratory.” |
| Other bias | Low risk | None |

| Davis 1995 [20] | | |
| --- | --- | --- |
| Risk of bias | Judgement | Support for judgement |
| Selection bias | Unclear risk | 5 males were circumcised before testing (8-15 hours) |
| Performance bias | High risk | Time of collection varied widely: 8.30-19h, mean around 14.00h (1 to 3h after feeding) |
| Detection bias | Low risk | Serum and salivary cortisol was assayed, using Coat-a-Count Cortisol radioimmunoassay (trademark, Diagnostic Products Corporation). |
| Other bias | Low risk | None |

| De Bruijn 2009 [21] | | |
| --- | --- | --- |
| Risk of bias | Judgement | Support for judgement |
| Selection bias | Low risk | “ For the comparison group (prenatally nonexposed), children were selected whose mothers had at least given information in two separate periods during pregnancy and did not report high scores for any of the prenatal depression or anxiety questionnaires. ” In addition no important differences in baseline characteristics are found. |
| Performance bias | High risk | **“**Time of cortisol sampling may be a confounding variable (…)One-way ANOVA with time of home visit (divided into three groups: 10.00–12.00 a.m., 13.00–15.00 p.m., 15.00– 17.00 p.m.) as fixed factor and cortisol level at T1, T2, and T3, respectively, as dependent variable, revealed significant differences for girls in the prenatally exposed group.”  Initially 444/1093 women gave informed consent (41%).  In total 132 agreed for participation with the home visits and. Most important reasons for nonparticipation were lack of time (43%), personal difficulties (16%, e.g., illness or death of family member), problems with being videotaped (12%) and inability to contact some families because they had moved (8.9%)(…) Cortisol data were collected for 103 children (78%) (…) Lack of data was caused by insufficient saliva production of the child, or child’s refusal to suck on the cotton rolls, with younger children showing more refusal compared to older children. However, lack of data was equally represented within the two groups.” |
| Detection bias | Low risk | "time-resolved immunoassay with fluorescence detection” [22] |
| Other bias | High risk | Cortisol levels are non-parametrically distributed |

| Dietrich 2013 [23][ | | |
| --- | --- | --- |
| Risk of bias | Judgement | Support for judgement |
| Selection bias | Unclear risk | “After intensive recruitment efforts (including telephone calls, reminder letters and home visits),a total of 2230 children (76.0%) were included in the study at baseline.” [24] There are baseline differences between boys and girls, see table 2, in behavioral scores, which might influence cortisol levels. |
| Performance bias | Unclear risk | “Both the sampling and the preceding day should be normal school days, without special events or stressful circumstances (...) Only participants with complete morning cortisol assays (Cort1 and Cort2) were included in this study. In the population cohort complete cortisol data of 1667 children were available (…) Subjects were excluded from the analyses due to the use of corticosteroid-containing medication (population cohort: n = 22; clinic-referred cohort: n = 13), lack of compliance with the protocol (population cohort: n = 9; clinic-referred cohort: n = 35; note that non-compliant children from the clinic-referred cohort had higher YRS withdrawn-depressed scores than compliant children, t = 1.9, p = 0.05), and extreme cortisol values (>3 SD from the mean, population cohort: n = 32; clinic-referred cohort: n = 6). Lack of compliance was defined as failing to take the first sample within 5 min of awakening or the second sample between 25 and 35 min after awakening. This resulted in the following available morning cortisol  data: n = 1604” |
| Detection bias | Low risk | “A competitive solid phase time-resolved fluorescence immunoassay with fluorometric end point detection (DELFIA= dissociation-enhanced lanthanide fluorescent immunoassays" [25] |
| Other bias | Low risk | None |

| Elmlinger 2002 [26] | | |
| --- | --- | --- |
| Risk of bias | Judgement | Support for judgement |
| Selection bias | Unclear risk | Not described |
| Performance bias | High risk | Protocol, including time of sampling, not described |
| Detection bias | Low risk | “Immulite and Immulite 2000® chemiluminescence assay system” |
| Other bias | Low risk | None |

| Forest 1978 [27] [27 | | |
| --- | --- | --- |
| Risk of bias | Judgement | Support for judgement |
| Selection bias | Unclear risk | not described |
| Performance bias | Unclear risk | test procedures clearly described. However, compliance to protocol is not described |
| Detection bias | Low risk | “assayed by a competitive protein-binding method” |
| Other bias | Low risk | none |

| Fransson 2014 [28] [ | | |
| --- | --- | --- |
| Risk of bias | Judgement | Support for judgement |
| Selection bias | Low risk | “The total population included 545 adolescents. Of these, 413 (246 girls and 167 boys) completed an initial questionnaire that was mandatory for inclusion in the cortisol study (...) A comparison of self-rated health and re-current pain shows no significant differences between the analytic sample and those responding to the questionnaire regarding self-rated health (analytic sample: M = 1.86, SD= .69; others: M = 1.82, SD= .74), or recurrent pain (analytic sample: M =1.96, SD = 2.18; others: M = 1.77, SD = 2.14). ” |
| Performance bias | Low risk | Procedures described in detail, leading to a uniform approach for the participants. “adolescents (n = 15) taking their first sample more than 5 minutes after waking were excluded” |
| Detection bias | Low risk | “Cortisol was determined using competitive radioimmunoassay (Spectria Cortisol RIA, Orion Diagnostica, Espoo, Finland; intra-assay preci- sion <5%, 1.7–4.1% and inter-assay precision <10%, 4.3– 9.0%). Each sample was analyzed twice and in randomized order “ |
| Other bias | High risk | It is not clear if the presented results are the natural logartithm or the 10log. |

| Garagorri 2008 [29] | | |
| --- | --- | --- |
| Risk of bias | Judgement | Support for judgement |
| Selection bias | Low risk | Selection and exclusion criteria in detail described. Besides the fact that participation and baseline characteristics are not sex-specifically analysed, we expect no major differences. |
| Performance bias | Low risk | “Umbilical cord blood was obtained by needle aspiration at delivery immediately after clamping the cord with the placenta still in situ. The remaining blood samples were obtained from each infant between 8:00 am and 9:00 am by rapid venous puncture six times during the first 6 months of life: on the 3rd, 15th, 30th, 60th, 90th, 120th, 150th and 180th days of life.” Besides the fact that compliance to the protocol is not described, we expect no major sex-specific differences. |
| Detection bias | Low risk | **“**All steroid determinations were performed in a single laboratory (Biochemistry Laboratory from the University Clinical Hospital ‘Lozano Blesa’, Zaragoza, Spain) using specific RIA procedures (…) The direct RIA assays were performed on plasma aliquots as described by the manufacturers using (…) the 125I-Gamma Coat Cortisol RIA kit from Baxter (Deerfield, IL, USA)” |
| Other bias | Low risk | None |

| Georgopoulos 2011 [30] | | |
| --- | --- | --- |
| Risk of bias | Judgement | Support for judgement |
| Selection bias | High risk | "Controls recruited from public schools in Greece (...) All baseline characteristics are reported of both cases and controls. However: girls 16.0±1.4 years; boys 15.3±2.0 years.  Body fat percentage: girls 27.7±6.91 (n=39), boys 20.8±4.7 (n=40) % Lean body mass: girls 37.8±5.14 (n=38), boys 48.1±9.9 (n=40) kg" |
| Performance bias | Unclear risk | Little is described: “From controls, the saliva samples were collected in the morning.” |
| Detection bias | High risk | Salivary cortisol concentrations were measured by electro-chemiluminescence quantitation (Elecsys 2010, Roche Diagnostics, Tokyo, Japan) with dynamic range: 0.04–63μg/dl. Sensitivity was 0.07μg/dl. The intra- and interassay CVs were 5.7% and 5.6%,respectively. |
| Other bias | High risk | Cortisol levels are non-parametrically distributed |

| Ghaziuddin 2003 [31] | | |
| --- | --- | --- |
| Risk of bias | Judgement | Support for judgement |
| Selection bias | Low risk | “Participants were recruited from newspaper advertisements and flyers posted throughout a university hospital (...) The females and males were similar in age, Tanner rating and SES. There was, however, a significant difference between males and females in the season of testing (Fisher’s exact test, p=0.015): the majority of the females (n=7, 77.7%) underwent the challenge procedure in the fall; for males, the most frequent time was the summer (n=6, 54.5%). Males and females were similar in all behavior ratings.” |
| Performance bias | Low risk | ”All female participants were studied during the first 14 days of their menstrual cycle(…) All participants were asked to adhere to a low- monoamine diet for approximately 48 h prior to the study.” In addition, no cortisol assessment was undertaken for one female (in total N=8), no explanation was given. |
| Detection bias | Low risk | “Cortisol was measured in ethanol extracts of plasma samples by a standard competitive protein-binding method using human cortisol-binding globulin. ” |
| Other bias | High risk | The attrition rate is not described during the longitudinal follow-up. Moreover, cortisol levels are non-parametrically distributed |

| Gunnar 2010 [32] | | |
| --- | --- | --- |
| Risk of bias | Judgement | Support for judgement |
| Selection bias | Low risk | While baseline characteristics are not sex-specifically analysed, we expect no major differences. In addition, **“**of the parents contacted, 59% agreed to participate (...)  Recruitment took place over multiple years, implying that children from the same site were not necessarily present at the same time in the child-care setting. Of the 30 sites with multiple participants, 36 of the 70 participants were present together at the same time. Sites contributing more than one child to the analyses tended to be the larger child-care sites, t = 1.71, df = 149, p<.10.” |
| Performance bias | Low risk | “The children were observed on two mornings between 8:30 and 9:30 am on days the provider deemed typical (i.e., no field trip, birthday parties, etc.) (...)Twenty children were removed prior to assaying saliva for cortisol because the care provider deviated by more than 1.5 hr in the timing of nearly all of their samples, resulting in the child having no usable cortisol data” Attrition is not sex-specifically analysed, though we expect no major differences. |
| Detection bias | Unclear risk | Assays were conducted in duplicate using a time-resolved fluorescence immunoassay.” not further described. I emailed Dr. Gunnar for more information. She did not respond |
| Other bias | High risk | Cortisol levels are non-parametrically distributed |

| Hackney 2003 [33] | | |
| --- | --- | --- |
| Risk of bias | Judgement | Support for judgement |
| Selection bias | High risk | “This segment of the Cardiovascular Health in Children study was conducted in 12 schools in North Carolina (…) Initially 108 schools agreed to participate; study schools were randomly selected from 33 of those that met study criteria as clearly rural or urban.”  “Baseline characteristics differed sex-specifically, see table 1: boys and girls differ significantly in pubertal stage, sum skinfolds, physical activity score, caloric intake, VO_2 max_, leptin and cortisol level.” [34] |
| Performance bias | Low risk | “All physiologic measurements were collected “on-site” at the subjects’ schools. All physical tests and research procedures were completed in the same order, which was (1) resting blood collec- tion, (2) questionnaires, (3) height, (4) weight, (5) skinfold measurements, and (6) PWC195 cycle ergometry test. All blood samples were obtained after an overnight fast (8 h) by using venipuncture techniques performed by a trained phlebotomist.” Compliance to protocol was not described. |
| Detection bias | Low risk | “Plasma leptin, cortisol, thyroid stimulating hormone (TSH), and free triidothyronine (T3) concentrations were assessed in duplicate determination by using commercially available highly specific radioimmunoassay procedures. The reagents for the leptin assays were obtained from Linco Inc. (St Charles, MO, USA), and all other hormonal reagents were provided by Diagnostic Products Corporation Inc. (Los Angeles, CA, USA)” |
| Other bias | Low risk | None |

| Honour 2007 [35] | | |
| --- | --- | --- |
| Risk of bias | Judgement | Support for judgement |
| Selection bias | Low risk | “Comparison with U.K. 1990 growth references show that this Children in Focus cohort is representative in terms of birth weight (mean U.K. reference sd score= 0.0, sd = 1.0) (...) On the basis of normal birth weights, few children from pregnancies complicated by preeclampsia, gestational diabetes and small for gestational age (SGA) were likely to have been included in the group”. In addition, see table 1: no evident differences between males and females in baseline characteristics. |
| Performance bias | Low risk | “Collections were judged to be reliable when total volumes were between 300 and 750 ml and the creatinine concentrations were within the range 3.5 to 15 mmol/ liter. Other samples were not processed for steroid excretion rates due to the likelihood of incomplete collections.” 461 children collected urine. In total 244 boys and 188 girls completed the protocol. (94%) |
| Detection bias | Low risk | “gas chromatography after solid phase extraction, enzyme hydrolysis of conjugates, and derivative formation (methyloxime-trimethylsilyl ether). “ |
| Other bias | High risk | 14h urine collections are difficult to interpretate, and conversion to 24h production rates less reliable. Moreover, IQ ranges instead of standard deviations were given. |

| Huybrechts 2014 [36] [ | | |
| --- | --- | --- |
| Risk of bias | Judgement | Support for judgement |
| Selection bias | High risk | “ Adolescents were selected by random cluster sampling [all pupils from a selection of classes from all schools in 10 European cities(...) Blood sampling was performed in a randomly representative sample of one third of the participants (100 adolescents per city), based on class level. “ Note, chronic health problems were not an exclusion criteria. Prevalence of those is not described in the article. Moreover body fat percentage and pubertal stage at baseline differed significantly between boys and girls (see table 1) |
| Performance bias | Low risk | Vary concise set up. [37] **“**Of the 1089 eligible adolescents with valid blood results, 723 adolescents provided all necessary data for the research purposes  (...) When comparing the 1089 adolescents of HELENA-CSS having valid blood results with the 723 adolescents included in the present study, no significant differences were observed for age, BF% or the blood parameters leptin, insulin, glucose and cortisol.” |
| Detection bias | Low risk | ”Total cortisol concentrations were measured using the Fluorescence PolarizationImmunoassay technology (AxSYM, Abbott Laboratories, USA)” |
| Other bias | Low risk | None |

| Ilias 2009 [38] | | |
| --- | --- | --- |
| Risk of bias | Judgement | Support for judgement |
| Selection bias | High risk | **“**Prepubertal children with idiopathic short-normal stature and normal GH responses to at least one pharmacological stimulation test (peak GH >10 ng/ml after arginine or clonidine administration) as well as normal IGF-I, IGF-binding protein-3, and 24-h integrated GH plasma concentrations were studied.“ However age, bone age and BMI seem to be similar in boys and girls (table 1). |
| Performance bias | Low risk | The protocol is clearly described. Protocol compliance is not described, but due to the clinical setting of the study, it can assumed to be good. |
| Detection bias | Low risk | **“**Commercial kits were used for the measurement of serum concentrations of F (RIA, Radim, Pomezia, Italy)” |
| Other bias | High risk | Cortisol levels are calculated from a 24h withdrawal and non-parametrically distributed |

| Jones 2006 [39] | | |
| --- | --- | --- |
| Risk of bias | Judgement | Support for judgement |
| Selection bias | Unclear risk | Selection criteria and baseline characteristics were not described. While in the study published previously 210 neonates were eligible, while in this 140 children took part.[40] |
| Performance bias | Low risk | Clear described protocol. “Parents were asked to change their appointment if stressful events or illness occurred in their family in the preceding week. “ |
| Detection bias | Low risk | **“** Salivary cortisol concentrations were measured using a time-resolved immunofluorescent assay (DELFIA) “ |
| Other bias | Low risk | None |

| Lashansky 1991 [41] | | |
| --- | --- | --- |
| Risk of bias | Judgement | Support for judgement |
| Selection bias | Unclear risk | Selection criteria and baseline characteristics were not described, except for “All patients were examined by a single examiner (T.G.) to ensure that all children were healthy, their nutritional status was adequate, their growth was appropriate” Moreover, pubertal status was clearly described for all age groups. |
| Performance bias | Low risk | Clear description of procedures. “All tests were performed between 0800-1000 h” |
| Detection bias | Unclear risk | “Cortisol and DHEAS were measured directly by RIA in diluted serum using specific antisera.” |
| Other bias | Low risk | None |

| Lundberg 1981 [42] | | |
| --- | --- | --- |
| Risk of bias | **Judgement** | **Support for judgement** |
| Selection bias | Unclear risk | “Fifty women, each of whom was expecting her first child and was experiencing a normal pregnancy, had been selected for a longitudinal study. This investigation represents part of that (…) only 15 families were able to participate in this part of the study.” No baseline characteristics of these 15 families are described. |
| Performance bias | Low risk | The procedure for urine sampling was clearly described. ”In the at-home session, they were also asked to avoid stressful activities and physical exercise, and to keep a brief diary of their activities (…) According to the diaries, the child usually spent the day playing, while the mother and the father relaxed (…) In some cases, the whole family went shopping at some point during the day.” |
| Detection bias | Low risk | “Radioactivity was measured in a Packard liquid scintillation spectrometer, using “Aquasol” (New England Nuclear) as the scintillation solution.” (42) |
| Other bias | High risk | overnight urine collections are difficult for interpretation, and conversion to 24h production rates less reliable. |

| Lundberg 1983 [44] | | |
| --- | --- | --- |
| Risk of bias | Judgement | Support for judgement |
| Selection bias | Unclear risk | Selection criteria were not described. Baseline characteristics (Age, weight and height) were similar among boys and girls |
| Performance bias | Low risk | "The method used for urine sampling was designed so as not to induce emothional arousal in the child (...) In order to make the children familiar with the procedure, the sampling of urine (not used in the analyses) was started one week prior to the actual study (...) Eleven joined the center when the study started. According to consistent reports from the parents and nurses, all the eleven children were well adjusted to the day-care situation by the end of their second week." |
| Detection bias | Unclear risk | Method of analysis not described |
| Other bias | High risk | Although, "The actual day of measurement was randomized between children and no measurements were taken on days when the children participated in special activities with the nurces", urine collections were done for each child during different time windows (differing in length) and are therefore difficult to interpretate. Conversion to 24h production rates makes them less reliable. |

| Martikainen 2013 [45] | | |
| --- | --- | --- |
| Risk of bias | Judgement | Support for judgement |
| Selection bias | High risk | "Because a primary objective of the initial study was to examine the effects of maternal licorice consumption during pregnancy on their offspring's developmental outcomes, subsequent participants were recruited to overrepresent children whose mothers consumed higher amounts of licorice (...) of the 413 invited children, 321 (77.7%) participated in the 2006 follow-up" |
| Performance bias | Low risk | Clearly described protocol. Children who did not provide at least 4 days of valid physical activity data including at least 1 weekend day (N=54) or children who had more than 1 missing cortisol value were excluded. |
| Detection bias | Low risk | "Salivary cortisol concentrations were determined by use of a competitive solid-phase, time-resolved fluorescence immunoassay with fluorometric end point detection (DELFIA; Wallac, Turku, Finland)" |
| Other bias | Low risk | None |

| Michels 2012 [46] | | |
| --- | --- | --- |
| Risk of bias | **Judgement** | **Support for judgement** |
| Selection bias | Low risk | Subjects included in this article are from a sub-study of the IDEFICS, named the ChiBS. The IDEFICS study:“All children in the defined age group who resided in the defined regions and who attended the selected primary schools (grades 1 and 2), preschools or kindergartens were eligible for participation. “(Ahrens et al. 2011) the ChiBS study: “All children participating in the control section of this IDEFICS survey (N=761) were eligible to join the ChiBS study.” [47]  In addition, personal and sociodemographic characteristics did not differ between boys and girls. |
| Performance bias | Low risk | “the children were asked to sample when healthy and on a normal day; to strictly respect the time points; not to eat, drink or brush their teeth in the hour before collection; to avoid physical activity 2h before sampling; and to avoid caffeine-rich drinks and minimize medication on the sampling days. The parents were also asked to fill in a checklist about instruction compliance." |
| Detection bias | Low risk | “Salivary cortisol was assayed within the first month after collection in the routine laboratory of the Ghent University Hospital on a Modular E 170 immunoanalyser system (Roche Diagnostics, Mannheim, Germany) using the Roche Cobas Cortisol assay.” A competitive electrochemiluminescence immunoassay (ECLIA) |
| Other bias | Low risk | Cortisol levels are non-parametrically distributed, however it is a large sample size (bigger than 100 in each group) [3] |

| Mills 2008 [48] | | |
| --- | --- | --- |
| Risk of bias | Judgement | Support for judgement |
| Selection bias | Unclear risk | “families were recruited through a letter of invitation under a cover letter sent by a government agency responsible for administering health care (…) The recruitment procedure did not allow the computation of a response rate” |
| Performance bias | High risk | “To accommodate parents’ schedules, we scheduled laboratory sessions any day of the week at different times of day (between 9:00 am and 7:00 pm) (...) 4 families subsequently withdrew from the study; and data were missing for 11 children" |
| Detection bias | Low risk | “Samples were assayed in duplicate without dilution (25 μl) using an enzyme immunoassay kit (High Sensitivity Salivary Cortisol, Catalog No. 1-0102/1- 0112; Salimetrics; State College, PA). “ |
| Other bias | High risk | Cortisol levels are non-parametrically distributed |

| Minkley 2012 [49] | |  |
| --- | --- | --- |
| Risk of bias | Judgement | Support for judgement |
| Selection bias | Unclear risk | Selection procedure was not described, besides “All of the 93 participants were students of biology courses at secondary or comprehensive schools (n=13) in Bochum or nearby cities (...) there was no significant intergroup difference in the distribution according to sex, age, or BMI (p>0.05)" |
| Performance bias | Low risk | “we have assessed their average awakening time which was between 6 AM and 7 AM. Thus, they were awake for at least 4 h before we collected the saliva(…) Furthermore, we aimed to test the students in relatively standardized conditions (same location, time of the day, tutor, information, etc.) (...) 4 students were excluded because of blood contamination or an insufficient amount of saliva for cortisol measurement.” |
| Detection bias | Low risk | “Cortisol levels were quantified using the cortisol saliva immunoassay kit from DRG (Marburg, Germany). Analyses were carried out using a 96-well ELISA reader (Thermo Fisher, Vantaa, Finland).” |
| Other bias | Unclear risk | None |

| Mrug 2016 [50] | | |
| --- | --- | --- |
| Risk of bias | Judgement | Support for judgement |
| Selection bias | High risk | Low socioeconomic class and “from approximately 240 invited students, 129 (54%) provided their contact information and 84 of those (65%) completed the interview (recruitment was curtailed by limited resources)” |
| Performance bias | Low risk | Clearly described protocol. |
| Detection bias | Low risk | “Cortisol was assayed in duplicate by commercially available enzyme immunoassay without modification to the manufacturers recommended protocol (Salimetrics, Carlsbad, CA).” |
| Other bias | High risk | Non-parametric distribution of the data. |

| Nakamura 1984 [51] | | |
| --- | --- | --- |
| Risk of bias | Judgement | Support for judgement |
| Selection bias | High risk | Selection procedure and baseline characteristics are not described. Moreover, age range of the participants is wide in relation to the small sample size. |
| Performance bias | Unclear risk | Protocol of 24h urine collection is not described |
| Detection bias | Low risk | ”Urinary cortisol was measured in each sample by a high-performance-liquid-chromatographic method.” |
| Other bias | Low risk | None |

| Ong 2004 [52] | | |
| --- | --- | --- |
| Risk of bias | **Judgement** | **Support for judgement** |
| Selection bias | Low risk | "851 (...) from these 2 subcohorts attended a research clinic and gave a fasting blood sample (...) at age 8 yr. Of these, 770 subjects had sufficient plasma samples for adrenal androgen assays (...) These children did not differ from other ALSPAC children with regard to body size at birth or during childhood." In addition, see table 1: age, weight and waist circumference did not differ between boys and girls. |
| Performance bias | Low risk | Protocol is clearly described and "data were excluded on children (...) who admitted to be nonfasting on the questionairre (n=2)" |
| Detection bias | Low risk | "Cortisol was measured by immunochemiluminescence (Cortisol ELISA assay, DSL)" |
| Other bias | Low risk | Cortisol levels are non-parametrically distributed, however it is a large sample size (bigger than 100 in each group) [3] |

| Osika 2007 [53] | | |
| --- | --- | --- |
| Risk of bias | Judgement | Support for judgement |
| Selection bias | Low risk | "In some of the classes the teachers expressed hesitation to engage in the study due to lack of time, despite the commitment made by the principals, and this is likely to have influenced the participation rate" In addition "seven (...) children dropped out because one had a somatic disease and the remaing six changed their minds about participating" Nonetheless, see table 1: no sex-specific differences in demographic and antropometric data |
| Performance bias | Low risk | "samples were all colected in a standardized manner (...) there was no notable difference between the children with complete and incomplete cortisol data (...) between the sexes" |
| Detection bias | Low risk | Salivary cortisol concentrations were determined using a commercial, radioimmunoassay (Spectria CORTISOL [125I] Coated Tube Radioimmunoassay, Salivette; Sarstedt Inc., Rommelsdorf, Germany) |
| Other bias | High risk | Cortisol levels are non-parametrically distributed. Moreover, calculating a SD from a range is not a robust method. |

| Pérez-Edgar 2008 [54] | | |
| --- | --- | --- |
| Risk of bias | Judgement | Support for judgement |
| Selection bias | Low risk | "Preliminary analyses found no significant differences between the children in this study and the remaining cohort with missing data (all p's >0,12)"  "This sample is unusually homogeneous with respect to good health of the mother and infant as well as the social class and economic advantage of the family" [55] |
| Performance bias | Low risk | Detailed description of procedures, including cortisol sample collection times, as well as analyses of gender differences of collection times (non-significant). Compliance to protocol was not described |
| Detection bias | Low risk | "Concentrations were determined using a solid phase radioimmunoassay (125I) (Coat-A-Count, Diagnostic Products Corporation, Los Angeles, CA), using 200 uL of saliva". |
| Other bias | Low risk | None |

| Portnoy 2015 [56] | | |
| --- | --- | --- |
| Risk of bias | Judgement | Support for judgement |
| Selection bias | Low risk | “The original sample consisted of 454 subjects. Of this original group, eight subjects were later deemed ineligible or withdrew, resulting in a sample of 446 subjects. The sample was 50.6% male. The racial makeup of the sample  was 11.9% white, 79.7% African American, and 4.8% multiracial. Less than 1% of the sample identified as Hispanic, Asian, or Native American. The mean age of the sample was 11.92 years (SD = .59). 14.2% of subjects had a lifetime diagnosis of conduct disorder and 19.1% had a lifetime diagnosis of oppositional defiant disorder” |
| Performance bias | High risk | Stress test was performed in the afternoon. |
| Detection bias | Low risk | “Samples were assayed for salivary cortisol using a commercially available enzyme immunoassay (Salimetrics, State College, PA). The test used 25 μL of saliva for singlet determinations and had a range of sensitivity of .007–3 μg/dl. Samples were assayed in duplicate and the averages of cortisol concentrations were used in the current analysis.” |
| Other bias | High risk | Data are non-parametrically distributed |

| Reynolds 2013 [57] | | |
| --- | --- | --- |
| Risk of bias | **Judgement** | **Support for judgement** |
| Selection bias | Unclear risk | "The 17-year follow-up (...) included 1771 Raine participants, representing 61,8% of the orginal cohort" |
| Performance bias | Low risk | Procedures clearly described. Moreover, "the date and time of blood collection before 1000 h in the morning was documented (...) The participants recorded the date and time of collection before 10 am in the morning on sample tubes" |
| Detection bias | Low risk | “Cortisol and CBG were measured by125I radioimmunoassay (GammaCoat cortisol RIA — DiaSorin, MN, USA and CBG-RIA-100 — BioSource Europe S.A., Belgium(…) The intra- and interassay variations of 23 cortisol RIAs were 8% and 14.0% (salivary cortisol), 8.8% and 13.7% (plasma cortisol)” |
| Other bias | Low risk | Salivary cortisol levels are non-parametrically distributed, however it is a large sample size (bigger than 100 in each group) [3] |

| Ross 1986 [58] | | |
| --- | --- | --- |
| Risk of bias | **Judgement** | **Support for judgement** |
| Selection bias | High risk | Selection procedures are not described. Baseline characteristics only scarce. Distribution of Tanner staging seem to be equally distributed between both sexes. However, the age range of participants is wide and is likely to include both prepubertal and pubertal children: age 6-15 yrs. |
| Performance bias | High risk | protocol described in detail. Compliance to protocol not described, but due to the clinical setting of the study, it can be assumed to be good. However plasma was not a morning sample, collected between 1900 and 2000h. |
| Detection bias | Low risk | Plasma cortisol was measured by RIA, as clearly described in Kao et al. 1975 [59] |
| Other bias | High risk | Cortisol levels are non-parametrically distributed |

| Soriano-Rodriguez 2010 [60] | | |
| --- | --- | --- |
| Risk of bias | Judgement | Support for judgement |
| Selection bias | Low risk | Selection and participation is only described by "Subjects (...) were consecutively recruited among those seen for well-child check-up visits by their primary care paediatricians (...) Participation ranged from 50 to 70% among the different clinics." Nonetheless, baseline characteristics, presented in table 1, show no significant differences between boys and girls. |
| Performance bias | Unclear risk | Extensively described method. Though, compliance to protocol is not described. |
| Detection bias | Low risk | "Cortisol was measured by a fluorescence polarization immunoassay (FPIA; AxSYM, ABBOTT, Abbott Park, IL)" |
| Other bias | Low risk | None |

| Stroud 2011 [61] | | |
| --- | --- | --- |
| Risk of bias | Judgement | Support for judgement |
| Selection bias | Low risk | "Participants were recruited through printed advertisements, health fairs, direct mailings, and personal contacts (...) All participants were physically healthy with no current or personal history of psychiatric disorder with low familial risk for depression (...) Participants were carefully screened controls pooled from all three phases of the Pittsburgh Pediatric Neurobehaviroal studies." |
| Performance bias | High risk | Procedures are clearly described. Compliance to protocol is not described, but due to clinical setting of the study, protocol compliance can assumed to be good. However baseline samples were not collected in the morning, 4pm. |
| Detection bias | Low risk | "Cortisol levels were determined from 25 μL samples assayed in duplicate by solid phase ^125^I radioimmunoassay (Diagnostic Products Corporation; Los Angeles, CA)" |
| Other bias | Low risk | None |

| Stupnicki 1995 [62] | | |
| --- | --- | --- |
| Risk of bias | Judgement | Support for judgement |
| Selection bias | High risk | Not described how subjects were recruited. No baseline characteristics known between groups. Age between girls and boys in junior rowers is different (1 year difference on average). Moreover, these are athletes (level unknown), so possibly not comparable to normal adolescent population. |
| Performance bias | High risk | Well described protocol. However: “When undertaking the study, no sport-specific effects were assumed to play a role in the hormonal-exercise relationships. Therefore, both male and female athletes, of different training experience were studied as no uniform, comparable exercise protocol was considered necessary. The only condition was that the exercise was to be maximal or nearly maximal and of medium duration (12-20 min). Thus, the different types of exercise resulted from sport-specific protocols established for routine examinations of athletes.” But, all subjects performed to the maximum of their ability. |
| Detection bias | Low risk | “Cortisol concentration in serum was measured using a specific, direct (non-extraction) radio-immunoassay as described elsewhere (Stupnicki 1985). The amount of serum per assay tube was 1 p.1. The mean within-assay error in the concentration range encountered was 8.5, and between-assay 12%, respectively.” |
| Other bias | Low risk | None |

| Susman 1991 [63] | | |
| --- | --- | --- |
| Risk of bias | **Judgement** | **Support for judgement** |
| Selection bias | Unclear risk | Little is descrbed: "the adolescents were recruited into the study until there was apporximately an equal number of boys and girls in each of the five stages of pubertal development:" |
| Performance bias | Low risk | Procedures are clearly described. Compliance to protocol is not described, but due to clinical setting of the study, protocol compliance can assumed to be good |
| Detection bias | Low risk | Plasma cortisol was measured by RIA, as described in Kao et al. 1975 [58] |
| Other bias | Low risk | None |

| Syme 2008 [64] | | |
| --- | --- | --- |
| Risk of bias | Judgement | Support for judgement |
| Selection bias | High risk | Case control study investigating the long-term consequences of prenatal exposure to maternal cigarette smoking : "The following selection criteria are used for the exposed subjects: (1) Age 12–18 years; (2) One or more siblings in the same age group; (3) Maternal and paternal grand- parents of French–Canadian ancestry; and (4) Positive history of maternal cigarette smoking (>1 cigarette/day in the 2nd trimester of pregnancy) (...) The nonexposed subjects are matched to the exposed ones based on the level of maternal education and the school attended. “ [65] Moreover, baseline characteristics (i.e. height, tanner stages and fat percentage) seems to differ between boys and girls. |
| Performance bias | Unclear risk | Uniform approach: ”hospital visits always take place on Saturdays (…) a fasting blood sample is drawn between 8 am and 9 am” [65] However, "63 subjects were excluded because of technical issues". The influence of these exclusions are not further analyzed |
| Detection bias | Unclear risk | not described in either the current article as well as their study design reference [65] |
| Other bias | Low risk | None |

| Tennes 1973 [66] | | |
| --- | --- | --- |
| Risk of bias | Judgement | Support for judgement |
| Selection bias | Unclear risk | No inclusion and/or exclusion criteria or health characteristics of the subjects described |
| Performance bias | Low risk | “Blood samples were obtained from the infants on about the third day of life between 7 to 8 am” |
| Detection bias | High risk | “Plasma cortisol levels were determined in duplicate utilizing our modifications of the ultramicro protein binding method of Murphy. By this method cortisol and cortisone are not differentiated. In the newborn both cortisol and cortisone are increased following the administration of ACTH. In the present study it was assumed to be unnecessary to distinguish between the individual steroids because both cortisol and cortisone have an excitatory effect on the central nervous system” |
| Other bias | High risk | Cortisol levels are non-parametrically distributed |
| Törnhage 2002 [67] | | |
| Risk of bias | Judgement | Support for judgement |
| Selection bias | Unclear risk | The only thing that is described is "403 school-aged children were invited to participate in the study. Nine girls and five boys declined to participate. Three samples were excluded because of inconclusive assay results" |
| Performance bias | Low risk | "Saliva collection was performed in the classroom between 08.00 and 09.00h after a period of rest." |
| Detection bias | Low risk | "a commercial ^125^I RIA kit (Cortisol (^125^I) Radioimmunoassay Kit, Orion Diagnostica)" |
| Other bias | High risk | Cortisol levels are non-parametrically distributed. Moreover, calculating a SD from a range is not a robust method. |

| Tout 1998 [68] | | |
| --- | --- | --- |
| Risk of bias | Judgement | Support for judgement |
| Selection bias | Low risk | “Ninety-two children (75% of those contacted) were given permission by their parents or caregivers to participate (...) Preschool children were recruited from six classes at two urban child care centers (...) these two centers represented (...) an ethnic mix of children that was broadly representative of the metropolitan area” |
| Performance bias | Low risk | **“**The two centers also followed roughly the same daily routine and shared a similar philosophy of child care (...) Samples from individual children were assayed only if they had provided at least 10 morning and afternoon samples (morning M = 16 samples; afternoon M = 15 samples) (...) Seventeen children did not provide an adequate number of samples due to frequent absences, intermittent attendance, or refusal to participate in the sampling procedures (…) To determine whether the 17 children with missing cortisol data differed significantly from the other children, we computed a multivariate analysis of covariance (MANCOVA) entering the four observational factor measures and the teacher report measures and covarying age. The multivariate test was not significant, F(7,81) = 1.53.” |
| Detection bias | Low risk | “A modification of the Pantex RIA cortisol kit (inter- and intra-assay coefficients of variation less than 12%). (...) Samples with cortisol levels over 1.5 pg/dL were diluted to determine whether the high levels might reflect the activity of an interfering sub-stance. Only those that diluted linearly were retained. Only a few samples (less than 2%) were noted to have poor duplicates or nonlinear dilutions.“ |
| Other bias | Low risk | None |

| Tsvetkova 1977 [69] | | |
| --- | --- | --- |
| Risk of bias | Judgement | Support for judgement |
| Selection bias | Unclear risk | Children were recruited as healthy controls for several larger trials. Baseline characteristics are not described |
| Performance bias | Low risk | “At 8 am, before breakfast, 5 mL of blood was withdrawn (…) The children were then given 0.5 mg tetracosactrin intramuscularly, and another 5 mL blood sample was taken 2 hours later.” |
| Detection bias | Low risk | “The fluorescence of the sample is determined with an exciting wave length of 470mµ. A K2-8 filter is used to eliminate the scattered light. Standards containing 0.1-0.5 µg corticosterone are carried through the same procedure and the instrument is set at an arbitrary point (about 80) with the highest standard before each set of 3 samples. The fluorescence is proportional to the amount of standard in this range, after correction for the low reading obtained with the blank carried through the same procedure.” [70] |
| Other bias | Low risk | None |

| Turan 2015 [71] | | |
| --- | --- | --- |
| Risk of bias | Judgement | Support for judgement |
| Selection bias | Low risk | A population based sample with clear exclusion criteria. |
| Performance bias | High risk | Not a morning sample of cortisol. |
| Detection bias | Unclear risk | “Samples were immediately placed in a −20◦C freezer to be stored until they were shipped on dry ice to the Technical University of Dresden (Director: Clemens Kirschbaum) for hormone assays.” |
| Other bias | High risk | Cortisol levels are non-parametric distributed |

| Tzortzi 2009 [72] |  |  |
| --- | --- | --- |
| Risk of bias | Judgement | Support for judgement |
| Selection bias | Low risk | **“**School children of both genders between 10 and 14 years of age were recruited from local schools, direct communication with colleagues and their acquaintances, and a local dental office(…) The research outline and the protocol leaflets were explained to more than 100 potential test persons, of whom 30 gave informed consent (...) The exclusion criteria (chronic diseases, medication intake, hormone therapy, allergies, or any invasive medical procedure) were enforced based on a personal interview” |
| Performance bias | Low risk | “The participants were instructed to keep a diary on the sampling day, which included saliva sampling times, meal times, possible medication, physical exercise like running or cycling, and any other incidents involving physical activity (…) Red coloured samples, i.e. samples containing blood, were discarded (…) 402 samples were returned by the probands (= 95.7%). In 37 samples the noted sampling times in the probands sampling diary did not coincide with the study protocol (= 365 samples, 86.9%). Additionally, 28 cortisol samples directly correlated with physical activity were removed before further analysis (Figure 2). For final analysis, 337 (= 80.2 %) samples from 21 participants remained” |
| Detection bias | Low risk | ”Saliva cortisol determination was completed using a commercial cortisol luminescence immunoassay (part no. RE62011, IBL Immuno Biological Laboratories, Hamburg, Germany)” |
| Other bias | Low risk | None |

| Vaindirlis 2000 [73] | | |
| --- | --- | --- |
| Risk of bias | Judgement | Support for judgement |
| Selection bias | Unclear risk | “Control subjects were selected for age, sex, and pubertal stage from the healthy siblings of patients with insulin-dependent diabetes mellitus who previously acted as control subjects in a study of endothelin in insulin-dependent diabetes mellitus.” Moreover, baseline characteristics are described, however, not for boys and girls separately |
| Performance bias | Low risk | Protocol clearly described, moreover “At the time of urine delivery, subjects were asked how the urine was collected; if there was any doubt about the completion of the collection, the urine was discarded.” |
| Detection bias | Unclear risk | UET1 (Nichols Institute, Wijchen, The Netherlands) and UFC (Clinical Assays) levels were determined by radioimmunoassay according to previously reported methods |
| Other bias | Low risk | None |

| West 2010 [74] |  |  |
| --- | --- | --- |
| Risk of bias | Judgement | Support for judgement |
| Selection bias | High risk | **“**Conducted among pupils within 22 schools (...) The sampling scheme aimed to obtain a representative sample by selecting schools within strata based on geographical location (within Glasgow City or not), religious status (Catholic/Non-denominational) and deprivation (…) Table 1 shows the descriptive statistics of all variables used in the analysis for each sex and the total sample. Of interest is the shorter mean time (but greater variability), between awakening and T1 among males (1.80 h; mean wake time, 0729) compared with females (1.94 h; mean wake time, 0721) (p<0.001). Females were assessed as more mature (p<0.001) though there was no gender difference in BMI categories. A majority (64%) reported eating something in the previous hour, otherwise the proportions engaging in behaviours linked to cortisol were small, and with the exception of smoking (more females, p<0.001) similar between the sexes. With respect to school hierarchies, females rated them- selves higher on the scholastic hierarchy, males higher on both peer and sports hierarchies (all p<0.001).” |
| Performance bias | Low risk | Protocol is clearly desribed. Moreover, “Extreme cases, presumed to be contaminated by blood, were excluded from analysis.” |
| Detection bias | Low risk | **“**Samples were analysed by single analysis using the Tekan Stäfa, Swit-roboting pipetting system (Genesis 150, Tekan, Stäfa, Switzerland) using the IBL luminometric assay (IBL, Hamburg, Germany)” |
| Other bias | High risk | The unit of measurement of cortisol concentrations was not described. While the same cohort was presented in (Kelly, Young, Sweeting, Fischer, & West, 2008), I presumed the unit of measurement was nmol/L in the current study as well. |

| Wudy 2007 [75] | | |
| --- | --- | --- |
| Risk of bias | Judgement | Support for judgement |
| Selection bias | Low risk | “Fifty 24-h urine samples (25 from boys and 25 from girls) were randomly selected for each of the eight equally wide age groups.”  “a selected, non-representative study sample (Table 1). Compared to the general population the parents of the DONALD Study children are characterised by a higher educational attainment and higher socio-economic status. Comparing body mass index (BMI) to the German reference data, only slight deviations from the percentile curves can be observed (Figs.2 and 3). From this point of view the selection procedure did not result in a major deviation from the reference population.”[76] |
| Performance bias | Low risk | “The participating children and their parents received instruction and written guidance to ensure compliance in the 24-h urine collection, which was performed at home by each subject (...) a dietitian visiting the families and discussing collection completeness in detail” |
| Detection bias | Low risk | “Urinary steroid profiles were determined using quantitative data produced by GC-MS analysis according” |
| Other bias | Low risk | None |

| Yu 2009 [77] | | |
| --- | --- | --- |
| Risk of bias | **Judgement** | **Support for judgement** |
| Selection bias | Unclear risk | “20 non-aggressive students were selected from 1051 students aged 11-16 years from two middle schools in Wuhan city, Hubei province of China. The average age of the aggressive and non-aggressive students was 12.8±1.6 years and 12.6±1.8 years, respectively (P<0.05). The aggressive students and non-aggressive controls were matched for gender, age, grade, Tanner stage, and family economic status.” Moreover, no baseline characteristics, as well as no inclusion or exclusion criteria are described. |
| Performance bias | High risk | “A 5.0 mL saliva sample was collected from each individual at the same time point during school day (1:30-2:30 pm). Saliva specimens were collected from all female students during their follicular phase of menstrual cycles.” No morning cortisol sample. |
| Detection bias | Low risk | “The levels of salivary CORT, T, GH, and PRL were measured by radioimmunoassay using DFM-96 type ten-tube radioimmunity and γ counting  apparatus. Radioimmunoassay kits were purchased from CHEMCLIN of Beijing.” |
| Other bias | Low risk | None |

**References of Additional File 2**

1. Alghadir A, Gabr S, Al-Eisa E. Effects of Physical Activity on Trace Elements and Depression Related Biomarkers in Children and Adolescents. Biol Trace Elem Res. 2016;172:299-306.
2. Allen LB, Lu Q, Tsao JCI, Worthman CM, Zeltzer LK. Sex differences in the association between cortisol concentrations and laboratory pain responses in healthy children. Gend Med. 2009; 6 Suppl 2:193–207.
3. Higgins J, Green S. Cochrane Handbook for Systematic Reviews of Interventions. The Cochrane Collaboration. 2011. www.cochrane-handbook.org. Version 5.1.0
4. Apter D, Pakarinen A, Hammond GL, Vihko R. Adrenocortical function in puberty. serum ACTH, cortisol and dehydroepiandrosterone in girls and boys. Acta Paediatr Scand. 1979;68:599–604.
5. Apter D, Pakarinen A, Vihko R. Serum prolactin, FSH and LH during puberty in girls and boys. Acta Paediatr Scand. 1978;67:417–23.
6. Apter D, Jänne O, Vihko R. Lipidex chromatography in the radioimmunoassay of serum and urinary cortisol. Clin Chim Acta. 1975;63:139–48.
7. Azurmendi A, Pascual-Sagastizabal E, Vergara A, Munoz J, Braza P, Carreras R, et al. Developmental Trajectories of Aggressive Behavior in Children from Ages 8 to 10: The Role of Sex and Hormones. Amnerican J Hum Biol. 2016;28:90–7.
8. Bailey D, Colantonio D, Kyriakopoulou L, Cohen AH, Chan MK, Armbruster , et al. Marked biological variance in endocrine and biochemical markers in childhood: Establishment of pediatric reference intervals using healthy community children from the CALIPER cohort. Clin Chem. 2013;59:1393–403.
9. Colantonio DA, Kyriakopoulou L, Chan MK, Daly CH, Brinc D, Venner AA, et al. Closing the gaps in pediatric laboratory reference intervals: A caliper database of 40 biochemical markers in a healthy and multiethnic population of children. Clin Chem. 2012;58:854–68.
10. Belva F, Painter RC, Schiettecatte J, Bonduelle M, Roelants M, Roseboom TJ, et al. Gender-specific alterations in salivary cortisol levels in pubertal intracytoplasmic sperm injection offspring. Horm Res Paediatr. 2013;80:350–5.
11. Belva F, Painter R, Bonduelle M, Roelants M, Devroey P, De Schepper J. Are ICSI adolescents at risk for increased adiposity? Hum Reprod. 2012;27:257–64.
12. Canalis E, Reardon GE, Caldarella AM. A more specific, liquid-chromatographic method for free cortisol in urine. Clin Chem. 1982;28:2418–20.
13. Chen FR, Raine A, Rudo-Hutt AS, Glenn AL, Soyfer L, Granger DA. Harsh discipline and behavior problems: The moderating effects of cortisol and alpha-amylase. Biol Psychol. 2014;104:19–27.
14. Liu J, Richmond T, Raine A, Cheney R, Brodkin E, Gur R, et al. The Healthy Brains and Behavior Study: objectives, design, recruitment, and population coverage. Int J Methods Psychiatr Res. 2013;22:204–16.
15. Cicchetti D, Rogosch FA. The impact of child maltreatment and psychopathology on neuroendocrine functioning. Dev Psychopathol. 2001;13:783–804.
16. Cieslak TJ, Frost G, Klentrou P. Effects of physical activity, body fat, and salivary cortisol on mucosal immunity in children. J Appl Physiol (1985). 2003;95:2315-20.
17. Colomina MT, Canals J, Carbajo G, Domingo JL. Salivary cortisol in a young population: Relationship with psychopathological disorders. Res Commun Biol Psychol Psychiatry. 1997;22:1–10.
18. Covelli MM, Wood CE, Yarandi HN. Biologic measures as epidemiological indicators of risk for the development of hypertension in an African American adolescent population. J Cardiovasc Nurs. 2012;27:476–84.
19. Daughters SB, Gorka SM, Matusiewicz A, Anderson K. Gender specific effect of psychological stress and cortisol reactivity on adolescent risk taking. J Abnorm Child Psychol. 2013;41:749–58.
20. Davis M, Emory E. Sex differences in neonatal stress reactivity. Child Dev. 1995;66:14–27.
21. De Bruijn ATCE, Van Bakel HJA, Wijnen H, Pop VJM, Van Baar AL. Prenatal maternal emotional complaints are associated with cortisol responses in toddler and preschool aged girls. Dev Psychobiol. 2009;51:553–63.
22. Dressendörfer RA, Kirschbaum C, Rohde W, Stahl F, Strasburger CJ. Synthesis of a cortisol-biotin conjugate and evaluation as a tracer in an immunoassay for salivary cortisol measurement. J Steroid Biochem Mol Biol. 1992;43:683–92.
23. Dietrich A, Ormel J, Buitelaar JK, Verhulst FC, Hoekstra PJ, Hartman CA. Cortisol in the morning and dimensions of anxiety, depression, and aggression in children from a general population and clinic-referred cohort: An integrated analysis. The TRAILS study. Psychoneuroendocrino. 2013;38:1281–98.
24. Huisman M, Oldehinkel AJ, De Winter A, Minderaa RB, De Bildt A, Huizink AC, et al. Cohort profile: The Dutch “Tracking Adolescents” individual lives’ survey'; TRAILS. Int J Epidemiol. 2008;37:1227–35.
25. Rosmalen JGM, Oldehinkel AJ, Ormel J, de Winter AF, Buitelaar JK, Verhulst FC. Determinants of salivary cortisol levels in 10-12 year old children; a population-based study of individual differences. Psychoneuroendocrino. 2005;30:483–95.
26. Elmlinger MW, Kühnel W, Ranke MB. Reference ranges for serum concentrations of lutropin (LH), follitropin (FSH), estradiol (E2), prolactin, progesterone, sex hormone-binding globulin (SHBG), dehydroepiandrosterone sulfate (DHEAS), cortisol and ferritin in neonates, children and young adul. Clin Chem Lab Med. 2002;40:1151–60.
27. Forest MG. Age-related response of plasma testosterone, delta 4-androstenedione, and cortisol to adrenocorticotropin in infants, children, and adults. J Clin Endocrinol Metab. 1978;47:931–7.
28. Fransson E, Folkesson L, Bergström M, Östberg V, Lindfors P. Exploring salivary cortisol and recurrent pain in mid-adolescents living in two homes. BMC Psychol. 2014;2:1–7.
29. Garagorri JM, Rodríguez G, Lario-Elboj ÁJ, Olivares JL, Lario-Muñoz Á, Orden I. Reference levels for 17-hydroxyprogesterone, 11-desoxycortisol, cortisol, testosterone, dehydroepiandrosterone sulfate and androstenedione in infants from birth to six months of age. Eur J Pediatr 2008;167:647–53.
30. Georgopoulos NA, Rottstein L, Tsekouras A, Theodoropoulou A, Koukkou E, Mylonas P, et al. Abolished circadian rhythm of salivary cortisol in elite artistic gymnasts. Steroids. 2011;76: 353–7.
31. Ghaziuddin N, Welch K, Greden J. Central serotonergic effects of m-chlorophenylpiperazine (mCPP) among normal control adolescents. Neuropsychopharmacol. 2003;28:133–9.
32. Gunnar MR, Kryzer E, Van Ryzin MJ, Phillips DA. The Rise in cortisol in family day care: Associations with aspects of care quality, child behavior, and child sex. Child Dev. 2010;81:851–69.
33. Hackney AC, McMurray RG, Judelson DA, Harrell JS. Relationship between caloric intake, body composition, and physical activity to leptin, thyroid hormones, and cortisol in adolescents. Jpn J Physiol. 2003;53:475–9.
34. Harrell JS, McMurray RG, Bangdiwala SI, Frauman AC, Gansky SA, Bradley CB. Effects of a school-based intervention to reduce cardiovascular disease risk factors in elementary-school children: the Cardiovascular Health in Children (CHIC) study. J Pediatr. 1996;128:797–805.
35. Honour JW, Jones R, Leary S, Golding J, Ong KK, Dunger DB. Relationships of urinary adrenal steroids at age 8 years with birth weight, postnatal growth, blood pressure, and glucose metabolism. J Clin Endocrinol Metab. 2007;92:340–5.
36. Huybrechts I, De Vriendt T, Breidenassel C, Rogiers J, Vanaelst B, Cuenca-García M, et al.; HELENA Study Group. Mechanisms of stress, energy homeostasis and insulin resistance in European adolescents - the HELENA study. Nutr Metab Cardiovasc Dis. 2014;24:1082–9.
37. Béghin L, Castera M, Manios Y, Gilbert CC, Kersting M, De Henauw S, et al.; HELENA Study Group. Quality assurance of ethical issues and regulatory aspects relating to good clinical practices in the HELENA Cross-Sectional Study. Int J Obes. 2008;32:S12–8.
38. Ilias I, Ghizzoni L, Mastorakos G. Orderliness of cortisol, growth hormone, and leptin secretion in short-normal pre-pubertal boys and girls. Med Sci Monit. 2009;15:242–7.
39. Jones A, Godfrey KM, Wood P, Osmond C, Goulden P, Phillips DIW. Fetal growth and the adrenocortical response to psychological stress. J Clin Endocrinol Metab. 2006;91:1868–71.
40. Godfrey K, Walker-Bone K, Robinson S, Taylor P, Shore S, Wheeler T, et al. Neonatal bone mass: influence of parental birthweight, maternal smoking, body composition, and activity during pregnancy. J bone Miner Res. 2001;16:1694–703.
41. Lashansky G, Saenger P, Fishman K, Gautier T, Mayes D, Berg G, et al. Normative data for adrenal steroidogenesis in a healthy pediatric population: Age- and sex-related changes after adrenocorticotropin stimulation. J Clin Endocrinol Metab. 1991;73:674–86.
42. Lundberg U, De Chateau P, Winberg J, Frankenhaeuser M. Catecholamine and cortisol excretion patterns in three-year-old children and their parents. J Human Stress. 1981;7:3–11.
43. Ficher M, Curtis GC, Ganjam VK, Joshlin L, Perry S. Improved Measurement of Corticosteroids in Plasma and Urine by Competitive Protein-Binding Radioassay. Clin Chem. 1973;19:511–5.
44. Lundberg U. Sex differences in behaviour pattern and catecholamine and cortisol excretion in 3-6 year old day-care children. Biol Psychol. 1983;16:109–117.
45. Martikainen S, Pesonen A-K, Lahti J, Heinonen K, Feldt K, Pyhälä R, et al. Higher levels of physical activity are associated with lower hypothalamic-pituitary-adrenocortical axis reactivity to psychosocial stress in children. J Clin Endocrinol Metab. 2013;98:E619–27.
46. Michels N, Sioen I, Huybrechts I, Bammann K, Vanaelst B, De Vriendt T, et al. Negative life events, emotions and psychological difficulties as determinants of salivary cortisol in Belgian primary school children. Psychoneuroendocrino. 2012;37:1506–15.
47. Michels N, Vanaelst B, Vyncke K, Sioen I, Huybrechts I, De Vriendt T, et al. Children’s Body composition and Stress - the ChiBS study: aims, design, methods, population and participation characteristics. Arch Public Heal. 2012;70:17.
48. Mills RS, Imm GP, Walling BR, Weiler HA. Cortisol reactivity and regulation associated with shame responding in early childhood. Dev Psychol. 2008;44:1369–80.
49. Minkley N, Kirchner WH. Influence of test tasks with different cognitive demands on salivary cortisol concentrations in school students. Int J Psychophysiol. 2012;86:245–50.
50. Mrug S, Tyson A, Turan B, Granger DA. Sleep problems predict cortisol reactivity to stress in urban adolescents. Physiol Behav. 2016;155:95–101.
51. Nakamura J, Yakata M. Age- and sex-related differences in urinary cortisol level. Clin Chim Acta. 1984;137:77–80.
52. Ong KK, Potau N, Petry CJ, Jones R, Ness AR, Honour JW, et al.; Avon Longitudinal Study of Parents and Children Study Team. Opposing influences of prenatal and postnatal weight gain on adrenarche in normal boys and girls. J Clin Endocrinol Metab. 2004;89:2647–51.
53. Osika W, Friberg P, Wahrborg P. A new short self-rating questionnaire to assess stress in children. Int J Behav Med. 2007;14:108–17.
54. Pérez-Edgar K, Schmidt LA, Henderson HA, Schulkin J, Fox NA. Salivary cortisol levels and infant temperament shape developmental trajectories in boys at risk for behavioral maladjustment. Psychoneuroendocrino. 2008;33:916–25.
55. Kagan J, Snidman N. Infant Predictors of Inhibited and Uninhibited Profiles. Psychol Sci. 1991;2:40–4.
56. Portnoy J, Raine A, Glenn AL, Chen FR, Choy O, Granger DA. Digit ratio (2D:4D) moderates the relationship between cortisol reactivity and self-reported externalizing behavior in young adolescent males. Biol Psychol. 2015;112:94–106.
57. Reynolds RM, Hii HL, Pennell CE, McKeague IW, Kloet ER de, Lye S, et al. Analysis of baseline hypothalamic-pituitary-adrenal activity in late adolescence reveals gender specific sensitivity of the stress axis. Psychoneuroendocrino. 2013;38:1271–80.
58. Ross JL, Schulte HM, Gallucci WT, Cutler GB, Loriaux DL, Chrousos GP. Ovine corticotropin-releasing hormone stimulation test in normal children. J Clin Endocrinol Metab. 1986;62:390–2.
59. Kao M, Voina S, Nichols A, Horton R. Parallel Radioimmunoassay for Plasma Cortisol and 11-Deoxycortisol. Clin Chem. 1975;21:1644–7.
60. Soriano-Rodriguez P, Osiniri I, Grau-cabrera P, Riera-perez E, Prats-puig A, Carbonell-Alferez M, et al. Physiological Concentrations of Serum Cortisol Are Related to Vascular Risk Markers in Prepubertal Children. Pediatr Res. 2010;68:452–5.
61. Stroud LR, Papandonatos GD, Williamson DE, Dahl RE. Sex differences in cortisol response to corticotropin releasing hormone challenge over puberty: Pittsburgh Pediatric Neurobehavioral Studies. Psychoneuroendocrino. 2011;36:1226–38.
62. Stupnicki R, Obmiński Z, Klusiewicz A, Viru A. Pre-exercise serum cortisol concentration and responses to laboratory exercise. Eur J Appl Physiol Occup Physiol. 1995;71:439–43.
63. Susman EJ, Dorn LD, Chrousos GP. Negative affect and hormone levels in young adolescents: Concurrent and predictive perspectives. J Youth Adolesc. 1991;20:167–90.
64. Syme C, Abrahamowicz M, Leonard GT, Perron M, Pitiot A, Qiu X, et al. Intra-abdominal adiposity and individual components of the metabolic syndrome in adolescence: sex differences and underlying mechanisms. Arch Pediatr Adolesc Med. 2008;162:453–61.
65. Pausova Z, Paus T, Abrahamowicz M, Almerigi J, Arbour N, Bernard M, et al. Genes, maternal smoking, and the offspring brain and body during adolescence: Design of the Saguenay Youth Study. Hum Brain Mapp. 2007;28:502–18.
66. Tennes K, Carter D. Plasma Cortisol Levels and Behavioral States in Early Infancy The purpose of this study was to examine the relationship between plasma cortisol levels and behavioral states in the infant at birth and during the first three months of life . Convincing evid. 1973;35:121–8.
67. Tornhage C. Reference values for morning salivary cortisol concentrations in healthy school-aged children. J Pediatr Endocrinol Metab. 2002;15:197–204.
68. Tout K, de Haan M, Campbell EK, Gunnar MR. Social Behavior Correlates of Cortisol Activity in Child Care: Gender Differences and Time-Of-Day Effects. Child Dev. 1998;69:1247–62.
69. Tsvetkova V. Adrenocortical function after stimulation with synthetic ACTH. Curr Med Res Opin. 1977;4:635–9.
70. Silber RH, Busch RD, Oslapas R. Practical procedure for estimation of corticosterone or hydrocortisone. Clin Chem. 1958;4:278–85.
71. Turan B, Tackett JL, Lechtreck MT, Browning WR. Coordination of the cortisol and testosterone responses: A dual axis approach to understanding the response to social status threats. Psychoneuroendocrino. 2015;62:59–68.
72. Tzortzi C, Proff P, Redlich M, Aframian DJ, Palmon A, Golan I, et al. Cortisol daily rhythm in saliva of healthy school children. Int Dent J. 2009;59:12–8.
73. Vaindirlis I, Peppa-Patrikiou M, Dracopoulou M, Manoli I, Voutetakis A, Dacou-Voutetakis C. “White coat hypertension” in adolescents: increased values of urinary cortisol and endothelin. J Pediatr. 2000;136:359–64.
74. West P, Sweeting H, Young R, Kelly S. The relative importance of family socioeconomic status and school-based peer hierarchies for morning cortisol in youth: An exporatory study. Soc Sci Med. 2010;70:1246–53.
75. Wudy SA, Hartmann MF, Remer T. Sexual dimorphism in cortisol secretion starts after age 10 in healthy children: urinary cortisol metabolite excretion rates during growth. Am J Physiol Endocrinol Metab. 2007;293:E970–6.
76. Kroke A, Manz F, Kersting M, Remer T, Sichert-Hellert W, Alexy , et al. The DONALD Study History, current status and future perspectives. EurJNutr. 2004;43:45–54.
77. Yu YZ, Shi JX. Relationship between levels of testosterone and cortisol in saliva and aggressive behaviors of adolescents. Biomed Environ Sci. 2009;22:44–9.
