# Supplementary material for: Gender-specific differences in hypothalamus–pituitary–adrenal axis activity during childhood: a systematic review and meta-analysis
Source: Biol Sex Differ. 2017 Jan 19;8:3. doi: 10.1186/s13293-016-0123-5 (PMC5244584; doi:10.1186/s13293-016-0123-5)
Supplement: Additional file 3: — Extracted data of studies included in the meta-analysis (DOCX 2550 kb) [file 13293_2016_123_MOESM3_ESM.docx]

**Extracted data of studies included in the meta-analysis**

| **Alghadir 2015** [1**]** | |
| --- | --- |
| **Methods** | **Design** Prospective, observational study |
|  | **Setting** Mansoura, Egypt |
|  | **Timing**  9:00 – 11:30 am |
| **Participants** | ***n=*** 150 |
|  | **Subjects**  Randomly selected at elementary and secondary public schools |
|  | **Age** G1 boys 9.3 ± 1.5yr; G2 boys 14.9 ± 3.7yr; G1 girls 8.96 ± 1.8yr;  G2 girls 14.82 ± 4.6yr |
|  | **Sex** 90 boys and 60 girls |
|  | **Exclusion criteria** “None of the school children included in this study reported any kind of disability such as cerebral palsy, muscle weakness, paralysis, or mental retardation” |
| **Protocol** | Mid-morning (9:00 – 11:30 am) saliva samples were collected from all participants following overnight fast |
| **Outcomes** | **Primary** “to investigate the effect of physical activity (PA) on depressive symptoms in children and adolescents.” |
|  | **Secondary** “ to study the association of demographic variables, serum levels of Copper (Cu), Zinc (Zn), serotonin, and salivary cortisol with depression in this population.” |
| **Results** | \|  \| G1 (7-11yr) \| \| \| \| G2 (12-18yr; *n*=85) \| \| \| \| \| --- \| --- \| --- \| --- \| --- \| --- \| --- \| --- \| --- \| \|  \| Sendentary \| \| Active \| \| Sendentary \| \| Active \| \| \|  \| Boys \| Girls \| Boys \| Girls \| Boys \| Girls \| Boys \| Girls \| \| *n* \| 12 \| 16 \| 25 \| 12 \| 20 \| 30 \| 25 \| 10 \| \| Cortisol (pg/ml) \| 65.4  ± 5.2 \| 71.5  ± 10.3 \| 48.6  ± 9.5 \| 56.8  ± 6.3 \| 78.5  ± 5.4 \| 86.9  ± 12.3 \| 56.7  ± 6.4 \| 61.2  ± 5.2 \|   For the meta-analysis all the sample groups (G1 and G2; sendentary and active) were included.  I emailed Dr. Gabr ('sgabr@ksu.edu.sa'; 'nadalab2009@hotmail.com'; 'drGabr14@yahoo.com') to check the unit of cortisol, since pg/mL is not often used and these salivary cortisol levels are very low. He did not respond, so we assumed that it was pg/mL |

| **Allen 2009** [2] | |
| --- | --- |
| **Methods** | **Design** non-randomized intervention study |
|  | **Setting** California, USA |
|  | **Timing** not described |
| **Participants** | ***n=*** 235 |
|  | **Subjects** “children who, by parent and self-report, had no acute or chronic illness such as a heart condition or arthritis, recent surgery, injury to any limb, history of frostbite, history of fainting spells, or developmental delay. “ |
|  | **Age** 12.7 ± 2.9 yr |
|  | **Sex** 119 males and 116 females |
|  | **Exclusion criteria**“(1) acute or chronic illness at the time of study participation; (2) developmental delay or significant anatomic impairment that would preclude understanding of the study procedures or participation in pain-induction procedures (3) daily use of opioid medication.” |
| **Protocol** | “Salivary cortisol samples (…) were obtained at baseline after entering the laboratory (SCb), after the completion of all pain tasks (SC1), and at the end of the session (SC2), 20 minutes later (…)Participants were then exposed to 3 pain tasks— pressure, heat, and cold pressor—with the order of tasks counterbalanced across participants.“ |
| **Outcomes** | **Primary** to examine cortisol–pain response relationships across 2 methods of assessment (salivary and blood cortisol levels). |
|  | **Secondary** potential sex differences in patterns of cortisol reactivity. |
| **Results** | \| Gender \|  \| Baseline salivary cortisol,  nmol/L \| \| --- \| --- \| --- \| \| *Males* \| *n* \| 119 \| \|  \| Mean(SD) \| 0.17 (0.18) \| \| Females \| *n* \| 116 \| \|  \| Mean(SD) \| 0.13 (0.09) \| \| Total \| *n* \| 235 \| \|  \| Mean(SD) \| 0.15 (0.14) \| |
|  | |

| Apter 1979 [3] | |
| --- | --- |
| **Methods** | **Design** longitudinal cohort study |
|  | **Setting** Helsinki, Finland |
|  | **Timing** 08 – 10am |
| **Participants** | ***n=*** 280 |
|  | **Subjects** healthy [4]; not further described. |
|  | **Age** 7.5 – 18.5 yr |
|  | **Sex** 200 girls and 80 boys |
|  | **Exclusion criteria** not described |
| **Protocol** | Morning serum samples |
| **Outcomes** | **Primary** gender- and age-specific serum ACTH, cortisol and DHEA |
|  | **Secondary** serum ACTH, cortisol and DHEA according to pubertal stages |
| **Results** | “In girls bone age determination was carried out at each examination and therefore the data on girls are given in relation to bone age. In boys, bone age was not regularly recorded and the data are therefore given in relation to chronological age.”   \| Serum cortisol in girls according to bone age \| \| \| \| --- \| --- \| --- \| \|  \|  \| Age (yr) \| \|  \|  \| 7.5 \| \| *n* \|  \| 7 \| \| Cortisol, µg/L \| Mean \| 82 \| \|  \| S.E.M. \| 15 \|  \| Serum cortisol in boys according to chronological age \| \| \| \| --- \| --- \| --- \| \|  \|  \| Age (yr) \| \|  \|  \| 8.5 \| \| *n* \|  \| 7 \| \| Cortisol, µg/L \| Mean \| 87 \| \|  \| S.E.M. \| 11 \|   “Samples are analyzed in a mixed longitudinal approach (2 – 3 samples each person). Table 1 and 2 give the mean concentration of all samples analyzed cross-sectionally per age groups.”  Therefore, we will report only the concentration for the 7.5 – 8.5yr age group.  Standard errors were converted to SDs: SD= SE x √N [5] |

| **Azurmendi 2016** [6**]** | |
| --- | --- |
| **Methods** | **Design** longitudinal study |
|  | **Setting** San Sebastian, Spain |
|  | **Timing** 9:00 am |
| **Participants** | ***n=*** 90 |
|  | **Subjects** “The socioeconomic status of the participants in the sample was considered to be medium and medium-high, based on our knowledge of the area in which participants live.” |
|  | **Age** 8 year and 10 year; not further described |
|  | **Sex** 49 boys and 41 girls |
|  | **Exclusion criteria** not described |
| **Protocol** | “Saliva samples were taken by passive drool into a plastic cup (sterile container for the hygienic collection of biological samples) (…) Saliva samples (…) were taken on two different occasions in each year of the study (all at the same time, 09:00 h, with an interval of 6 weeks, to control diurnal patterns)” |
| **Outcomes** | **Primary** “to explore whether, in a transitional stage toward puberty, changes occur in the developmental trajectory of aggressive behavior in a sample of  children from age 8 to age 10.” |
|  | **Secondary** “whether or not they are predicted by sex and/or changes in hormone levels between these two ages” |
| **Results** | For the meta-analysis the 8 year sample was used.  The unit of measurement of cortisol was not given. Dr. Azurmendi was e-mailed to confirm if it was μg/dL. He did not respond, so we assumed that it was μg/dL and converted the data to nmol/L.   \|  \| \| Boys \| Girls \| \| --- \| --- \| --- \| --- \| \|  \| \| 8-yr-old \| 8-yr-old \| \| Cortisol \| Mean (SD) \| 0.29 (0.15) \| 0.23 (0.13) \| \|  \| range \| 0.1 – 0.7 \| 0.1 – 0.7 \| |

| Bailey 2013 [7] | | |
| --- | --- | --- |
| **Methods** | | **Design** Cross-sectional study |
|  |  | **Setting** Toronto, Canada |
|  |  | **Timing** 9:00 am – 10 pm |
| **Participants** | | ***n=*** 1482 |
|  |  | **Subjects** Samples from participants older than 1 year were collected from healthy children in the community. Samples from participants younger than 1 year were collected from leftover samples from apparently healthy/metabolically stable children to ensure a sufficiently large sample size. These samples were obtained in outpatient clinics, which included dentistry, fracture, and plastic surgery (93% outpatients; 7% from community children).” [8] Samples from participants younger than 14 days old were obtained from neonates in the maternity ward who had been deemed healthy and were being sent home.” |
|  |  | **Age** 0 – 18 years |
|  |  | **Sex** 736 boys and 746 girls |
|  |  | **Exclusion criteria** “a history of chronic illness or metabolic disease, an acute illness within the previous month, or use of prescribed medication over the previous 2 weeks. “[8] |
| **Protocol** | | Whole-blood samples were collected for establishing age- and sex-specific reference intervals for 14 immunochemical analytes measured on the Abbott ARCHITECT i2000 instrument system including: alfa-fetoprotein (AFP), cobalamin (vitamin B12), folate, total homocysteine (tHcy), ferritin, cortisol, troponin I (TnI), 25(OH)-vitamin D [25(OH)D], intact parathyroid hormone (iPTH), thyroid- stimulatinghormone (TSH), total thyroxine (TT4), total triiodothyronine (TT3), free T4 (FT4), and free T3 (FT3). |
| **Outcomes** | | **Primary** age- and sex-specific reference intervals for 40 serum biochemical markers. |
|  |  | **Secondary** the influence of ethnicity in children older than 1 year. |
| **Results** | | I edited the raw data from the supplementary file:   \| Age group \| Gender=1 \| \| \| Gender=2 \| \| \| \| --- \| --- \| --- \| --- \| --- \| --- \| --- \| \|  \| Mean \| Sds \| N \| Mean \| Sds \| N \| \| 0-2 \| 159.8349 \| 135.3292 \| 261 \| 190.1688 \| 186.2016 \| 208 \| \| 2-8 \| 134.4109 \| 75.57116 \| 110 \| 129.9081 \| 71.47614 \| 86 \| \| 8-18 \| 218,3151 \| 98,93106 \| 370 \| 215,7187 \| 110,7262 \| 369 \| \|  \|  \|  \|  \|  \|  \|  \|     Note. In the supplementary file it is not described which gender are the males or females (1 or 2). I have emailed the research group [ManKhun.Chan@sickkids.ca] and they answered: 1 = Male 2 = Female |
| **Belva 2013** [9] | | |
| **Methods** | **Design** Prospective, observational study | |
|  | **Setting** Brussels, Belgium | |
|  | **Timing** on a weekday between 06: 00 and 07: 00 am | |
| **Participants** | ***n=*** 223 | |
|  | **Subjects** children born by intracytoplasmic sperm injection (ICSI) and children born after spontaneous conception (SC). | |
|  | **Age** ± 14.0 yr | |
|  | **Sex** SC children: 115 males, 108 females | |
|  | **Exclusion criteria** for SC children: no use of hormonal stimulation [10] | |
| **Protocol** | **Protocol** salivary cortisol was measured | |
| **Outcomes** | **Primary “**to investigate if an end-product of HPA axis functioning is altered among children born after assisted conception.” | |
|  | **Secondary** NA | |
| **Results** | In males, mean salivary cortisol levels in the SC group (8.9 µg/l; 95% CI 8.3–9.7)  In females, mean salivary cortisol levels in the SC group(10.6 µg/l; 95% CI 9.7–11.5)  We converted 95%CIs to SEs by calculating: upper limit – lower limit /3.92 [5] and subsequently converted the SEs to SDs.  Males: 24.6 ± 10.57 nmol/L Females: 29.2 ± 13.2 nmol/L | |

| Canalis, 1982 [11] | |
| --- | --- |
| **Methods** | **Design** Prospective, observational study |
|  | **Setting** Connecticut, USA |
|  | **Timing** 24h-urine |
| **Participants** | ***n=*** 29 |
|  | **Subjects** “None of these persons was taking any medications known to affect the pituitary-adrenal axis” |
|  | **Age** 4 – 15 yr |
|  | **Sex** 14 boys, 15 girls |
|  | **Exclusion criteria** Not specified |
| **Protocol** | Collection of 24h urine |
| **Outcomes** | **Primary** Comparing new LC method to RIA |
|  | **Secondary** Determining specificity and sensitivity of the LC method, as well as analytical recovery, precision and accuracy and stability. |
| **Results** | \| Urinary Cortisol in Subjects with normal adrenal function \| \| \| \| \| \| --- \| --- \| --- \| --- \| --- \| \|  \| *n* \| µg/24h \| µg/g creatinine \| µg/m^2^ \| \| Children \| 29 \| 14.1 ± 6.0 \| 27.3 ± 14.0 \| 14.4 ± 7.4 \| \| Boys \| 14 \| 15.2 ± 6.3 \| 26.2 ± 14.0 \| 15.3 ± 7.3 \| \| Girls \| 15 \| 13.0 ± 5.6 \| 28.3 ± 10.5 \| 13.5 ± 7.7 \| |

| Chen 2014 [12] | |
| --- | --- |
| **Methods** | **Design** Prospective, observational study |
|  | **Setting** Pennsylvania, USA |
|  | **Timing** The first sample was collected at approximately 9 am |
| **Participants** | ***n=*** 425 |
|  | **Subjects** “Community-residing 11 and 12 year-old boys and girls living in Philadelphia County or Pennsylvania” [13] |
|  | **Age** 11.87 ± 0.60 yr |
|  | **Sex** 213 males and 212 females |
|  | **Exclusion criteria** “1.Psychotic disorder 2. Mental retardation 3. Claustrophobia 4. Pervasive developmental disorders 5. Conditions that precludes participation (or increase risk) in the clinical trial (Type 1 diabetes mellitus; metabolic diseases, gastrointestinal disorders affecting nutrient absorption, cancer) 6. Currently on medication that may modify lipid metabolism 7. Extensive use of nutritional supplements within the previous three months 8. Seafood allergy 9. Presence or history of orthopedic circumstances and metallic inserts interfering with magnetic resonance scanning 10. Pregnancy.”[13] |
| **Protocol** | Across a single day, three saliva samples were collected: the first at 9 am, the second 15 min later, and the third 30 min later. |
| **Outcomes** | **Primary** To test the individual and interactive effect of harsh discipline, cortisol and salivary alpha-amylase levels on behavior problems |
|  | **Secondary** High vs. low latent levels. |
| **Results** | Sample 1 was included in the meta-analysis   \|  \|  \|  \| Boys  (*n* ***=*** 213) \| Girls  (*n* ***=*** 212) \| \| --- \| --- \| --- \| --- \| --- \| \| Cortisol (µg/dL) \| Means (SD) \| Sample 1 \| 0.17 (0.20) \| 0.20 (0.19) \| \| Saliva collection time (h) \|  \| Sample 1 \| 9.31 (0.32) \| 9.29 (0.37) \| |

| Cicchetti 2001 [14] | | |  |
| --- | --- | --- | --- |
| **Methods** | | **Design** Case-control study |  |
|  |  | **Setting** New York, USA |  |
|  |  | **Timing** at 9am and at 4pm |  |
| **Participants** | | **N** 204 (non-maltreated) |  |
|  |  | **Subjects “**Maltreated and non-maltreated low-income disadvantaged children” |  |
|  |  | **Age** 9.24 ± 2.33 yr |  |
|  |  | **Sex** 225 boys 146 girls (= total group) |  |
|  |  | **Exclusion criteria** for non-maltreatment group: if they had any previous contact with social services or services to prevent foster care placement. |  |
| **Protocol** | | “Saliva samples were obtained twice daily from the children at the same time in the morning (i.e., 9:00 am) and in the afternoon (i.e., 4:00 pm)” |  |
| **Outcomes** | | **Primary** clinical level of internalizing and externalizing problems in maltreated and non-maltreated children |  |
|  |  | **Secondary** (1) basal levels and decrease over the day of cortisol in maltreated and non-maltreated children with or without clinical levels of internalizing or externalizing problems. (2) The role of gender as a possible moderator. |  |
| **Results** | | 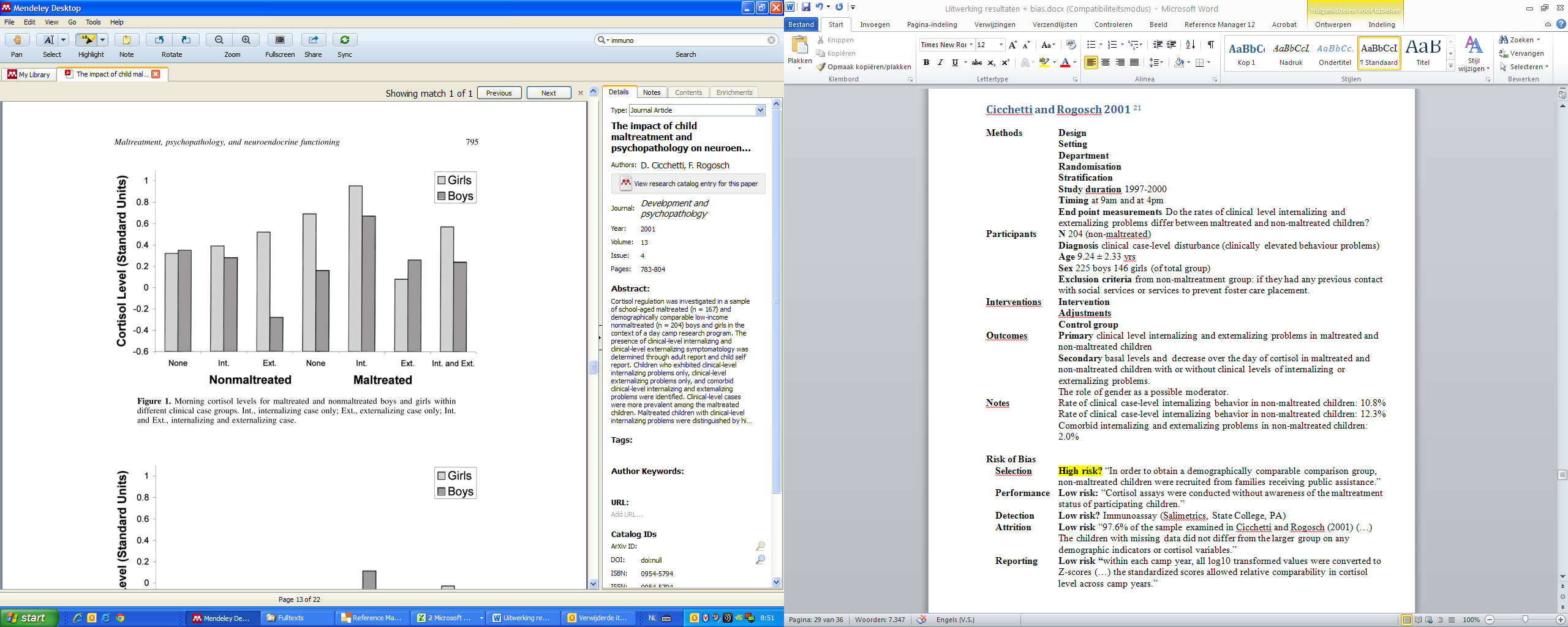  “A significant within-subjects three-way interaction effect was obtained in a repeated measures ANOVA with maltreatment, gender and externalizing cases as independent variables.”  We send an email to Dr. Cicchetti. (‘cicchett@umn.edu’) He replied: Here are the raw cortisol values for the meta-analysis.  Nonmaltreated Group: Raw cortisol values in μg/dl                                    Morning Cortisol              Afternoon Cortisol                                      M (SD)                                  M (SD)  Girls                      .345 (.189)                           .236 (.120)  Boys                      .313 (.187)                           .221 (.117)    I calculated the sample size assuming that the girl/boy ratio equaled the ratio in the total sample: 225/371 🡪 61% boys  Sample size per gender in nonmaltreated group 0.61*204= 124 boys and 80 girls. |  |
| Cieslak [15] | | | |
| **Methods** | **Design** Prospective, observational study | | |
|  | **Setting** Ontario, Canada | | |
|  | **Timing** Not described | | |
| **Participants** | ***n=*** 61 | | |
|  | **Subjects** “Subjects were recruited from three schools in Southwestern Ontario. Subjects came from classes of students from randomly selected schools that agreed to participate.” | | |
|  | **Age** 10.4 ± 0.4 yr | | |
|  | **Sex** 29 boys 32 girls | | |
|  | **Exclusion criteria** “chronic medical conditions such as asthma, heart disease, or any other condition that would put the subject at risk when performing the experimental tests, and a flu vaccination in the past 12 mo.” | | |
| **Protocol** | “Resting saliva samples were collected and tested for resting cortisol and resting secretory immunoglobulin A (SIgA).” | | |
| **Outcomes** | **Primary** “relationships between mucosal immunity, physical fitness levels, stress levels, and relative body fat in 10- to 11-yr-old children.” | | |
|  | **Secondary** “to test the long-term reproducibility of both resting salivary cortisol and IgA for repeated measures taken 6 wk apart.” | | |
| **Results** | \| Salivary cortisol in male and female children \| \| \| \| --- \| --- \| --- \| \| Variables \| Boys \| Girls \| \| *n* \| 29 \| 32 \| \| Salivary cortisol, nmol/L \| 3.0 ± 0.5 \| 3.0 ± 0.3 \|   “There were no gender differences in SIgA, URTI frequency, and cortisol levels.” | | |

| Colomina 1997 [16] | |
| --- | --- |
| **Methods** | **Design** Prospective, observational study |
|  | **Setting** Reus, Spain |
|  | **Timing** 7 – 9am |
| **Participants** | ***n =*** 109 |
|  | **Subjects** “with a rather high average socio-economic status.” |
|  | **Age** “17.5 – 18.5 years” |
|  | **Sex** 37control boys, 13 affected boys, 36 control girls, 22 affected girls |
|  | **Exclusion criteria** only if salivary samples were not returned. |
| **Protocol** | *Schedules for Clinical Assessment in Neuropsychiatry* (SCAN) interviews were done by one psychiatrist and two clinical psychologists. Salivary samples were collected between 7am and 9am. Although not clearly described, saliva collection seems to have taken place at home, prior to the interview. |
| **Outcomes** | **Primary** Differences in cortisol values between boys and girls with and without psychiatric disorders |
|  | **Secondary** Cortisol levels for subjects affected with only one disorder or with comorbid disorders |
| **Results** | \| Salivary cortisol levels (µg/dL) \| \| \| \| \| \| \| \| --- \| --- \| --- \| --- \| --- \| --- \| --- \| \|  \| *n* \| Males \| *n* \| Males \| *n* \| Total \| \| Control group \| 37 \| 0.497 ± 0.278 \| 36 \| 0.486 ± 0.276 \| 73 \| 0.495 ± 0.271 \| \|  \|  \|  \|  \|  \|  \|  \| |

| Covelli 2012 [17] | |
| --- | --- |
| **Methods** | **Design** Non-randomized intervention study |
|  | **Setting** Florida, USA |
|  | **Timing** 8 – 10am |
| **Participants** | ***n=*** 106 |
|  | **Subjects “**Participants were recruited from a historically African American high school (9^th^ – 12th grades) with student population of 1000, located in an urban, low socioeconomic community in Florida. “ |
|  | **Age** 15.3 ± 1.1 yr |
|  | **Sex** 49 males and 57 females |
|  | **Exclusion criteria** “a history of peripheral circulation problems or diabetes “ |
| **Protocol** | “Biologic measurements (i.e. salivary cortisol before/after cold pressor test) were conducted by the study investigator during week 1 and week 4 of the study in a quiet area between the hours of 0800 and 1000” |
| **Outcomes** | **Primary** What is the prevalence of biologic measures of risk of hypertension specifically FHH, prehypertension, elevated salivary cortisol, hyperresponsive cortisol, and CVR? |
|  | **Secondary** Are there gender difference in the prevalence of biologic risk factors of hypertension? |
| **Results** | \| Biologic measures of risk of hypertension data. \| \| \| \| \| --- \| --- \| --- \| --- \| \|  \| Total \| Male \| Female \| \| *n* \| 106 \| 49 \| 57 \| \| Cortisol, nmol/L \| 26.3 (11.5) \| 27 (11) \| 25 (11.2) \| |

| Daughters 2013 [18] | |
| --- | --- |
| **Methods** | **Design** Non-randomized intervention study |
|  | **Setting** North Carolina, USA |
|  | **Timing** 3 – 5pm |
| **Participants** | ***n=*** 132 |
|  | **Subjects** were recruited via newspaper advertisements and via letters sent to guardians of all high school students in the local county |
|  | **Age** 16.1 ± 1.0 yr |
|  | **Sex** 73 females, 59 males |
|  | **Exclusion criteria** the use of corticosteroids or regular smoking in the past 30 days |
| **Protocol** | “participants completed a 10 min deep breathing exercise. The first cortisol sample (C1) was collected approximately 10 min after the relaxation exercise.” |
| **Outcomes** | **Primary** “evaluate how psychological stress, gender and HPA axis functioning relate to adolescent risk behavior.” |
|  | **Secondary** the interaction between gender and cortisol levels. |
| **Results** | \| Gender differences in salivary cortisol (nmol/L) \| \| \| \| --- \| --- \| --- \| \|  \| Male (*n* = 59) \| Female (*n* = 73) \| \| Baseline cortisol \| 3.22 (2.13) \| 2.30 (1.38) \| |
|  |  |

| Davis 1995 [19] | |
| --- | --- |
| **Methods** | **Design** Non-randomized intervention study |
|  | **Setting** Atlanta, USA |
|  | **Timing** 8.30 – 19h, mean 14.00h (1 to 3h after feeding) |
| **Participants** | ***n=*** 36 |
|  | **Subjects** Healthy term infants |
|  | **Age** 2 days old |
|  | **Sex** 18 male infants and 18 female infants |
|  | **Exclusion criteria** “Apgar score< 8 at 5min, risk for sepsis or on antibiotics at time of testing” |
| **Protocol** | **Protocol** “Saliva was collected using an aspirator that produced 100-mmHg vacuum pressure through a 35-cm (length) Delee suction catheter (Vygon). The catheter deposited saliva directly into a 4-cc plastic test tube. Saliva was collected five times: 5 min before NBAS ( — 5 min), immediately following NBAS (+ 1 min); 10 min after the beginning of the -I-1 min sample ( 4-10 min), immediately following electrode removal (+ 11 min), and 5 min after the beginning ofthe +1 min sample (4-15 min). One drop of a 5% citric acid solution was placed on the infant s tongue, to promote salivation” |
| **Outcomes** | **Primary** Sex differences in adrenocortical activity following administration of the NBAS (neonatal behavorial assessment scale) |
|  | **Secondary** Sex differences in cardiovascular and behavioural activity following administration of the NBAS |
| **Results** | No significant difference in baseline salivary cortisol levels 1.09 ± 1.32 µg/dL in males and 1.07±1.19 µg/dL in females |

| De Bruijn 2009 [20] | |
| --- | --- |
| **Methods** | **Design** case-control study |
|  | **Setting** Tilburg, The Netherlands |
|  | **Timing** home visits were divided into three groups: 10 – 12 am, 1– 3 pm, 3 – 5 pm |
| **Participants** | ***n=*** 45 |
|  | **Subjects** “For the comparison group (prenatally nonexposed), children were selected whose mothers had at least given information in two separate periods during pregnancy and did not report high scores for any of the prenatal depression or anxiety questionnaires.” |
|  | **Age** ± 40 months |
|  | **Sex** 19 boys and 26 girls |
|  | **Exclusion criteria** “Cases that had fathers who did not want to cooperate, single mothers or children with perinatal problems like prematurity and twins.” |
| **Protocol** | Child saliva was collected at the start of a home visit (T1), 22 min after a mother-child interaction episode (T2), and 22 min after a potentially frustrating task (T3). |
| **Outcomes** | **Primary** Differences in cortisol responses in preschool children prenatally exposed to high or low levels of maternal emotional complaints. |
|  | **Secondary** NA |
| **Results** | \| Cortisol levels (Log scores) at T1 (Basal) \| \| \| \| \| \| --- \| --- \| --- \| --- \| --- \| \|  \| Exposed \| \| Nonexposed \| \| \|  \| Boys (*n* =25) \| Girls (*n* =24) \| Boys (*n* =19–22) \| Girls (*n* =27–29) \| \| Mean (SD) \| 0.37 (0.29) \| 0.60 (0.42) \| 0.45 (0.27) \| 0.39 (0.26) \|   Sample T1 was included in the meta-analysis. The data were exponentiated  No dimension of measurement, e.g. nmol/L, is given. Therefore I emailed Prof. van Baar (A.L.vanBaar@uu.nl). She confirmed that the log transformed cortisol levels were measured in nmol/L. |

| Dietrich 2013 [21] | |
| --- | --- |
| **Methods** | **Design** prospective, observational study (TRAILS cohort) |
|  | **Setting** Groningen, The Netherlands |
|  | **Timing** immediately after waking up |
| **Participants** | ***n=*** 2230 |
|  | **Subjects** “five municipalities in the North of The Netherlands (including urban and rural areas) were requested to provide information from the community registers (i.e. name, date of birth, gender, address) of all inhabitants that were born between 1 October 1989 and 30 September 1990 (first two municipalities) or between 1 October 1990 and 30 September 1991 (last three municipalities) (…) A total of 135 primary schools were identified, encompassing 3483 eligible children.” |
|  | **Age** 11.1 ± 0.55 yr |
|  | **Sex** 1133 girls and 1097 boys |
|  | **Exclusion criteria** severe mental retardation, serious physical illness or handicap or no Dutch-speaking parent available, the use of corticosteroid-containing medication, lack of compliance with the protocol and extreme cortisol values.  Lack of compliance was defined as failing to take the first sample within 5 min of awakening or the second sample between 25 and 35 min after awakening. |
| **Protocol** | Subjects collected saliva at home immediately after waking up as they were still lying in bed (Cort1) and 30 min later (Cort2)” |
| **Outcomes** | **Primary** “The overall objective of the study is to contribute to the understanding of the determinants of adolescents’ mental (ill-) health and social development during adolescence and young adulthood”[22] |
|  | **Secondary** “the mechanisms underlying the associations between determinants and these outcomes.” [22] |
| **Results** | \|  \|  \| Boys \| Girls \| \| --- \| --- \| --- \| --- \| \| Cort1 (nmol/L) \| Mean (SD) \| 11.20 (4.70) \| 11.93 (4.73) \| \|  \| range \| 0.71 – 30.14 \| 0.87 – 13.14 \|   *n* = 1604 |

| Elmlinger 2002 [23] | | |
| --- | --- | --- |
| **Methods** | **Design** Cross-sectional study | |
|  | **Setting** Bad Nauheim, Germany | |
|  | **Timing** not described | |
| **Participants** | ***n=*** 762 | |
|  | **Subjects** neonates, infants, children, adolescents and young adults from different centers, as described previously (University Children’s Hospital, Tübingen, Germany; Sankt Bernward Krankenhaus, Hildesheim, Germany, and at the Medical Center, University of Sibiú, Romania.[24]).  No symptoms of any endocrine or metabolic disorders were found in the subjects. | |
|  | \| Age ranges of the healthy subjects \| \| \| \| --- \| --- \| --- \| \| Age \| Male \| Female \| \| 1 – 7d \| 28 \| 17 \| \| 8 – 15d \| 20 \| 20 \| \| 16d – 3y \| 42 \| 44 \| \| 4 – 6y \| 28 \| 23 \| \| 7 – 8y \| 26 \| 24 \| \| 9 – 10y \| 31 \| 40 \| \| 16d – 10y \| 127 \| 131 \| \| 11y \| 22 \| 23 \|   **Age**   \|  \| \| \| \| --- \| --- \| --- \| \| Age \| Male \| Female \| \| 12y \| 17 \| 18 \| \| 13y \| 21 \| 25 \| \| 14y \| 32 \| 30 \| \| 15y \| 40 \| 48 \| \| 16y \| 31 \| 40 \| \| 17y \| 22 \| 30 \| \| 18 – 19y \| 8 \| 12 \| \| 17 – 19y \| 30 \| 42 \| | |
|  | **Sex** 393 males and 369 females | |
|  | **Exclusion criteria** “As far as it was known, girls taking contraceptives were excluded from the study.”[24] | |
| **Protocol** | “In all centres sampling was done by venipuncture into serum tubes (Sarstedt, Nümbrecht, Germany), avoiding haemolysis.” [24] | |
| **Outcomes** | **Primary** to establish reference ranges for children (neonates to young adults), for serum lutropin (LH), follitropin (FSH), estradiol (E2), progesterone, prolactin, sex hormone-binding globulin (SHBG), dehydroepiandrosterone sulfate (DHEAS), cortisol and ferritin | |
|  | **Secondary** NA | |
| **Results** | We included the most representative and largest age group of the 1-8yr group (16d-3y, no cord blood included) and the youngest group of the >8yr-olds (11y) in the meta-analysis. Cortisol in nmol/L:   \| Age \| Male \| \| \| Female \| \| \| \| --- \| --- \| --- \| --- \| --- \| --- \| --- \| \|  \| 2.5^th^ \| 50^th^ \| 97.5^th^ \| 2.5^th^ \| 50^th^ \| 97.5^th^ \| \| 1 – 7d \| 213 \| 524 \| 961 \| 204 \| 569 \| 927 \| \| 8 – 15d \| 190 \| 311 \| 581 \| 145 \| 342 \| 613 \| \| 16d – 3y \| 111 \| 253 \| 486 \| 155 \| 271 \| 537 \| \| 4 – 6y \| 109 \| 253 \| 673 \| 139 \| 218 \| 601 \| \| 7 – 8y \| 194 \| 400 \| 749 \| 113 \| 294 \| 597 \| \| 9 – 10y \| 98 \| 317 \| 748 \| 123 \| 328 \| 724 \| \| 11y \| 133 \| 341 \| 578 \| 156 \| 364 \| 616 \| \| 12y \| 233 \| 386 \| 681 \| 153 \| 388 \| 707 \| \| 13y \| 232 \| 430 \| 746 \| 216 \| 442 \| 621 \| \| 14y \| 216 \| 440 \| 644 \| 224 \| 466 \| 740 \| \| 16y \| 179 \| 444 \| 618 \| 240 \| 388 \| 697 \| \| 17 – 19y \| 218 \| 465 \| 707 \| 242 \| 403 \| 648 \|   In addition, 95%CIs were converted to SDs: upper limit – lower limit /3.92 [5] | |
| Forest 1978 [25] | | |
| **Methods** | | **Design** Non-randomized intervention study |
|  |  | **Setting** Lyon, France |
|  |  | **Timing** 8 – 9 am |
| **Participants** | | ***n=*** 20 infants |
|  |  | **Subjects** not described how they were selected |
|  |  | **Age** <1 yr |
|  |  | **Sex** 17 males and 3 females |
|  |  | **Exclusion criteria** pituitary or adrenal dysfunction |
| **Protocol** | | “A standardized adrenal function test” |
| **Outcomes** | | **Primary** The response of Testosterone, ∆^4^ –androstenedione and cortisol to prolonged ACTH stimulation, from infancy to adulthood |
|  |  | **Secondary** Age- and sex-specific differences |
| **Results** | | \| I edited the data from table 1: \| \| \| \| \| \| \| --- \| --- \| --- \| --- \| --- \| --- \| \| Cortisol µg/dL \| *n* \| Minimum \| Maximum \| Mean \| SD \| \| Males \| 15 \| 2.90 \| 21.00 \| 11.3000 \| 6.07207 \| \| Females \| 3 \| 11.10 \| 23.90 \| 16.2333 \| 6.76560 \|   Males: 311.8 ± 167.5 nmol/L Females: 447.9 ± 186.7 nmol/L |

| Fransson et al. 2014 [26] | |
| --- | --- |
| **Methods** | **Design** Prospective, observational study |
|  | **Setting** Stockholm, Sweden |
|  | **Timing** at awakening |
| **Participants** | ***n =***  132 (living in intact families) |
|  | **Subjects** Adolescents aged 14 – 16 years were recruited through two compulsory schools in Stockholm, Sweden, to participate in a study of students’ daily life, health and well-being in the 8th and 9th school years. |
|  | **Age** 14 – 16yr |
|  | **Sex** 89 girls and 43 boys |
|  | **Exclusion criteria** “Relatively few adolescents lived with one parent only and since their contacts with the other parent varied, these adolescents were excluded (n = 49). Also, data from adolescents suffering from chronic illness relating to hormonal functioning or taking medication influencing HPA-axis functioning (e.g., asthma and hypothyroidism) were excluded (n = 3).“ |
| **Protocol** | **Protocol “**Salivary cortisol samples were collected: 1) immediately at awakening, 2) +30 minutes, 3) +60 minutes, and 4) at 8 pm” |
| **Outcomes** | **Primary** “to investigate how living arrangements (intact family/JPC) relate to HPA-axis activity and recurrent pain in mid-adolescents.” |
|  | **Secondary** the influence of sex and school year |
| **Results** | “means (SD) for cortisol (log transformed values) at different time points for the different groups were as follows; Girls in intact families: at waking: 2.39 (SD= .70) (…)Boys in intact families: at waking: 1.88 (SD= .71)”  I have emailed Fransson ('emma.fransson@ki.se') to check if it was the natural logartithm. She confirmed that. Data were exponentiated. |

| Garagorri 2008[27] | |
| --- | --- |
| **Methods** | **Design** Longitudinal, observational study |
|  | **Setting** Zaragoza, Spain |
|  | **Timing** 8am – 9am |
| **Participants** | ***n=*** 138 |
|  | **Subjects** “37 – 41 gestational weeks, birth weight 2,500 – 4,000 g, (…) and spontaneous and uncomplicated delivery (…) Moreover, all these infants had a normal weight and length evolution without any disease during their first 6 months of life. Infants of minority ethnic groups were not considered.” |
|  | **Age** 0 – 6 months |
|  | **Sex** 80 males and 58 females |
|  | **Exclusion criteria** “Major congenital and metabolic abnormalities, maternal endocrine and non-endocrine diseases that could affect newborn neurohormonal status, and history of maternal corticoid therapy (…) no neonatal asphyxia or perinatal diseases, pregnancy without incidents” |
| **Protocol** | “Blood samples were obtained from each infant between 8:00 am and 9:00 am by rapid venous puncture six times during the first 6 months of life” |
| **Outcomes** | **Primary** “to obtain longitudinal reference plasma levels for 17-hydroxyprogesterone (17OHP), 11-desoxycortisol (11DOC), cortisol, dehydro-epiandrosterone sulfate (DHEAS), testosterone, and androstenedione in healthy infants from birth to 6 months of age.” |
|  | **Secondary** NA |
| **Results** | \|  \|  \| All (*n =* 138) \| \| Boys (*n =* 80) \| \| Girls (*n =* 58) \| \| \| --- \| --- \| --- \| --- \| --- \| --- \| --- \| --- \| \|  \| Age \| Mean±SD \| Range \| Mean±SD \| Range \| Mean±SD \| Range \| \| Cortisol (µg/dL) \| 3 days \| 9.51±4.09 \| 23.5–2.3 \| 9.04±3.6 \| 21.6–2.3 \| 10.16±4.5 \| 23.5–2.4 \|   The collection at 3 days of age was included in the meta-analysis, to minimize the influences of the delivery, while selecting the “youngest” and most relevant sample. |

| Georgopoulos 2011 [28] | |
| --- | --- |
| **Methods** | **Design** Case-control study |
|  | **Setting** Athens, Greece |
|  | **Timing** in the morning, before brushing their teeth, smoking, drinking, or eating |
| **Participants** | ***n=*** 81 controls |
|  | **Subjects** Controls did not engage in strenuous sports activities |
|  | **Age** girls aged 16.0±1.4 years; boys aged 15.3±2.0 years |
|  | **Sex** 40 girls, 41 boys |
|  | **Exclusion criteria** Engaging in strenuous sports activities |
| **Protocol** | Saliva was collected in the morning, during a normal school week |
| **Outcomes** | **Primary** “to evaluate the effects of intensive physical exercise and acute psychological stress during a high level athletic competition on the levels of salivary cortisol in elite artistic gymnasts.” |
|  | **Secondary** gender influences |
| **Results** | “In the control group, salivary morning cortisol was 11.6±8.3 nmol/l (*n=*29) in females and 9.1±8.3 nmol/l (*n=*35) in males” |

| Ghaziuddin 2003 [29] | |
| --- | --- |
| **Methods** | **Design** Placebo-controlled cross-over trial |
|  | **Setting** Michigan, USA |
|  | **Timing** 7 am |
| **Participants** | ***n =***  21 |
|  | **Subjects** healthy adolescents |
|  | **Age** 14.6 ± 1.5 years |
|  | **Sex** 9 females and 12 males |
|  | **Exclusion criteria** “Any psychiatric diagnosis on Axis I or on Axis II, a first-degree relative with a psychiatric disorder, pregnancy, lactation, drug abuse or dependence, major medical disorder, and mental retardation or pervasive developmental disorder.” |
| **Protocol** | a challenge with the central serotonergic agonist m-chlorophenylpiperazine (mCPP) : “Subjects received 0.1mg/kg mCPP in saline, infused over 90 s under conditions identical to the saline infusion (…) Blood samples were collected at 10 and 3 min before infusion and at 20, 35, 50, 95, and 120 min after saline infusion.” |
| **Outcomes** | **Primary** “identify the effects of mCPP among adolescents” |
|  | **Secondary** “determine if serotonin-related neuroendocrine hormones (prolactin, GH and cortisol) are influenced by gender.” |
| **Results** | \| Baseline hormone levels (Mean ± SD) in adolescent males and females \| \| \| \| --- \| --- \| --- \| \| Cortisol \| *n* \| Pre-saline \| \| Female \| 8 \| 69.4 ± 60.1 \| \| Male \| 12 \| 63.7 ± 43.2 \|   No dimension of measurement, e.g. nmol/L, is given. Moreover, the concentrations were either low (when measured in nmol/L) or very high (when measured in µg/dL). Therefore I emailed Dr. Ghaziuddin (neerag@med.umich.edu). She replied:  Hi, thank you for your interest in this article.  I no longer have the raw data pertaining to this paper but I had worked with a very diligent statistician and I am certain that what we reported was what we had found.  Good luck with your review.  Neera Ghaziuddin, MD.  Associate Professor of Psychiatry  University of Michigan, Ann Arbor  Based on the unit of measurement shown in figure 2, we assumed that it was measured in µg/dL. |

| Gunnar 2010 [30] | |
| --- | --- |
| **Methods** | **Design** Cross-sectional study |
|  | **Setting** Minnesota, USA |
|  | **Timing** between 10 and 11 am |
| **Participants** | ***n =***  151 |
|  | **Subjects** “recruited from family-based day-care settings in a major metropolitan area.“ |
|  | **Age** 3.81 ± 0.23 yr |
|  | **Sex** 82 girls and 69 boys |
|  | **Exclusion criteria** “being the child of the care provider” |
| **Protocol** | **Protocol** “The children were observed on two mornings between 8:30 and 9:30 am on days the provider deemed typical (i.e., no field trip, birthday parties, etc.) (…) Saliva samples were collected by the day-care provider on 2 days between 10:00 and 11:00 am and 3:00 and 4:00 p.m (…) Parents were also asked to collect samples using the same protocol on 2 days when the child did not attend day care. (…) Parents followed the same protocol as the day-care providers” |
| **Outcomes** | **Primary** “determine whether cortisol increases of the day in family day-care settings” |
|  | **Secondary** (1) examine the extent to which structural and process measures of child-care quality are associated with the magnitude of cortisol increase over the child-care day; (2) explore whether anxious, vigilant or angry, aggressive behavior is related to the child-care cortisol stress response; (3) examine whether gender moderates child behavior–cortisol rise associations; (4) examine whether child behavior mediates or moderates associations between process measures of child-care quality and the child-care cortisol stress response. |
| **Results** | Cortisol concentrations were not reported for males and females separately.  We send an email to Dr. Gunnar (‘gunnar@umn.edu’) She replied:  Dear Bibian  Here are the data. I did not try to impute so there are missing subjects. What you have as months is our first assessment and 6 months later. Then dcamt1 is day care am (morning) time 1. So, ampm is morning and afternoon. you do have some trends, but nothing significant. The 3 year olds tend to respond more than the four year olds, as I have reported previously.  Megan  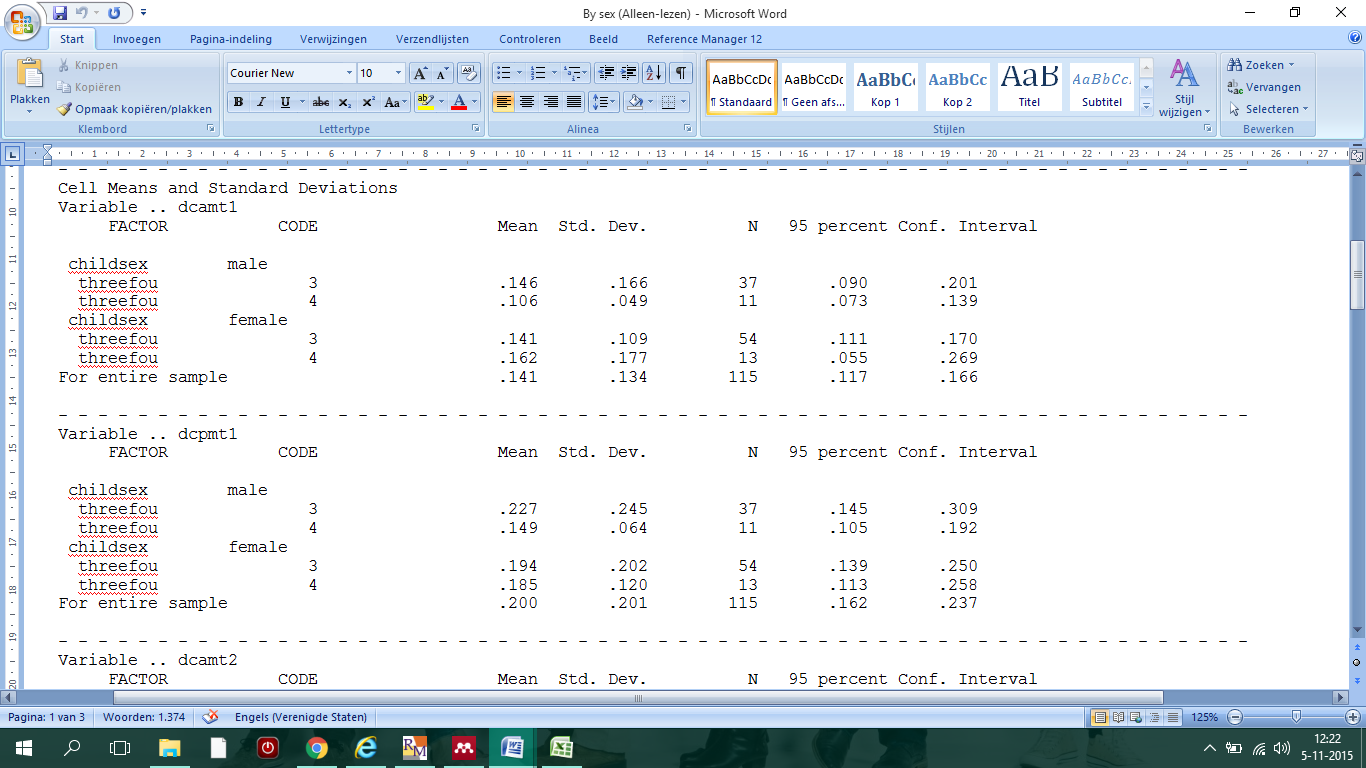 |

| Hackney 2003 [31] | |
| --- | --- |
| **Methods** | **Design** Prospective, observational study (CHIC cohort) |
|  | **Setting** North Carolina, USA |
|  | **Timing** 8 am |
| **Participants** | ***n =*** 125 |
|  | **Subjects** “The subjects were part of an ongoing epidemiological study to evaluate cardiovascular disease risk factors in children (CHIC Study)” |
|  | **Age** 13.4 ± 0.9 yr |
|  | **Sex** 63 girls and 62 boys |
|  | **Exclusion criteria** inability to read and write English; mental, emotional, or physical handicap; chronic illness |
| **Protocol** | “All physiologic measurements were collected “on-site” at the subjects’ schools. All physical tests and research procedures were completed in the same order, which was (1) resting blood collection, (2) questionnaires, (3) height, (4) weight, (5) skinfold measurements, and (6) PWC195 cycle ergometry test. All blood samples were obtained after an overnight fast (8h) by using venipuncture techniques performed by a trained phlebotomist.” |
| **Outcomes** | **Primary** “the interrelationships between the hormones leptin, TSH, free T3, and cortisol” |
|  | **Secondary** “the interrelationships between (…) caloric intake, body composition, and physical activity to these select hormones“ |
| **Results** | \| Resting hormonal levels of the 125 subjects used in the study (mean ± SD) \| \| \| \| --- \| --- \| --- \| \|  \| Male (*n* = 62) \| Female (*n* = 63) \| \| Cortisol (µg/dL) \| 14.4 ± 4.6 \| 16.9 ± 5.9 \| |

| Honour et al. 2007 [32] | |
| --- | --- |
| **Methods** | **Design** Longitudinal, cohort study (ALSPAC birth cohort) |
|  | **Setting**  London, United Kingdom |
|  | **Timing** “Urine collection for 14 h, from 2200 to 1200 h the following day” |
| **Participants** | ***n =***  461 |
|  | **Subjects** “The ALSPAC birth cohort includes 14,062 live births, comprising over  80% of all births in the three Bristol-based District Health Authorities between April 1991 and December 1992 (…) Most infants were born at term. A 10% subcohort (Children in Focus) was randomly selected from the tail end of births in ALSPAC to undergo regular postnatal measurements (at 4, 8, 12, 18, 24, 31, 37, 43, 49, and 61 months) (…)In an additional subset of children (control group), direct measurements of growth are also available from early childhood. Together these subsets consist of approximately 1800 children. The whole cohort has attended annual half-day clinics from the age of 7 yr.” |
|  | **Age** 8.2 – 8.4 yr |
|  | **Sex** 244 boys and 188 girls |
|  | **Exclusion criteria** any form of steroid medication taken in the past year. |
| **Protocol** | **“**Urine collection for 14 h, from 2200 to 1200 h the following day (...) Collections were judged to be reliable when total volumes were between 300 and 750 ml and the creatinine concentrations were within the range 3.5 to 15 mmol/ liter [27]. Other samples were not processed for steroid excretion rates due to the likelihood of incomplete collections. “ |
| **Outcomes** | **Primary** the association between early growth (birth weight, early weight gain, current weight) and adrenal androgen excretion at 8 yr of age. |
|  | **Secondary** the association between adrenal androgen excretion at 8 yr of age and arterial blood pressure and insulin sensitivity. |
| **Results** | \|  \| Boys \| Girls \| \| --- \| --- \| --- \| \| Total cortisol metabolites (mg per 14h) \| 1649 (1128 – 2174) \| 1492 (1118 – 2003) \| \| Data are presented as median (interquartile range) \| \| \|   We calculated 24h cortisol production rates, based on these 14h collections.  **boys** 24/14 * 1649 = 2826.86  **girls** 24/14 * 1492 = 2557.71  In addition, we converted interquartile ranges to SDs by calculating:  upper limit – lower limit /1.35 [5]  **boys** 2826.86 ± 774.81 mg/24h  **girls** 2557.71 ± 655.56 mg/24h |

| Huybrechts 2014 [33] | |
| --- | --- |
| **Methods** | **Design** cross-sectional study (HELENA study) |
|  | **Setting** Europe; from 10 European cities: Stockholm, Sweden; Ghent, Belgium; Lille, France; Dort- mund, Germany; Vienna, Austria, Pécs, Hungary; Rome, Italy, Athens and Crete, Greece, Zaragoza, Spain. |
|  | **Timing** morning blood samples |
| **Participants** | ***n=*** 723 |
|  | **Subjects** “Adolescents were selected by random cluster sampling [all pupils from a selection of classes from all schools in 10 European cities (…) and stratified by geographical location, age and socio-economic status (…)Blood sampling was performed in a randomly representative sample of one third of the participants (100 adolescents per city), based on class level“ |
|  | **Age** 14.7 ± 1.2 yr |
|  | **Sex** 297 boys and 426 girls |
|  | **Exclusion criteria** Male or female subjects aged <12.5 or ≥17.5 years, or when weight and/or height information was missing [34] |
| **Protocol** | “Participants who were selected for the blood sampling were asked to abstain from eating and drinking after 8 pm the day before the study. On the day of the study, a medical doctor either visited the school classes (in Athens, Dortmund, Ghent, Heraklion, Pecs, Roma, Stockholm, Vienna, Zaragoza), or participants went to a hospital ward (in Lille). Participants were asked for their medical history and recent acute diseases. A blood sampling questionnaire was used to assess fasting status, acute infections, allergies, smoking, vitamin and mineral supplements, and medication (…)Blood sampling generally took place between 8–10 a.m, after blood pressure measurement and body composition assessment by bio-impedancemetry” [34] |
| **Outcomes** | **Primary** “the present study investigates the relationships between serum cortisol and leptin, insulin, glucose and IR (the latter by Homeostasis Assessment Model for Insulin Resistance (HOMA-IR)) in European adolescents” |
|  | **Secondary** gender differences |
| **Results** | \|  \| Total (*n* = 723) \| Boys (*n* = 297) \| Girls (*n* = 426) \| \| --- \| --- \| --- \| --- \| \|  \| Mean ± SD \| Mean ± SD \| Mean ± SD \| \| Cortisol (nmol/L) \| 303.1 ± 1.6 \| 299.5 ± 1.6 \| 305.6 ± 1.7 \| |

| Ilias 2009 [35] | |
| --- | --- |
| **Methods** | **Design** Non-randomized intervention study |
|  | **Setting** Athens, Greece |
|  | **Timing** 24h-plasma withdrawal |
| **Participants** | ***n=*** 11 |
|  | **Subjects** “Eleven prepubertal children (…) with idiopathic short-normal stature and normal GH responses to at least one pharmacological stimulation test (peak GH >10 ng/ml after arginine or clonidine administration) as well as normal IGF-I, IGF-binding protein-3, and 24h integrated GH plasma concentrations were studied.” |
|  | **Age** ± 9 yr |
|  | **Sex** 5 boys and 6 girls |
|  | **Exclusion criteria** peak GH response <10 ng/ml after arginine or clonidine administration, abnormal IGF-I, IGF-binding protein-3, and 24-h integrated GH plasma concentrations |
| **Protocol** | “At 10:00 am, after an overnight fast, an indwelling non-thrombogenic catheter was inserted into an antecubital vein and connected to a portable constant withdrawal pump, according to the method of Kowarski et al. The rate of withdrawal was 4 ml/h and blood collection tubes were changed every 30min for 24h. During this time the children were encouraged to continue normal activity and were given a standard hospital diet. We started the pulsatility study at 10:00 am to avoid dividing the morning cortisol surge.” |
| **Outcomes** | **Primary** “The aim of the present study was to evaluate using the tools of ApEn and cross-ApEn the regularity of Le, F, and GH secretions” |
|  | **Secondary** “the joint synchrony of the secretory profiles of these hormones under physiological conditions” |
| **Results** | \| All values are mean ± SD \| \|  \| \| --- \| --- \| --- \| \|  \| Boys (*n* = 5) \| Girls (*n* = 6) \| \| Cortisol (nmol/L) \| 112.97 ± 49.14 \| 153.26 ± 131.05 \|   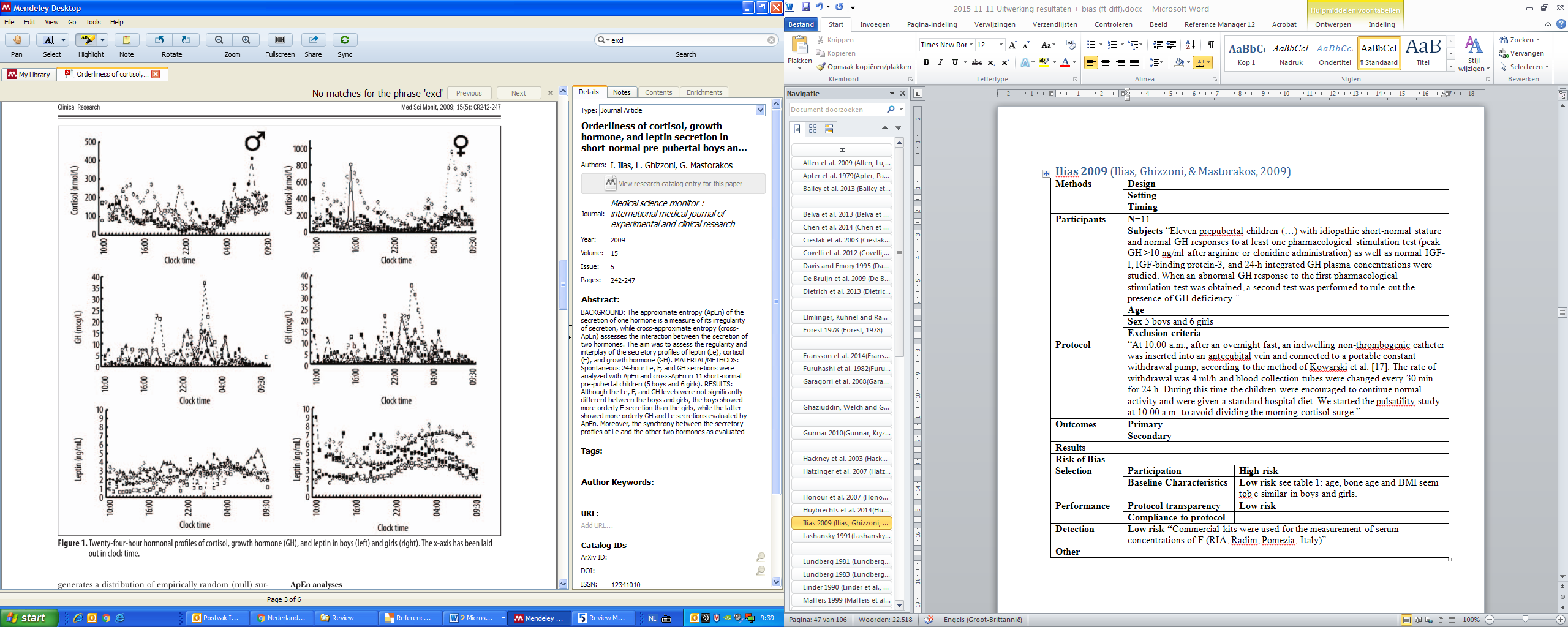  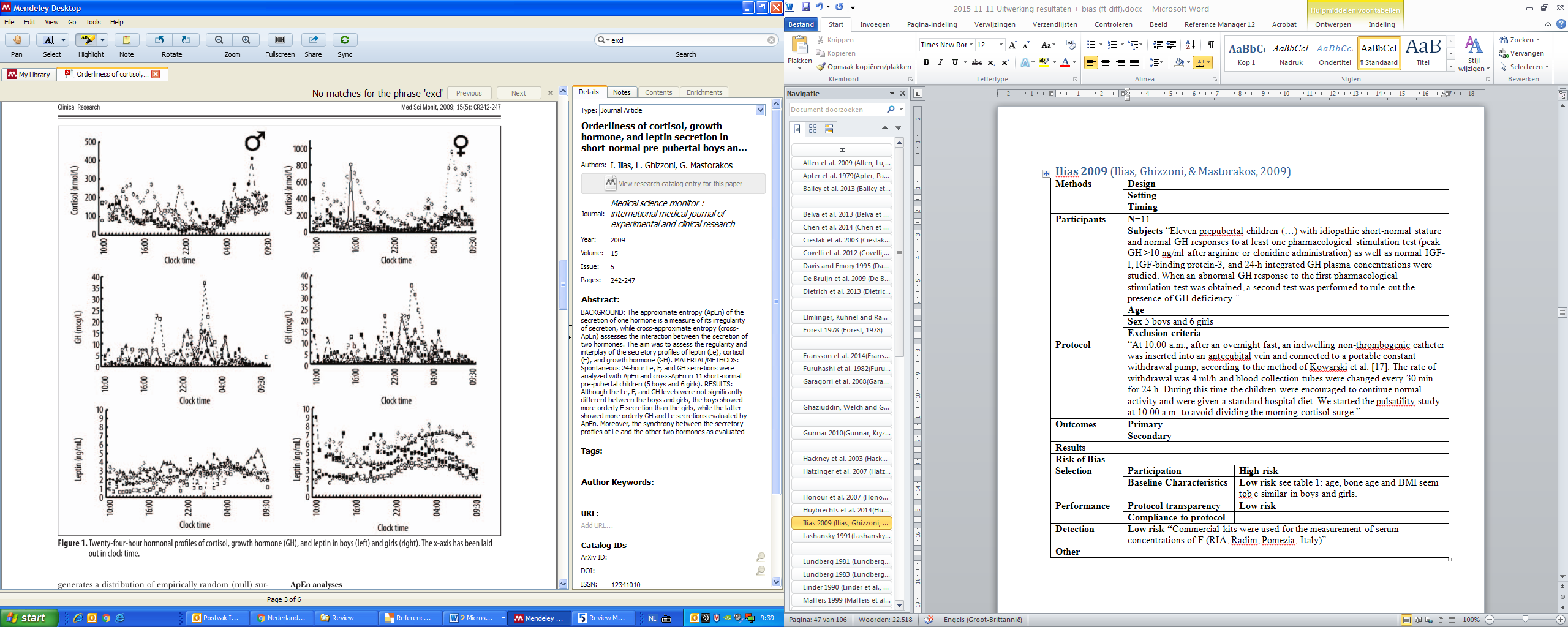 |

| **Jones 2006** [36] | | |  |
| --- | --- | --- | --- |
| **Methods** | | **Design** Prospective, observational study |  |
|  |  | **Setting** Southampton, United Kingdom |  |
|  |  | **Timing** first sample on awakening |  |
| **Participants** | | ***n=*** 140 |  |
|  |  | **Subjects** “healthy children (68 boys and 72 girls, aged 7 – 9 yr) who have been followed up since 12 wk gestation when their mothers took part in a study of children born in Southampton, UK” |  |
|  |  | **Age** 7 – 9 yr |  |
|  |  | **Sex** 68 boys and 72 girls |  |
|  |  | **Exclusion criteria** not described |  |
| **Protocol** | | “To assess their baseline adrenocortical function, the children were asked to use a home-testing kit to collect salivary cortisol at five time points [on awakening, 30 min later, 1230 h (before lunch), 1530 h, and 1830 h (before evening meal)] on a restful day (usually a weekend or holiday) when the children were taking no part in activities(...) the children attended a clinical research facility for the TSST-C, which was timed to occur in the afternoon (1330–1430 h) when diurnal secretion of cortisol is leveling out.” |  |
| **Outcomes** | | **Primary** “we examined the relationship between birth weight and HPAA stress responsivity.” |  |
|  |  | **Secondary** gender differences |  |
| **Results** | | See figure 1: “ Home profiles did not differ significantly between genders with the exception of the awakening response, which was evident in boys but not girls (P = 0.04 for difference in increment between awakening and 30 min later).”  Send email Okt 2015 to Dr. Philips, who forwarded the mail to the fellow in charge, Drs. Jones (alex.jones@ucl.ac.uk). His reply:  Dear Bibian  Sorry for the delay - things have been hectic.  I managed to dig out the data.  Is this what you are after?  ----------------------------------------------------  -> sex = 0  Variable \|   Obs   Mean    Std. Dev.  Min        Max  -------------+---------------------------------------------------------  hlcort1 \|   61    2.259149   .4128954   1.137581  3.062258  hlcort2 \|   61    2.403583  .5108586   .9359011  3.318198  hlcort3 \|   61    1.45859    .3199189   .8356615   2.28684  hlcort4 \|   61    1.3097     .415888   .3679596 2.184431  hlcort5 \|   59    .8277474   .4942733 0 1.770462  --------------------------------------------------------------  -> sex = 1  Variable \|  Obs   Mean     Std. Dev.    Min        Max  -------------+------------------------------------------------  hlcort1 \|   65    2.360082    .4049213   1.453928   3.203153  hlcort2 \|   64    2.344766    .4194042   1.276096   3.252982  hlcort3 \|   64    1.519448    .3553046   .6555833   2.233666  hlcort4 \|   65    1.337486    .4077579   .4087432   2.303958  hlcort5 \|   65    .7889093    .4670859          0   2.468669  Please note that sex = 0 is males and sex = 1 is females.  Also note that for the home saliva profiles, we had some missing data in both sexes - the N numbers are provided. The values are logged salivary cortisols at the five time points of the home profiles as per figure 1.  The exponentiated salivary cortisol level upon awakening (h1cort1) was included in the meta-analysis:  Males: 9.58± 1.51nmol/L Females: 10.59± 1.49nmol/L |  |
| Lashansky 1991[37] | | | |
| **Methods** | **Design** Non-randomized intervention study | | |
|  | **Setting** Santo Domingo, Dominican Republic | | |
|  | **Timing** 8 – 10 am | | |
| **Participants** | ***N=*** 102 | | |
|  | **Subjects** healthy children with an adequate nutritional status and growth | | |
|  | **Age** group 1: 2 – 12 month-old, *n=*22; group 2: 1-5yr old, *n=*22; group 3: 6-12yr old, *n=*15; group 4: early-midpuberty (Tanner stage 2-3), *n=*21; group 5: late puberty (Tanner stage 4-5), *n=*22. | | |
|  | **Sex** 43 females and 59 males | | |
|  | **Exclusion criteria** inadequate nutritional status and/or inappropriate growth | | |
| **Protocol** | **Protocol** “ACTH stimulation test was performed with a single IV injection of 0.25mg ACTH-(1-24)” | | |
| **Outcomes** | **Primary** “Adrenal steroids before and after ACTH stimulation.” | | |
|  | **Secondary** The need to interpretate ACTH stimulation test data to be based upon age- and sex-specific norms | | |
| **Results** | \|  \| **Baseline Cortisol levels (nmol/L)** \| **<1 yr** \| **1 – 5 yr** \| **6 – 12 yr** \| **Tanner**  **II – III** \| **Tanner**  **IV – V** \| \| --- \| --- \| --- \| --- \| --- \| --- \| --- \| \| **Males** \| **Mean ± SD** \| 342 ± 146 \| 337 ± 160 \| 254 ± 86 \| 223 ± 74 \| 262 ± 80 \| \| **Range** \| 83 – 579 \| 157 – 690 \| 157 – 414 \| 110 – 359 \| 138 – 414 \| \| **Females** \| **Mean ± SD** \| 353 ± 196 \| 281 ± 113 \| 229 ± 94 \| 243 ± 102 \| 279 ± 77 \| \| **Range** \| 116 – 634 \| 201 – 524 \| 83 – 331 \| 119 – 441 \| 166 – 414 \| | | |

| Lundberg 1981 [38] | |
| --- | --- |
| **Methods** | **Design** Longitudinal, cohort study |
|  | **Setting** Stockholm, Sweden |
|  | **Timing** urine samples were obtained at 7am (night urine), 10am, 12noon and 2pm |
| **Participants** | ***n =***  13 |
|  | **Subjects** “Fifty women, each of whom was expecting her first child and was experiencing a normal pregnancy, had been selected for a longitudinal study (...) An additional follow-up was conducted three years after delivery” |
|  | **Age** 3yr |
|  | **Sex** 9 girls and 4 boys |
|  | **Exclusion criteria** a complicated pregnancy or delivery |
| **Protocol** | **Protocol** “In each family the three-year-old, the mother and the father were asked to spend a day at the hospital for an examination, which included observations and testing of the child, an interview with both parents, and a videotape recording of mother-child play. Urine specimens were collected (…) during each of four different conditions at the hospital. To obtain reference levels, urine was also collected at home.“ |
| **Outcomes** | **Primary** “to examine catecholamine and cortisol excretion patterns in healthy three-year-olds, their mothers and their fathers during routine activities at home and under diverse conditions at the hospital” |
|  | **Secondary** the effects of differences in neonatal care |
| **Results** | \| **Cortisol excr. (pmol/min/kg)** \| 7 a.m. \| \| \| \| --- \| --- \| --- \| --- \| \| Mean \| SE \| *n* \| \| Girls \| 3.04 \| 0.54 \| 9 \| \| Boys \| 4.14 \| 1.39 \| 5 \|   We calculated 24h cortisol production rates, based on the 7am overnight collections. Since bodyweight of the children is not described, the bodyweight of a 3yr-old was estimated to be approximately (2,5*age+8=) 15.5kg  **boys** 4.14*60*24 = 3.35 ± 1.12 µg/24h  **girls** 3.04*60*24 = 2.46 ± 0.44 µg/24h |

| Lundberg 1983 [39] | |
| --- | --- |
| **Methods** | **Design** Prospective, observational study |
|  | **Setting** Karolinska, Sweden |
|  | **Timing** urine samples obtained between 8 am and 4 pm” |
| **Participants** | ***n=*** 26 |
|  | **Subjects** “Children who were at a day-care center in a Stockholm suburb, 9 hours each day, while their parents were at their jobs or studying. Fifteen of them had been attending the day-care center for more than a year, while eleven joined the center when the study started. According to consistent reports from the parents and nurses, all the eleven children were well adjusted to the day-care situation by the end of their second week.” |
|  | **Age** ± 4.5yr |
|  | **Sex** 15 boys and 11 girls |
|  | **Exclusion criteria** not described |
| **Protocol** | “At the day center, the urine was collected in white plastic buckets, which had been fitted into the toilets. Each time the child spontaneously went to the toilet to void, the experimenter noted the time and collected the urine. In order to make the children familiar with the procedure, the sampling of urine (not used in the analyses) was started one week prior to the actual study. In the at-home condition, urine samples were obtained by the parents, who noted the times for voiding and stored the urine for four hours (maximum) in a refrigerator at about +3°C until picked up by the experimenters. A subsample of 2 ml urine was removed from each specimen for cortisol analysis” |
| **Outcomes** | **Primary** “To examine sex differences in behaviour pattern and catecholamine and cortisol excretion in 3-6 year old children under natural conditions, i.e. during daily activities at a day-care center and in the children’s homes. “ |
|  | **Secondary** “In addition, catecholamine and cortisol excretion levels were determined in a group of new children, who were adjusting to the day-care situation.” |
| **Results** | \| **Cortisol excretion (pmol/min/m^2^)** \| Week number 3 \| \| \| \| --- \| --- \| --- \| --- \| \| Mean \| SE \| *n* \| \| Boys \| 122 \| 9.0 \| 8 \| \| Girls \| 171 \| 45.8 \| 3 \|   In the article it was noted that the children were well adjusted after 3 weeks. Therefore, we took the data of week 3 and calculated 24h cortisol production rates, based on their height and weight, assuming that their body surface area will be approximately 0.75 m^2^.  In addition, standard errors were converted to SDs: SD= SE x √N [5]  Cortisol excretion: **Boys** 8.5 ± 1.7 µg/24h **Girls** 11.9 ± 5.5 µg/24h |

| **Martikainen 2013** [40] | |
| --- | --- |
| **Methods** | **Design** Prospective, observational study |
|  | **Setting** Helsinki, Finland |
|  | **Timing “**7:53 am; SD 50 minutes |
| **Participants** | ***n =*** 258 |
|  | **Subjects** “From an urban community-based cohort comprising 1049 infants (...)were recruited to overrepresent children whose mothers consumed higher amounts of licorice” |
|  | **Age** 8 yr |
|  | **Sex** 126 boys and 132 girls |
|  | **Exclusion criteria** children with parent-reported, physician-diagnosed developmental delay (*n*=3) or Asperger syndrome (*n=*1) and those who did not provide at least 4days of valid PA data including at least 1 weekend day (*n=*54).  Of the remaining 263 children,11 in the diurnal sampling and 15 in the sampling during the TSST-C were excluded for having more than 1 missing cortisol value. |
| **Protocol** | “Parents were shown how to collect salivary samples for determination of cortisol using cotton swabs (Salivette, Sarstedt,Nümbrecht, Germany). Salivary samples were obtained during a 1-day period, at awakening (mean 7:53 am; SD 50 minutes), 15 and 30 minutes thereafter, and at 10:30 am, 12:00 pm, 5:30 pm, and bedtime (mean 9:15pm; SD 75minutes)” |
| **Outcomes** | **Primary** “First, we examined whether overall daytime physical activity (PA), referring to the child’s habitual activity level, was associated with diurnal salivary cortisol pattern“ |
|  | **Secondary** “Second, we examined whether overall daytime PA was associated with salivary cortisol responses to a standardized psychosocial stress test, the TSST for Children (TSST-C). Finally, we examined whether children who occupy a higher percentage of time in vigorous physical activity (VPA) showed different diurnal patterns of salivary cortisol and different salivary cortisol responses to stress.“ |
| **Results** | The salivary cortisol level upon awakening was included in the meta-analysis.   \|  \| Boys (*n* = 126)  Mean (SD) \| Girls (*n* = 132)  Mean (SD) \| \| --- \| --- \| --- \| \| Salivary cortisol (nmol/L) \|  \| \| \| Upon awakening \| 6.8 (1.7) \| 8.0 (1.7) \| |

| Michels 2012 [41] | |
| --- | --- |
| **Methods** | **Design** prospective, observational study (IDEFICS and ChiBS cohort) |
|  | **Setting** Ghent, Belgium |
|  | **Timing** at waking up |
| **Participants** | ***n =*** 385 |
|  | **Subjects** “The IDEFICS study aims to investigate the aetiology of major  Disorders (...) Control children were selected at random from the remaining cohort” [42] |
|  | **Age** Boys 8.44 ± 1.18 yr; Girls 8.39 ± 1.20 yr |
|  | **Sex** 183 boys and 202 girls |
|  | **Exclusion criteria** “morning samples collected more than 5 min different from  the requested time point and evening samples not collected between 1900 h and 2100 h, were excluded. Furthermore, samples of corticosteroid-users were also excluded.” |
| **Protocol** | **Protocol** “Salivary cortisol samples were collected when waking up, 30 min and 60 min after wake up and in the evening between 1900h and 2000h on two consecutive weekdays.” |
| **Outcomes** | **Primary** Are children’s life events, emotions and psychological difficulties related to their salivary cortisol patterns? |
|  | **Secondary** Gender differences |
| **Results** | The salivary cortisol level upon awakening was included in the meta-analysis.   \|  \| Boys (*n*=183*)* \| \| \| \| Girls (*n*=202*)* \| \| \| \| \| --- \| --- \| --- \| --- \| --- \| --- \| --- \| --- \| --- \| \| Salivary cortisol (nmol/L) \| Mean \| SD \| Min \| Max \| Mean \| SD \| Min \| Max \| \| Immediately after wake up \| 12.66 \| 6.12 \| 1.66 \| 56.00 \| 13.84 \| 8.96 \| 1.49 \| 76.41 \| |

| **Mills 2008** [43] | |
| --- | --- |
| **Methods** | **Design** Non-randomized intervention study |
|  | **Setting** Manitoba, Canada |
|  | **Timing** between 9 am and 7 pm |
| **Participants** | ***n*** = 200 |
|  | **Subjects** “Families were recruited through a letter of invitation under a cover  letter sent by a government agency responsible for administering health care (...) families were invited to return an enclosed stamped return postcard if they had a healthy child” |
|  | **Age** 4.14 ± 0.24 yr |
|  | **Sex** 115 boys, 85 girls |
|  | **Exclusion criteria** “Overall, the demographic profile suggests that the sample was composed of families at low risk for dysfunction.” |
| **Protocol** | ”We collected salivary cortisol samples before and after the two initial failures (easy and difficult matching tasks): one prestressor sample at the end of the settling period and five poststressor samples taken 15, 20, 25, 30, and 40 min after failure on the easy matching task.” |
| **Outcomes** | **Primary** “Characterize cortisol response and regulation associated with shame  responding in early childhood” |
|  | **Secondary** “To examine how general the relation between shame and cortisol is” |
| **Results** | The salivary cortisol level prestressor was included in the meta-analysis   \|  \| Boys \|  \|  \|  \| Girls \|  \|  \|  \| \| --- \| --- \| --- \| --- \| --- \| --- \| --- \| --- \| --- \| \| Cortisol levels (µg/dL)  Prestressor \| *n* \| M (SD) \| Min \| Max \| *n* \| M (SD) \| Min \| Max \| \| 115 \| 0.11 (0.10) \| 0.02 \| 0.77 \| 85 \| 0.10 (0.10) \| 0.02 \| 0.87 \| |

| Minkley 2012 [44] | |
| --- | --- |
| **Methods** | **Design** Non-randomized intervention study |
|  | **Setting** Bochum, Germany |
|  | **Timing** at 11.10 am, the first saliva sample was taken |
| **Participants** | ***n=*** 93 |
|  | **Subjects** “students of biology courses at secondary or comprehensive schools (*n=*13) in Bochum or nearby cities.” |
|  | **Age** 17.86 yr (± 0.096 S.E.M.) |
|  | **Sex** 56 males and 37 females |
|  | **Exclusion criteria** “smoking, over or underweight (BMI above 25 kg/m^2^ or below 18.5, respectively), use of any long-term medication or oral contraceptives, or presence of a medical condition” |
| **Protocol** | “Participants were randomly assigned to one of the four different treatment groups: the first groups was asked to deal with tasks associated with the reproduction of knowledge (…) while students assigned to the second group were asked to deal with tasks associated with transfer and problem-solving (…). The third group served as the control for the writing activity (…) The fourth group also served as a control (…)(11.10 am), the first saliva sample was taken (duration: 5 min) to identify the cortisol baseline.” |
| **Outcomes** | **Primary** “the extent to which tasks with differing cognitive demands can influence the cortisol concentration of school students” |
|  | **Secondary** “Whether their sex modulates this hormonal reaction.” |
| **Results** | I have emailed Dr. Minkley [Nina.Minkley@rub.de] with the request for sex-specific data. Her response:  Dear Bibian, you will find the new table in the attachment. I have also seen, that I have accidentally reported ng-values instead of nmol in the last table I send to you. Now I have corrected all values to nmol/L. Sorry for that!  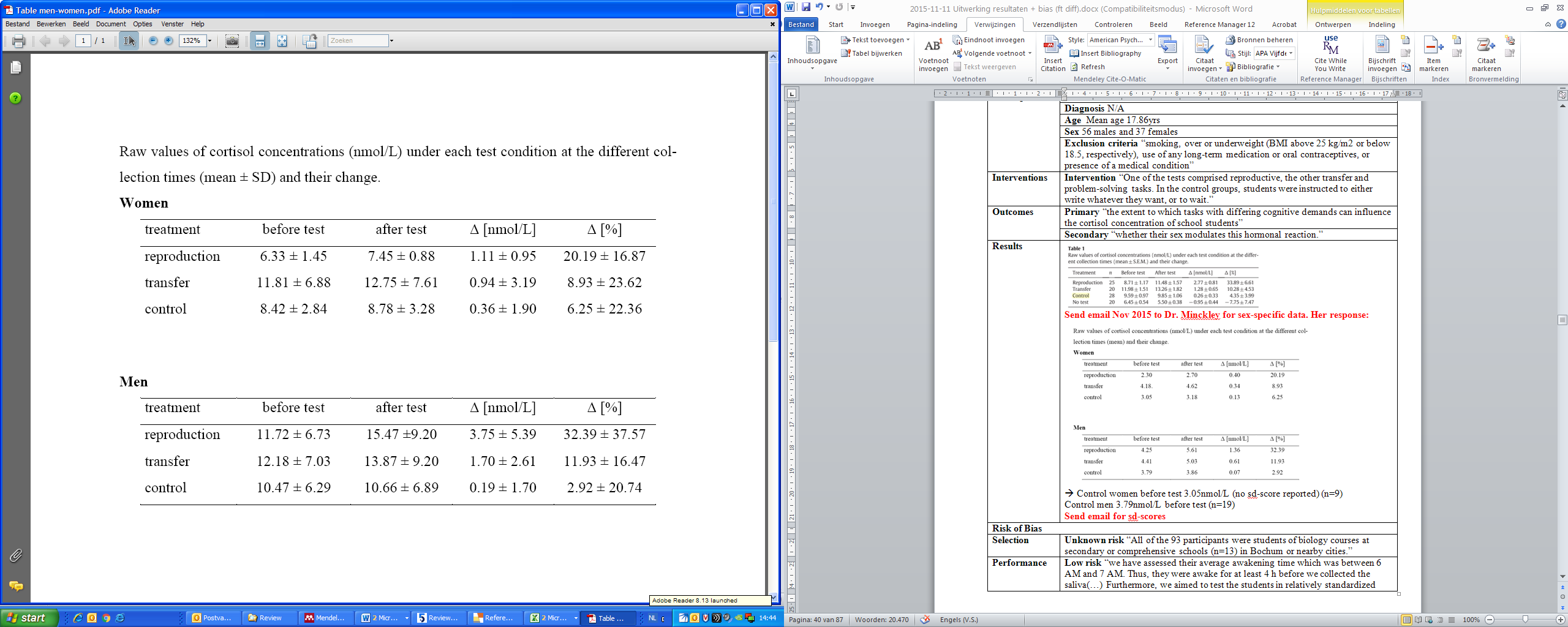 |

| **Mrug 2016** [45] | |
| --- | --- |
| **Methods** | **Design** non-randomized intervention study |
|  | **Setting** Birmingham, USA |
|  | **Timing “**between 3 and 6 pm |
| **Participants** | ***n =*** 84 |
|  | **Subjects** “The sample was socioeconomically heterogeneous, but comprised primarily low-income families; average annual family income was $20,000–$25,000 (range $5000 to $70,000–$90,000) and average parental education was some college but no degree (see Table 1 for sample characteristics). The adolescents were recruited from four public middle schools (grades 6–8 or 9) serving low income, urban communities in Birmingham, AL. Across the four schools, 83% to 87% of students were eligible for free or reduced price lunch. “ |
|  | **Age:** 13.36 ± 0.95 yr |
|  | **Sex:** 42 girls and 42 boys |
|  | **Exclusion criteria:** any psychiatric disorders (per parent report) or any medications that would affect cortisol levels (per self-report). |
| **Protocol** | “Trier Social Stress Test-Children's version (TSST-C) with saliva samples collected before and after the TSST-C (…) Whole saliva samples were collected by passive drool immediately before the TSST began (pre-test), 30min after the 15-min test began(15 min post-test), and 70min after the test began (55 min post-test)” |
| **Outcomes** | **Primary:** “The role of sleep problems and sleep duration on stress-related HPA axis reactivity among urban, low income adolescents.“ |
|  | **Secondary:** Gender differences |
| **Results** | I have emailed Dr. Mrug (sylva@uab.edu) with the request for sex-specific data. Her response:    Here are the sex-specific means and tests of sex differences. Good luck with your work!   \|  \| gender \| N \| Mean \| Std. Deviation \| Std. Error Mean \| T-test (*P*-value) \| \| --- \| --- \| --- \| --- \| --- \| --- \| --- \| \| cortl1tr \| Male \| 40 \| .1242 \| .10250 \| .01621 \| .067 \| \| Female \| 39 \| .0837 \| .09071 \| .01453 \|  \| \| cortl2tr \| Male \| 41 \| .0877 \| .05832 \| .00911 \| .120 \| \| Female \| 37 \| .1405 \| .20620 \| .03390 \|  \| \| cortl3tr \| Male \| 41 \| .0466 \| .03118 \| .00487 \| .045 \| \| Female \| 42 \| .0864 \| .12169 \| .01878 \|  \|   Cortisol was measured in µg/dL |

| Nakamura 1984 [46] | |
| --- | --- |
| **Methods** | **Design** Cross-sectional study |
|  | **Setting** Niigata, Japan |
|  | **Timing** 24h collection |
| **Participants** | ***n =***  15 subjects 5 – 10yr and 13 subjects 14 – 19yr |
|  | **Subjects** “normal men and women” |
|  | **Age** two groups: 5 – 10 and 14 – 19 yr, see table 1 |
|  | **Sex** 7 boys and 8 girls aged 5 – 10yr; 6 boys and 7 girls aged 14 – 19yr |
|  | **Exclusion criteria** not described |
| **Protocol** | 24h-urine collection, not further described |
| **Outcomes** | **Primary** “A possible difference in the physiological function of cortisol with age“ |
|  | **Secondary** Gender differences |
| **Results** | \| Age (yr) \| *n* \|  \| Mean urinary cortisol (µg/day) (SD) \| \| \| --- \| --- \| --- \| --- \| --- \| \|  \| men \| women \| men \| women \| \| 5 – 10 \| 7 \| 8 \| 14.8 (3.6) \| 9.98 (3.2) \| \| 14 – 19 \| 6 \| 7 \| 40.9 (23.0) \| 33.3 (3.1) \| |

| Ong 2004 [47] | |
| --- | --- |
| **Methods** | **Design** Longitudinal, cohort study (ALSPAC cohort) |
|  | **Setting** Bristol, United Kingdom |
|  | **Timing** 08:50 am ± 22 min |
| **Participants** | ***n =*** 770 |
|  | **Subjects** The Avon Longitudinal Study of Parents and Children (ALSPAC) is a prospective study of 14,541 pregnancies recruited from all pregnancies in three Bristol-based District Health Authorities with expected dates of delivery between April 1991 and December 1992 (...) Eight hundred fifty-one (...) children from these two subcohorts attended a research clinic and gave a fasting blood sample and had body size measurements taken at age 8 yr (...)These children did not differ from other ALSPAC children with regard to body size at birth or during childhood.” |
|  | **Age** 8.2 ± 0.1 yr |
|  | **Sex** 423 boys and 347 girls |
|  | **Exclusion criteria** Oral steroid use or when admitted that they did not fast. |
| **Protocol** | **“**A venous blood sample was collected after application of topical analgesic cream” |
| **Outcomes** | **Primary** The influence of birth weight and early postnatal weight gain on  overnight-fasting adrenal androgen and cortisol levels in 770 children from a large normal United Kingdom birth cohort at age 8 yr. |
|  | **Secondary** Gender differences |
| **Results** | \| Hormone levels at age 8 yr \| Mean or geometric mean (1SD range) \| \| \| --- \| --- \| --- \| \|  \| Boys (*n* = 423) \| Girls (*n* = 347) \| \| Cortisol (nmol/L) \| 210 (160 – 276) \| 215 (157 – 295) \|   Based on the 1SD range the average SD was calculated: (upper SD-lower SD)/2 |

#

| Osika 2007 [48] | |
| --- | --- |
| **Methods** | **Design** Prospective, observational study |
|  | **Setting** Gothenburg, Sweden |
|  | **Timing** morning |
| **Participants** | ***n =*** 84 |
|  | **Subjects** “The following inclusion criteria were applied:  - ability to read and understand the Swedish language  - not using any medication” |
|  | **Age** 9.9 ± 0.6 yr |
|  | **Sex** 50 girls, 34 boys |
|  | **Exclusion criteria “**Presence of any known chronic disease (information supplied by parents, the pupil, school nurses, or teachers)” |
| **Protocol** | “During an ordinary school week The first sample (SC1) was collected immediately upon waking in the morning, irrespective of time. The next sample (SC2) was collected 15 min later (...) The third sample (SC3) was obtained at (...) 9 am.(…) The fourth sample (SC4) was collected approximately 15 min before lunch (...) (11 am). The fifth and final sample (SC5) was collected at bedtime.” |
| **Outcomes** | **Primary** “Our aim was to assess the magnitude of stress in children of both sexes “ |
|  | **Secondary** “To validate this questionnaire against established psychometric measures for closely related states such as depression, anxiety, anger, disruptive behavior, and self-perception (Beck Youth Inventories)” |
| **Results** | Our data showed a saliva cortisol mean level of 5.50 nmol/L (range 2.5–  15.8) in the 48 girls with adequate samples of SC3 and 5.34 nmol/L (range 3.2–11.6) among the 31 boys.  The cortisol levels at SC3 were included in the meta-analysis, because these are the only levels that were reported sex-specifically in absolute numbers.  Mean and SD levels were calculated assuming that 95% of values will lie within 2×SD either side of the mean. The SD may therefore be estimated to be approximately one quarter of the typical range of data values.” [5]  In other words SD = (Maximum – Minimum)/4.  Girls: 5.50 ± 3.33nmol/L  Boys: 5.34 ± 2.1nmol/L |

| Pérez-Edgar 2008 [49] | |
| --- | --- |
| **Methods** | **Design** Longitudinal, cohort study |
|  | **Setting** Fairfax, United States |
|  | **Timing** 8 am ± 0:48h |
| **Participants** | ***n =*** 111 |
|  | **Subjects** “Of the 433 infants screened, 153 were selected for inclusion in the longitudinal studies. The families were Caucasian and of middle-class background, living in the greater Washington, DC area. Approximately 68% of the mothers and 72% of the fathers were college educated” |
|  | **Age** 4 yr |
|  | **Sex** 51 males and 60 females |
|  | **Exclusion criteria** No pre- or perinatal complications [50] |
| **Protocol** | A single composite measure of mean morning cortisol level (expressed in µg/dL) was computed by averaging across all useable morning samples. The average collection time was 7:53 am and reliable data were available for 111 4-year-olds (51 males). |
| **Outcomes** | **Primary** “Negative temperament in infancy would be associated with increased levels of withdrawal at age 4, particularly coupled with high levels of concurrent basal cortisol” |
|  | **Secondary** “This relation would be particularly strong in boys” |
| **Results** | \|  \| Overall \| Boys \| Girls \| \| --- \| --- \| --- \| --- \| \| Basal cortisol (µg/dL) \| 0.41 (0.18) \| 0.43 (0.17) \| 0.39 (0.18) \| |

| **Portnoy 2015** [51] | |
| --- | --- |
| **Methods** | **Design** non-randomized intervention study (the Healthy Brains and Behavior study) |
|  | **Setting** Philadelphia, USA |
|  | **Timing** 9.18am |
| **Participants** | ***n =*** 353 |
|  | **Subjects** “ The sample for this study consisted of 11 and 12-year old boys and girls living in Philadelphia County, PA or suburbs of Philadelphia. Within the study area, fliers soliciting enrollment were placed in recreation centers, libraries, health clinics, and other community centers. Targeted mailings were also sent  to parents of 11–12 year old children living in the geographic catchment area. “ |
|  | **Age** 11.92 ± 0.59 yr |
|  | **Sex** 178 females and 175 boys |
|  | **Exclusion criteria** “A diagnosed psychotic disorder, mental retardation, or a pervasive developmental disorder” |
| **Protocol** | “A morning saliva sample was collected at an average time of 9:18 am. Between sample collections, subjects completed behavioral questionnaires. In the afternoon, four saliva samples were collected to assess cortisol reactivity to the stressor at the following times: (1) Immediately prior to the laboratory tasks (mean time = 12:36 pm), (2) 5 min after the end of the stress task (mean time = 1:27 pm), (3) 20 min after the end of the stress task (mean time = 1:42 pm),and (4) 40 min after the end of the stress task (mean time = 2:02pm)” |
| **Outcomes** | **Primary** “ whether 2D:4D inter-acts with cortisol and adolescent testosterone level to predict externalizing behavior in a sample of young adolescents.” |
|  | **Secondary** Gender differences and influence of aggressive and non-aggressive forms of antisocial behaviour. |
| **Results** | I have emailed Dr. Portnoy (jill@sas.upenn.edu) for gender-specific absolute cortisol levels. She replied:  Good luck with your meta-analysis. I attached SPSS output with the information you requested.  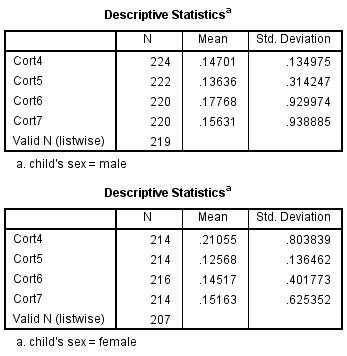  Cortisol was measured in µg/dL |

| Reynolds 2013 [52] | |
| --- | --- |
| **Methods** | **Design** Longitudinal, cohort study (Raine cohort) |
|  | **Setting** Edinburgh, United Kingdom |
|  | **Timing** Fasting morning blood and saliva samples |
| **Participants** | ***n=*** 1258 |
|  | **Subjects** “All adolescent participants were part of The Western Australian Pregnancy (Raine) cohort, a prospective population-based pregnancy cohort study of 2868 live births occurring between 1989 and 1991“ |
|  | **Age** 16.6 ± 0.5 yr |
|  | **Sex** 653 males, 605 females |
|  | **Exclusion criteria** pregnancy |
| **Protocol** | “A fasting morning EDTA-blood sample was collected under non-arousing circumstances during a home visit by a Raine Study phlebotomist. Participants refrained from eating or drinking anything other than water from 2200 h the night before blood collection. The date and time of blood collection before 1000h in the morning was documented. Saliva was self-collected by 1121 Raine participants using Salivette saliva collection tubes (Sarstedt, Germany) 15 min after spontaneous awakening on 3 consecutive weekdays (S1-3). The majority of participants (75.1%) provided three samples collected on consecutive days just prior to the day of blood collection. The participants recorded the date and time of collection before 10 am in the morning on sample tubes” |
| **Outcomes** | **Primary** to investigate basal HPA-activity in a normal population in late  adolescence, |
|  | **Secondary** Gender-specific stress-system profiles |
| **Results** | \|  \| Participants \| *n* \| Mean (SD) \| Range Min – Max \| \| --- \| --- \| --- \| --- \| --- \| \| Total plasma  cortisol (µg/dL) \| All \| 1258 \| 22.25 (8.35) \| 1.33 – 59.97 \| \| Males \| 653 \| 19.98 (5.60) \| 1.33 – 42.72 \| \| Females \| 605 \| 24.71 (9.99) \| 1.65 – 59.97 \| \| Females (no  known OC use) \| 401 \| 21.42 (7.35) \| 1.65 – 54.32 \| \| Salivary free  cortisol (µg/dL) \| All \| 1121 \| 0.99 (0.59) \| 0.11 – 5.33 \| \| Males \| 573 \| 0.94 (0.51) \| 0.11 – 3.38 \| \| Females \| 548 \| 1.04 (0.65) \| 0.12 – 5.33 \| \| Females (no known OC use) \| 362 \| 1.07 (0.69) \| 0.14 – 5.37 \|   The Female group with no known OC use was included in the meta-analysis. |

#

| Ross 1986 [53] | |
| --- | --- |
| **Methods** | **Design** Non-randomized intervention study |
|  | **Setting** Bethesda, USA |
|  | **Timing** between 7 and 8 pm |
|  | **End point measurements** “ACTH and cortisol responses to CRH” |
| **Participants** | ***n =*** 47 |
|  | **Subjects** “normal children” |
|  | **Age** aged 6 – 15yr |
|  | **Sex** 23 boys and 24 girls |
|  | **Exclusion criteria** not described |
| **Protocol** | **Protocol** “CRH stimulated release of immunoreactive ACTH and cortisol (...) blood was drawn at –15, 0, 30, 60, 90, 120 and 180 min after CRH injection for measurement of ACTH, cortisol, and CRH” |
| **Outcomes** | **Primary** “Age-related changes in the ACTH and cortisol responses to CRH during childhood.” |
|  | **Secondary** “The relationship between cortisol-binding globulin (CBG) and chronological age” |
| **Results** | 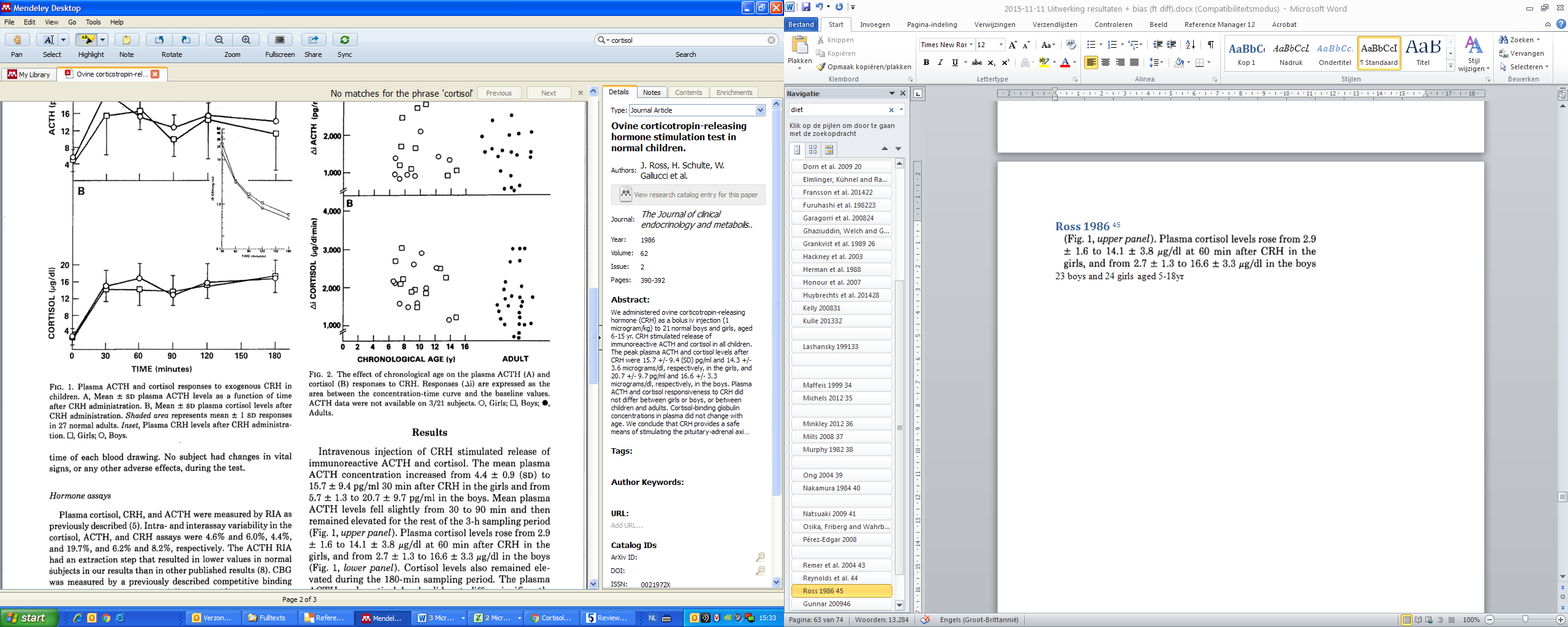  “Plasma cortisol levels rose from 2.9 ± 1.6 to 14.1 ± 3.8 μg/dL at 60 min after CRH in the girls, and from 2.7 ± 1.3 to 16.6 ± 3.3 μg/dL in the boys ” |

| Soriano-Rodriguez 2010 [54] | |
| --- | --- |
| **Methods** | **Design** Prospective, observational study |
|  | **Setting** Figueres, Spain |
|  | **Timing** between 8 and 9 am under fasting conditions |
| **Participants** | ***n=*** 223 |
|  | **Subjects** “A sample of healthy prepubertal children (…) attending primary care clinics (...) Inclusion criteria were 1) age between 5 and 9 yr and 2) absence of puberty, as based on the standards by Marshall and Tanner.” |
|  | **Age** 6.8 ± 0.1 y |
|  | **Sex** 106 boys and 117 girls |
|  | **Exclusion criteria** “Major congenital abnormalities, abnormal blood counts, liver or kidney or thyroid functions, evidence of chronic illness or chronic use of medication, and acute illness or medication use within the previous month” |
| **Protocol** | “Clinical examination was performed in the morning, in the fasting state followed by venous blood sampling. A local anesthetic cream was used to minimize the discomfort of venipuncture.” |
| **Outcomes** | **Primary** “Cross-sectional associations between morning serum cortisol and vascular risk markers |
|  | **Secondary** Gender interactions |
| **Results** | \|  \| Boys (*n* = 106) \| Girls (*n* =117) \| \| --- \| --- \| --- \| \| Cortisol (mg/dL) mean ± SEM \| 16.0 ± 0.5 \| 14.8 ± 0.5 \|   We assumed that the wrong unit of measurement was reported here, and emailed Dr. Soriano-Rodriguez with the question if it could have been µg/dL. She did not reply. Since cortisol levels > 400,000 nmol/L would be impossible, we assumed that these cortisol levels were reported in µg/dL and converted them subsequently: Boys: 441.4 ± 13.8 nmol/L Girls 408.3 ± 13.8 nmol/L |

| Stroud 2011 [55] | |
| --- | --- |
| **Methods** | **Design** non-randomized intervention study |
|  | **Setting** Providence, USA |
|  | **Timing** 4pm |
| **Participants** | ***n=*** 68 |
|  | **Subjects** “Participants were 68 carefully screened controls (41% girls, 59% boys) recruited over three phases of a multi-project study of neurobehavioral characteristics of pediatric affective disorders who completed at least one baseline CRH challenge with available cortisol and Tanner stage data.” |
|  | **Age** 10.5 ± 1.7 yr |
|  | **Sex** 30 girls and 39 boys |
|  | **Exclusion criteria** Personal or family history of psychiatric disorder with high familial risk for depression. “Additional exclusion criteria were: (a) use of medications except acetaminophen within two weeks of the protocol, (b) obesity, (c) height or weight below 3^rd^ percentile, and (d) IQ below 70 or learning disability” |
| **Protocol** | **“**The CRH infusion protocol began at 4:00 pm (Phases 2 and 3) or 5:00 pm (Phase 1) on the second laboratory day. These times were chosen as points when the HPA axis is believed to be relatively quiescent (Puig-Antich et al., 1989). The CRH challenge protocol included 30—40 min pre-infusion baseline, followed by 1 mg/kg human CRH (hCRH) administered as an intravenous infusion over two minutes, then 90—180 min of recovery. Nine to ten plasma cortisol samples were collected over the course of the CRH challenge protocol. For phase one of the study, basal samples were collected at -30, -15, and 0 min, with 0 as the time of CRH infusion. “ |
| **Outcomes** | **Primary** “To characterize the joint effects of gender (G) and pubertal stage (Tanner) on cortisol response to CRH challenge.” |
|  | **Secondary** NA |
| **Results** | In the meta-analysis Tanner I/II girls and boys who are approximately 10yr of age, were included.   \| Point estimate (95% CI) \|  \| \| --- \| --- \| \| Baseline cortisol (µg/dL) \| Tanner I/II \| \| Girls (*n*= 30) \| 5.31 (4.89; 5.74) \| \| Boys (*n*= 39) \| 5.96 (5.54; 6.38) \|   95%CIs were converted to SDs: upper limit – lower limit /3.92 [5]  Boys: 164.4 ± 5.90 nmol/L Females: 146.5 ± 5.99 nmol/L |

| Stupnicki 1995 [56] | |
| --- | --- |
| **Methods** | **Design** Prospective, observational study |
|  | **Setting** Warsaw, Poland |
|  | **Timing** “The exercise started at 0900 hours and was preceded by a light breakfast at 0730 hours (…) Blood was withdrawn from an antecubital vein just before and 2 min after the end of the exercise.” |
| **Participants** | ***n=*** Junior rowers: 29 (14 male, 15 female) |
|  | **Subjects** Junior rowers |
|  | **Age** males: 17.3 ± 0.8 yr, females: 16.4 ± 0.6 yr |
|  | **Sex** 14 male, 15 female |
|  | **Exclusion criteria** Not described |
| **Protocol** | “Graded exercise, performed on a rowing ergometer (Concept II), consisting of three periods (5 rain each) of exercise separated by 5-min intermissions. Consecutive periods were of increasing intensity, 50%, 70%, and 85% of individual mean power output recorded previously in a laboratory exercise simulating a 2-km run” |
| **Outcomes** | **Primary** Whether exercise performance is dependent on the pre-exercise blood concentration of cortisol |
|  | **Secondary** Relationships between postexercise values of blood lactate concentration, pH, oxygen uptake, heart rate, power output, and the pre- and postexercise blood cortisol concentrations |
| **Results** | \|  \| Junior male rowers \| \| Junior female rowers \| \| \| --- \| --- \| --- \| --- \| --- \| \|  \| Mean \| SD \| Mean \| SD \| \| Cortisol (nmol/L) pre-exercise \| 347 \| 130 \| 397 \| 125 \| |

| **Susman 1991** [57] | |
| --- | --- |
| **Methods** | **Design** Longitudinal, cohort study |
|  | **Setting** Pennsylvania, USA |
|  | **Timing** 8 am |
| **Participants** | ***n =*** 108 |
|  | **Subjects** “The participants were 10-15-year old boys (N = 56) and 9-14-year-old  girls (N = 52) and their parents. The adolescents were recruited into the study  until there was approximately an equal number of boys and girls in each of  the five stages of pubertal development (Tanner criteria; Marshall and Tanner, 1969, 1970). The adolescents and their parents were part of a larger longitudinal study of developmental changes in hormones and physical stature and psychological processes during early adolescence (Nottelmann, Susman,  Dorn et al., 1987). “ |
|  | **Age** 9 – 14yr old; mean age boys 12.7 yr and girls 12 yr [58] |
|  | **Sex** 56 boys and 52 girls |
|  | **Exclusion criteria** not described in this publication, neither in [58] |
| **Protocol** | “At each time of measurement, blood was drawn at 0, 20, and 40 minutes  for analysis (...) The range in reliabilities (correlations) among the three samples at the three times of measurement were: (…) cortisol, .51-.97. The mean of the three blood samples is used in all analyses reported here. Blood was drawn at 8:00 am to control for diurnal variations in hormone levels.” |
| **Outcomes** | **Primary** “Relations among negative affect and hormones of gonadal and adrenal origin in young adolescents, at three times of measurement, over a one-year period” |
|  | **Secondary** “Stability of negative affect” |
| **Results** | T1 was included in the meta-analysis.   \| T1 \|  \| Mean \| SD \| \| --- \| --- \| --- \| --- \| \| Cortisol (µg/dL) \| Males \| 13.22 \| 5.42 \| \|  \| Females \| 12.48 \| 5.39 \| |

| Syme 2008 [59] | |
| --- | --- |
| **Methods** | **Design** Prospective, observational study |
|  | **Setting** Quebec, Canada |
|  | **Timing** “A fasting blood sample was drawn between 8 am and 9 am” |
| **Participants** | ***n =***  324, from which 159 with low intra-abdominal fat (low IAF) |
|  | **Subjects** “Adolescents, aged 12 to 18 years, were recruited in a remote, French-Canadian population as part of the Saguenay Youth Study; all subjects were white. This is an ongoing, cross-sectional, family-based (adolescent sibships) investigation of the long-term consequences of prenatal exposure to maternal cigarette smoking (PEMCS) on cardiovascular and metabolic health and on the brain and behavior in adolescence” |
|  | **Age** Low IAF Males 14.4 ± 1.7 yr; Low IAF females 14.4 ± 1.9 yr |
|  | **Sex** 77 Low IAF males and 82 Low IAF females |
|  | **Exclusion criteria** “1) Positive history of alcohol abuse during pregnancy; (2) Positive medical history for meningitis, malignancy, and heart disease requiring heart surgery; (3) Severe mental illness (e.g. autism, schizophrenia) or mental retardation (IQ < 70); and (4) MRI contraindications.”[60] |
| **Protocol** | “A fasting blood sample was drawn between 8 am and 9 am (...) Subjects were divided into low and high IAF groups based on the sex-specific median of IAF. The median was used because, to our knowledge, there are no established cutoffs for high or low IAF” |
| **Outcomes** | **Primary** “Primary outcome measures were blood pressure (BP) and fasting serum glucose, insulin, lipids, and C-reactive protein levels.“ |
|  | **Secondary** “Secondary mechanistic measures were cardiovascular variability indexes of autonomic nervous system function, pubertal development, and serum levels of cortisol, leptin, and sex hormones.” |
| **Results** | \|  \| Males \| Females \| \| --- \| --- \| --- \| \|  \| Low IAF (*n* = 77) \| Low IAF (*n* = 82) \| \| Cortisol (µg/dL) Mean (SD) \| 19.8 (5.1) \| 25.9 (11.1) \| |

| Tennes 1973 [61] | |
| --- | --- |
| **Methods** | **Design** Prospective, observational study |
|  | **Setting** Colorado, USA |
|  | **Timing** 7 – 8 am |
| **Participants** | ***n =*** 40 |
|  | **Subjects** “The population consisted of 40 infants in the full-term nursery or rooming-in unit at Colorado General Hospital.” |
|  | **Age** the 3^rd^ day of life |
|  | **Sex** 23 boys and 17 girls |
|  | **Exclusion criteria** not described |
| **Protocol** | Neonatal phenylketonuria (PKU) screening: “Two heparinized capillary tubes of blood were obtained from the infant by the technician taking blood for PKU analysis.” |
| **Outcomes** | **Primary** “The relationship between cortisol and sex, birthweight, Apgar score, circumcision, and behavioural state” |
|  | **Secondary** “The second part of the present study attempted to examine the relationship between these chronic periods of irritability and plasma cortisol levels in the infant during the first three months of life.” |
| **Results** | “The mean of the cortisol determinations for the 23 boys, 15.8 µg/100 mL was not significantly different from the mean of the 17 girls, 14.1 µg/100 mL” |

| Törnhage 2002 [62] | |
| --- | --- |
| **Methods** | **Design** Cross-sectional study |
|  | **Setting** Karlstad, Sweden |
|  | **Timing** 8 – 9 am |
| **Participants** | ***n=*** 386 |
|  | **Subjects** “Healthy schoolchildren” |
|  | **Age** 7 – 15 years (see table 1) |
|  | **Sex** 210 boys and 176 girls |
|  | **Exclusion criteria** not described |
| **Protocol** | **Protocol** “Saliva collection was performed in the classroom between 08.00 and 09.00h after a period of rest.” |
| **Outcomes** | **Primary** “To estimate salivary cortisol concentrations in healthy school-aged children” |
|  | **Secondary** “Relate the concentrations to age, sex, stage of puberty and adult values” |
| **Results** | “Boys and girls had nearly the same median concentrations, 8.8 versus 8.6 nmol/L, but girls had a higher maximal level, 53.9 compared to 33.2 nmol/L in boys. The median concentration was lower in 7-9 year-old children, 7.2 in boys and 5.7 nmol/L in girls, compared to 10-12 year-old children, 11.5 in boys and 10.9nmol/L in girls”  Ranges were converted to SDs: upper limit – lower limit /4 [5]  In the meta-analysis the group of children <8yr and the largest group of children >8yr (see table 1 in the article) were included with a mean age of 7.1 and 7.4 yr, and 10.2 and 10.3 yr, for boys and girls respectively  🡪 girls ±7 yr median (range): 5.2 (2.5 – 10.0) 🡪 5.2 ± 1.88 nmol/L (*n* = 11)  🡪 boys ±7 yr median (range): 10.5 (4.9 – 25.8) 🡪 10.5 ± 5.2 nmol/L (*n* = 19)  🡪 girls ±10 yr median (range): 8.9 (1.8 – 53.9) 🡪 8.9 ± 13.03 nmol/L (*n* = 31)  🡪 boys ±10 yr median (range): 11.0 (4.6 – 20.9) 🡪 11.0 ± 4.08 nmol/L (*n* = 28) |

| Tout 1998 [63] | |
| --- | --- |
| **Methods** | **Design** Prospective, observational study |
|  | **Setting** Minnesota, USA |
|  | **Timing** approximately 10:30 am |
| **Participants** | ***n =*** 75 |
|  | **Subjects** “Preschool children were recruited from six classes at two urban child care centers, one university based (four classes, n = 81) and one community based (two classes, n = 42). While these two centers represented samples of convenience, they were selected, in part, because they allowed us to examine an ethnic mix of children that was broadly representative of the metropolitan area.” |
|  | **Age** mean age 4.3yr |
|  | **Sex** 37 girls and 38 boys |
|  | **Exclusion criteria** insufficient days of cortisol sampling |
| **Protocol** | “For a period of approximately 30 days, we visited each center twice daily to collect saliva samples and behavioral observations. The behavioral observations  were conducted in both the morning and afternoon at each center. The saliva samples for cortisol were obtained at approximately 10:30 am and 3:00 pm on  each assessment day.” |
| **Outcomes** | **Primary** “Relations between social behavior and daily patterns of a stress-sensitive hormone production were examined in preschool children (n = 75) attending center-based child care.” |
|  | **Secondary** Gender differences |
| **Results** | The morning samples were included in the meta-analysis.   \| Cortisol measures in µg/dL \| \| \| \| \| \| \| \| --- \| --- \| --- \| --- \| --- \| --- \| --- \| \|  \| Total \| \| Girls \| \| Boys \| \| \|  \| Mean \| SD \| Mean \| SD \| Mean \| SD \| \| Morning median \| 0.17 \| 0.06 \| 0.16 \| 0.05 \| 0.18 \| 0.07 \| |

| Tsvetkova 1977 [64] | |
| --- | --- |
| **Methods** | **Design** Non-randomized intervention study |
|  | **Setting** Sofia, Bulgaria |
|  | **Timing** 8 am, before breakfast |
| **Participants** | ***n =*** 31 |
|  | **Subjects** “healthy children” |
|  | **Age** 4 – 14 years |
|  | **Sex** 16 boys and 15 girls |
|  | **Exclusion criteria** not described |
| **Protocol** | “Adrenocortical function was tested before and 2 hours after 0.5 mg tetracosactrin given intramuscularly” |
| **Outcomes** | **Primary** “To assess the effect of ACTH on the adrenal cortex when used as a therapeutic or diagnostic agent” |
|  | **Secondary** The effect of age and sex |
| **Results** | \| Mean values of plasma cortisol (µg/100ml) at baseline \| Boys \| Girls \| \| --- \| --- \| --- \| \| Age 4 to 10yr \| *n* = 9 \| *n* = 8 \| \| Mean \| 18.8 \| 18.5 \| \| SD \| 7.0 \| 5.7 \| \| SE \| 2.3 \| 1.9 \| \| Age 11 to 14yr \| *n* = 7 \| *n* = 7 \| \| Mean \| 15.4 \| 14.8 \| \| SD \| 6.2 \| 5.8 \| \| SE \| 2.4 \| 2.2 \| |

| **Turan 2015** [65] | |
| --- | --- |
| **Methods** | **Design:** Non-randomized intervention study |
|  | **Setting:** Birmingham, USA |
|  | **Timing: “**Participants were scheduled between noon and sundown to minimize the effects of circadian rhythms on hormone levels.” |
| **Participants** | ***n =*** 153 |
|  | **Subjects:** “To control for race/ethnicity, only children whose caregivers identified them as Black (*n* = 43), non-Hispanic White (*n* = 50), and Hispanic (*n* = 60) were included in the analyses, as these reflect the most sizable racial/ethnic subgroups. Participants were primarily recruited from local public schools in an urban Southwestern community.” |
|  | **Age:** 9.38 ± 0.62 yr |
|  | **Sex:** 79 boys and 74 girls |
|  | **Exclusion criteria:** “Psychotic disorders, neurodevelopmental disorders, and mental retardation in the child.” |
| **Protocol** | “Children provided their first (baseline) saliva sample 30 min after arrival at the lab (just before the start of the stress task). Additional (post-stressor) samples were collected 20 min, 40 min, and 60 min after the start of the stress task (…) Four saliva samples per participant (approximately 1–2 ml per sample) were obtained by having participants drool passively into test tubes using straws (without the use of gum to stimulate saliva).”  The stress task was the TSST. |
| **Outcomes** | **Primary:** “A coordinated response in cortisol and testosterone (i.e., coupling of  cortisol and testosterone responses) during a social evaluative situation.” |
|  | **Secondary:** Influence of gender and reported anxiety |
| **Results** | \| Cortisol (nmol/L) \| Boys \|  \| Girls \|  \| \| --- \| --- \| --- \| --- \| --- \| \|  \| Mean \| SD \| Mean \| SD \| \| Baseline \| 5.197 \| 3.239 \| 5.634 \| 10.792 \| |

| Tzortzi 2009 [66] | |
| --- | --- |
| **Methods** | **Design** Prospective, observational study |
|  | **Setting** Regensburg, Germany |
|  | **Timing** “They were instructed to start sampling immediately after waking up: during the first three hours, one sample each 20 minutes, then one sample  each hour until bedtime.” |
| **Participants** | ***n =*** 21 |
|  | **Subjects** healthy school children |
|  | **Age** males (10y 3m – 13y 7m); females (age range: 10y 3m – 13y 3m) |
|  | **Sex** 11 males and 10 females |
|  | **Exclusion criteria** “Chronic diseases, medication intake, hormone therapy, allergies, or any invasive medical procedure” |
| **Protocol** | “Each volunteer collected 20 saliva samples during one day at defined times starting immediately after waking up and ending at night.” |
| **Outcomes** | **Primary** “To establish detailed levels of daily salivary cortisol secretion in  school children in order to determine the precise time range of its lowest concentration and individual variability.” |
|  | **Secondary** “A baseline for normalised saliva cortisol concentration in order to perform further studies in this particular age group” |
| **Results** | \|  \|  \| *n* \| Mean \| SD \| Min \| Max \| Median \| \| --- \| --- \| --- \| --- \| --- \| --- \| --- \| --- \| \| Cortisol (nmol/L) \| Male \| 11 \| 9.05 \| 3.63 \| 4.4 \| 15.6 \| 8.52 \| \|  \| Female \| 10 \| 10.24 \| 4.86 \| 1.3 \| 20.6 \| 9.45 \| \|  \| Total \| 21 \| 9.62 \| 4.19 \| 1.3 \| 20.6 \| 9.03 \| |

| Vaindirlis 2000 [67] | |
| --- | --- |
| **Methods** | **Design** Case-control study |
|  | **Setting** Athens, Greece |
|  | **Timing “**24-hour urine specimen (…) There were two separate collections of 12-hour duration, one for day (7:00 am to 7:00 pm) and one for night (7:00 pm to 7:00 am).” |
| **Participants** | ***n=*** 22 controls |
|  | **Subjects** “Control subjects were selected for age, sex, and pubertal stage from the healthy siblings of patients with insulin-dependent diabetes mellitus (…) The control subjects had no clinical or biochemical evidence of diabetes.” |
|  | **Age** 13 ± 3.5 years |
|  | **Sex** 12 males, 10 females |
|  | **Exclusion criteria** any doubts in regard to information about urine collection |
| **Protocol** | Urine specimen, collected at home the preceding 24h |
| **Outcomes** | **Primary** “Is WCH at this developmental stage an innocent phenomenon or a prelude to future permanent adult hypertension? “ |
|  | **Secondary** “Are there any vascular abnormalities (general or renal) in adolescents? |
| **Results** | \|  \| Male control subjects \| Female control subjects \| \| --- \| --- \| --- \| \| *n* \| 12 \| 10 \| \| Total urinary cortisol (µg/24h) \| 94 ± 32 \| 69 ± 33 \| |

| West 2010 [68] | |
| --- | --- |
| **Methods** | **Design** Prospective, observational study (the PaLS study) |
|  | **Setting** Glasgow, United Kingdom |
|  | **Timing** Approximately 0900 h, duration 45–55 min |
| **Participants** | ***n=*** 2824 |
|  | **Subjects** “15-year-olds in 2006 in their final year of statutory education (Scottish S4) in 22 mainstream secondary schools situated in and around Glasgow” |
|  | **Age** 15.4 ± 0.4 yr |
|  | **Sex** 1418 males, 1406 females |
|  | **Exclusion criteria** “Extreme cases, presumed to be contaminated by blood, were excluded from analysis” |
| **Protocol** | “Briefly, participants were provided with two pre-labeled Salivettes and instructed not to eat or drink during the session, and to remove chewing gum. Five minutes into questionnaire completion (T1, mean, 0917 h), the whole group was instructed to remove a cottonwool swab from the Salivette, chew on it for 2 min and replace it in the Salivette for collection by the survey team. The process was repeated half an hour later (T2) after all participants had been interviewed and had physical measures taken” |
| **Outcomes** | **Primary** “Assessing the relative importance of school-based peer hierarchies and family socioeconomic status for psychoneuroendocrine response, here represented by cortisol ” |
|  | **Secondary** To examine differences in respect of factors previously found to be associated with salivary cortisol measures. |
| **Results** | \|  \| Males \| Females \| Total \| \| --- \| --- \| --- \| --- \| \| T1 cortisol (log) \| *n* = 1418 \| *n* = 1406 \| *n* = 2824 \| \| continuous: mean (SD) \| 1.027 (0.007) \| 1.067 (0.008) \| 1.047 (0.007) \|   Logarithmic data were converted to log10 cortisol concentrations. |

| Wudy 2007 [69] | |
| --- | --- |
| **Methods** | **Design** Cross-sectional study (DONALD study) |
|  | **Setting** Dortmund, Germany |
|  | **Timing** 24h-urine |
| **Participants** | ***n=*** 400 |
|  | **Subjects** “Healthy children and adolescents (200 boys, 200 girls aged 3–18 yr) participating in the DONALD study”[70] |
|  | **Age** 3 – 18 yr |
|  | **Sex** 200 boys, 200 girls |
|  | **Exclusion criteria** “Eligible are healthy (no prevalent diseases affecting growth and/or diet) German babies (age 3–6 month) whose mothers and/or fathers are willing to participate in a long-term study and of whom at least one parent has sufficient knowledge of the German language.” |
| **Protocol** | **Protocol “**Subjects and parents received instruction and written guidance to  ensure compliance in the 24-h urine collection, which was performed at home by each participant. All micturitions were stored immediately in preservative-free, Extran-cleaned (Extran, MA03, Merck, Darmstadt, Germany), 1-liter plastic containers at temperatures between -12 and -18 C before transfer to the research institute organized by a dietitian visiting the families and discussing collection completeness in detail” [71] |
| **Outcomes** | **Primary** “To examine the dynamics in cortisol secretion during growth” |
|  | **Secondary** “To test the hypothesis that the higher cortisol secretion in men than in women develops during puberty” |
| **Results** | The Fcomb µg/day data were included in the meta-analysis.   \| Urinary 24h-excretion rates of cortisol (absolute) according to age and sex \| \| \| \| --- \| --- \| --- \| \|  \| Boys \| Girls \| \| Age, yr \| F_comb_ µg/day \| F_comb_ µg/day \| \| 3–4 \| 20.5 ± 6.63 \| 21.2 ± 6.49 \| \| 5–6 \| 30.5 ± 10.2 \| 25.4 ± 7.52 \| \| 7–8 \| 36.9 ± 12.9 \| 31.9 ± 10.9 \| \| 9–10 \| 35.5 ± 9.26 \| 34.9 ± 12.5 \| \| 11–12 \| 54.1 ± 13.8 \| 43.6 ± 12.2 \| \| 13–14 \| 53.2 ± 21.4 \| 49.5 ± 15.1 \| \| 15–16 \| 68.5 ± 24.0 \| 65.1 ± 22.0 \| \| 17–18 \| 75.0 ± 25.3 \| 81.1 ± 32.3 \| |

| **Yu 2009** [72] | |
| --- | --- |
| **Methods** | **Design** Case-control study |
|  | **Setting** Hubei, China |
|  | **Timing** 1:30-2:30 pm |
| **Participants** | ***n=*** 20 |
|  | **Subjects** Students |
|  | **Age** 12.6 ± 1.8 yr |
|  | **Sex** 10 males and 10 females |
|  | **Exclusion criteria** “The students who were affected currently, or had a past history of endocrine or metabolic disorders, physical organic diseases, immune diseases, cerebral trauma, obesity or recent weight loss, drug, alcohol or tobacco addiction,  psychiatric and personality disorders, were excluded” |
| **Protocol** | “A 5.0 mL saliva sample was collected from each individual at the same time point during school day (1:30-2:30 pm). Saliva specimens were collected from all female students during their follicular phase of menstrual cycles.” |
| **Outcomes** | **Primary** “To explore the role of hormone levels in saliva, including cortisol (CORT), testosterone (T), prolactin (PRL) and growth hormone (GH), in formation of aggressive behaviors among 20 aggressive students and 20 non-aggressive control  students” |
|  | **Secondary** “To identify the possible biological profiles that might be responsible for adolescent aggressive behaviors.” |
| **Results** | \| Salivary levels of cortisol in non-aggressive students (mean ± SD, pg/mL) \| \| \| \| --- \| --- \| --- \| \|  \| Males, *n* = 10 \| Females, *n* = 10 \| \| Cortisol \| 22.53 ± 3.34 \| 17.51 ± 7.40 \| |

**References of Additional File 3**

1. Alghadir A, Gabr S, Al-Eisa E. Effects of Physical Activity on Trace Elements and Depression Related Biomarkers in Children and Adolescents. Biol Trace Elem Res. 2016;172:299–306.

2. Allen LB, Lu Q, Tsao JCI, Worthman CM, Zeltzer LK. Sex differences in the association between cortisol concentrations and laboratory pain responses in healthy children. Gend Med. 2009;6 Suppl 2:193–207.

3. Apter D, Pakarinen A, Hammond GL, Vihko R. Adrenocortical function in puberty. serum ACTH, cortisol and dehydroepiandrosterone in girls and boys. Acta Paediatr Scand. 1979;68:599–604.

4. Apter D, Pakarinen A, Vihko R. Serum prolactin, FSH and LH during puberty in girls and boys. Acta Paediatr Scand. 1978;67:417–23.

5. Higgins J, Green S. Cochrane Handbook for Systematic Reviews of Interventions. The Cochrane Collaboration. 2011 www.cochrane-handbook.org. Version 5.1.0

6. Azurmendi A, Pascual-Sagastizabal E, Vergara A, Munoz J, Braza P, Carreras R, et al. Developmental Trajectories of Aggressive Behavior in Children from Ages 8 to 10: The Role of Sex and Hormones. Amnerican J Hum Biol. 2016;28:90–7.

7. Bailey D, Colantonio D, Kyriakopoulou L, Cohen AH, Chan MK, Armbruster , et al. Marked biological variance in endocrine and biochemical markers in childhood: Establishment of pediatric reference intervals using healthy community children from the CALIPER cohort. Clin Chem. 2013;59:1393–403.

8. Colantonio DA Kyriakopoulou L, Chan MK, Daly CH, Brinc D, Venner AA, et al. Closing the gaps in pediatric laboratory reference intervals: A caliper database of 40 biochemical markers in a healthy and multiethnic population of children. Clin Chem. 2012;58:854–68.

9. Belva F, Painter RC, Schiettecatte J, Bonduelle M, Roelants M, Roseboom TJ, et al. Gender-specific alterations in salivary cortisol levels in pubertal intracytoplasmic sperm injection offspring. Horm Res Paediatr. 2013;80:350–5.

10. Belva F, Painter R, Bonduelle M, Roelants M, Devroey P, De Schepper J. Are ICSI adolescents at risk for increased adiposity? Hum Reprod. 2012;27:257–64.

11. Canalis E, Reardon GE, Caldarella AM. A more specific, liquid-chromatographic method for free cortisol in urine. Clin Chem. 1982;28:2418–20.

12. Chen FR, Raine A, Rudo-Hutt AS, Glenn AL, Soyfer L, Granger DA. Harsh discipline and behavior problems: The moderating effects of cortisol and alpha-amylase. Biol Psychol. 2014;104:19–27.

13. Liu J, Richmond T, Raine A, Cheney R, Brodkin E, Gur RC, et al. The Healthy Brains and Behavior Study: objectives, design, recruitment, and population coverage. Int J Methods Psychiatr Res. 2013;22:204–16.

14. Cicchetti D, Rogosch FA. The impact of child maltreatment and psychopathology on neuroendocrine functioning. Dev Psychopathol. 2001;13:783–804.

15. Cieslak TJ, Frost G, Klentrou P. Effects of physical activity, body fat, and salivary cortisol on mucosal immunity in children. J Appl Physiol (1985). 2003;95:2315–20.

16. Colomina MT, Canals J, Carbajo G, Domingo JL. Salivary cortisol in a young population: Relationship with psychopathological disorders. Res Commun Biol Psychol Psychiatry. 1997;22:1–10.

17. Covelli MM, Wood CE, Yarandi HN. Biologic measures as epidemiological indicators of risk for the development of hypertension in an African American adolescent population. J Cardiovasc Nurs. 2012;27:476–84.

18. Daughters SB, Gorka SM, Matusiewicz A, Anderson K. Gender specific effect of psychological stress and cortisol reactivity on adolescent risk taking. J Abnorm Child Psychol. 2013;41:749–58.

19. Davis M, Emory E. Sex differences in neonatal stress reactivity. Child Dev. 1995;66:14–27.

20. De Bruijn ATCE, Van Bakel HJA, Wijnen H, Pop VJM, Van Baar AL. Prenatal maternal emotional complaints are associated with cortisol responses in toddler and preschool aged girls. Dev Psychobiol. 2009;51:553–63.

21. Dietrich A, Ormel J, Buitelaar JK, Verhulst FC, Hoekstra PJ, Hartman CA. Cortisol in the morning and dimensions of anxiety, depression, and aggression in children from a general population and clinic-referred cohort: An integrated analysis. The TRAILS study. Psychoneuroendocrino. 2013;38:1281–98.

22. Huisman M, Oldehinkel AJ, De Winter A, Minderaa RB, De Bildt A, Huizink AC, et al. Cohort profile: The Dutch “TRacking Adolescents” individual lives’ survey'; TRAILS. Int J Epidemiol. 2008;37:1227–35.

23. Elmlinger MW, Kühnel W, Ranke MB. Reference ranges for serum concentrations of lutropin (LH), follitropin (FSH), estradiol (E2), prolactin, progesterone, sex hormone-binding globulin (SHBG), dehydroepiandrosterone sulfate (DHEAS), cortisol and ferritin in neonates, children and young adul. Clin Chem Lab Med. 2002;40:1151–60.

24. Elmlinger MW, Kühnel W, Lambrecht HG, Ranke MB. Reference intervals from birth to adulthood for serum thyroxine (T4), triiodothyronine (T3), free T3, free T4, thyroxine binding globulin (TBG) and thyrotropin (TSH). Clin Chem Lab Med. 2001;39:973–9.

25. Forest MG. Age-related response of plasma testosterone, delta 4-androstenedione, and cortisol to adrenocorticotropin in infants, children, and adults. J Clin Endocrinol Metab. 1978;47:931–7.

26. Fransson E, Folkesson L, Bergström M, Östberg V, Lindfors P. Exploring salivary cortisol and recurrent pain in mid-adolescents living in two homes. BMC Psychol. 2014;2:1–7.

27. Garagorri JM, Rodríguez G, Lario-Elboj ÁJ, Olivares JL, Lario-Muñoz Á, Orden I. Reference levels for 17-hydroxyprogesterone, 11-desoxycortisol, cortisol, testosterone, dehydroepiandrosterone sulfate and androstenedione in infants from birth to six months of age. Eur J Pediatr. 2008;167:647–53.

28. Georgopoulos NA, Rottstein L, Tsekouras A, Theodoropoulou A, Koukkou E, Mylonas P, et al. Abolished circadian rhythm of salivary cortisol in elite artistic gymnasts. Steroids. 2011;76:353–7.

29. Ghaziuddin N, Welch K, Greden J. Central serotonergic effects of m-chlorophenylpiperazine (mCPP) among normal control adolescents. Neuropsychopharmacol. 2003;28:133–9.

30. Gunnar MR, Kryzer E, Van Ryzin MJ, Phillips DA. The Rise in cortisol in family day care: Associations with aspects of care quality, child behavior, and child sex. Child Dev. 2010;81: 851–69.

31. Hackney AC, McMurray RG, Judelson DA, Harrell JS. Relationship between caloric intake, body composition, and physical activity to leptin, thyroid hormones, and cortisol in adolescents. Jpn J Physiol. 2003;53:475–9.

32. Honour JW, Jones R, Leary S, Golding J, Ong KK, Dunger DB. Relationships of urinary adrenal steroids at age 8 years with birth weight, postnatal growth, blood pressure, and glucose metabolism. J Clin Endocrinol Metab. 2007;92:4340–5.

33. Huybrechts I, De Vriendt T, Breidenassel C, Rogiers J, Vanaelst B, Cuenca-García M Moreno LA, et al; HELENA Study Group. Mechanisms of stress, energy homeostasis and insulin resistance in European adolescents - the HELENA study. Nutr Metab Cardiovasc Dis. 2014;24:1082–9.

34. Beghin L, Huybrechts I, Vicente-Rodriguez G, De Henauw S, Gottrand F, Gonzales-Gross M, et al. Mains characteristics and participation rate of European adolescents included in the HELENA study. Arch Public Heal. 2012;70:14.

35. Ilias I, Ghizzoni L, Mastorakos G. Orderliness of cortisol, growth hormone, and leptin secretion in short-normal pre-pubertal boys and girls. Med Sci Monit. 2009;15:242–7.

36. Jones A, Godfrey KM, Wood P, Osmond C, Goulden P, Phillips DIW. Fetal growth and the adrenocortical response to psychological stress. J Clin Endocrinol Metab. 2006;91:1868–71.

37. Lashansky G, Saenger P, Fishman K, Gautier T, Mayes D, Berg G, et al. Normative data for adrenal steroidogenesis in a healthy pediatric population: Age- and sex-related changes after adrenocorticotropin stimulation. J Clin Endocrinol Metab. 1991;73:674–86.

38. Lundberg U, De Chateau P, Winberg J, Frankenhaeuser M. Catecholamine and cortisol excretion patterns in three-year-old children and their parents. J Human Stress. 1981;7:3–11.

39. Lundberg U. Sex differences in behaviour pattern and catecholamine and cortisol excretion in 3-6 year old day-care children. Biol Psychol. 1983;16:109–17.

40. Martikainen S, Pesonen A-K, Lahti J, Heinonen K, Feldt K, Pyhälä R, et al. Higher levels of physical activity are associated with lower hypothalamic-pituitary-adrenocortical axis reactivity to psychosocial stress in children. J Clin Endocrinol Metab. 2013;98:E619–27.

41. Michels N, Sioen I, Huybrechts I, Bammann K, Vanaelst B, De Vriendt T, et al. Negative life events, emotions and psychological difficulties as determinants of salivary cortisol in Belgian primary school children. Psychoneuroendocrino. 2012;37:1506–15.

42. Ahrens W, Bammann K, Siani A, Buchecker K, De Henauw S, Iacoviello L, et al; IDEFICS Consortium. The IDEFICS cohort: design, characteristics and participation in the baseline survey. Int J Obes. 2011;35:S3–15.

43. Mills RS, Imm GP, Walling BR, Weiler HA. Cortisol reactivity and regulation associated with shame responding in early childhood. Dev Psychol. 2008;44:1369–80.

44. Minkley N, Kirchner WH. Influence of test tasks with different cognitive demands on salivary cortisol concentrations in school students. Int J Psychophysiol. 2012;86:245–50.

45. Mrug S, Tyson A, Turan B, Granger DA. Sleep problems predict cortisol reactivity to stress in urban adolescents. Physiol Behav. 2016;155:95–101.

46. Nakamura J, Yakata M. Age- and sex-related differences in urinary cortisol level. Clin Chim Acta. 1984;137:77–80.

47. Ong KK, Potau N, Petry CJ, Jones R, Ness AR, Honour JW, et al; Avon Longitudinal Study of Parents and Children Study Team. Opposing influences of prenatal and postnatal weight gain on adrenarche in normal boys and girls. J Clin Endocrinol Metab. 2004;89:2647–51.

48. Osika W, Friberg P, Wahrborg P. A new short self-rating questionnaire to assess stress in children. Int J Behav Med. 2007;14:108–17.

49. Pérez-Edgar K, Schmidt LA, Henderson HA, Schulkin J, Fox NA. Salivary cortisol levels and infant temperament shape developmental trajectories in boys at risk for behavioral maladjustment. Psychoneuroendocrino. 2008;33:916–25.

50. Kagan J, Snidman N. Infant Predictors of Inhibited and Uninhibited Profiles. Psychol Sci. 1991;2:40–4.

51. Portnoy J, Raine A, Glenn AL, Chen FR, Choy O, Granger DA. Digit ratio (2D:4D) moderates the relationship between cortisol reactivity and self-reported externalizing behavior in young adolescent males. Biol Psychol. 2015;112:94–106.

52. Reynolds RM, Hii HL, Pennell CE, McKeague IW, Kloet ER de, Lye S, et al. Analysis of baseline hypothalamic-pituitary-adrenal activity in late adolescence reveals gender specific sensitivity of the stress axis. Psychoneuroendocrino. 2013;38:1271–80.

53. Ross JL, Schulte HM, Gallucci WT, Cutler GB, Loriaux DL, Chrousos GP. Ovine corticotropin-releasing hormone stimulation test in normal children. J Clin Endocrinol Metab. 1986;62:390–2.

54. Soriano-Rodriguez P, Osiniri I, Grau-cabrera P, Riera-perez E, Prats-puig A, Carbonell-Alferez M, et al. Physiological Concentrations of Serum Cortisol Are Related to Vascular Risk Markers in Prepubertal Children. Pediatr Res. 2010;68:452–5.

55. Stroud LR, Papandonatos GD, Williamson DE, Dahl RE. Sex differences in cortisol response to corticotropin releasing hormone challenge over puberty: Pittsburgh Pediatric Neurobehavioral Studies. Psychoneuroendocrino. 2011;36:1226–38.

56. Stupnicki R, Obmiński Z, Klusiewicz A, Viru A. Pre-exercise serum cortisol concentration and responses to laboratory exercise. Eur J Appl Physiol Occup Physiol. 1995;71:439–43.

57. Susman EJ, Dorn LD, Chrousos GP. Negative affect and hormone levels in young adolescents: Concurrent and predictive perspectives. J Youth Adolesc. 1991;20:167–90.

58. Nottelmann ED, Susman EJ, Inoff-Germain G, Cutler GB, Loriaux DL, Chrousos GP. Developmental processes in early adolescence: Relationships between adolescent adjustment problems and chronologic age , pubertal stage , and puberty-related serum hormone levels. J Pediatr. 1987;110:473–80.

59. Syme C, Abrahamowicz M, Leonard GT, Perron M, Pitiot A, Qiu X, et al. Intra-abdominal adiposity and individual components of the metabolic syndrome in adolescence: sex differences and underlying mechanisms. Arch Pediatr Adolesc Med. 2008;162:453–61.

60. Pausova Z, Paus T, Abrahamowicz M, Almerigi J, Arbour N, Bernard M, et al. Genes, maternal smoking, and the offspring brain and body during adolescence: Design of the Saguenay Youth Study. Hum Brain Mapp. 2007;28:502–18.

61. Tennes K, Carter D. Plasma Cortisol Levels and Behavioral States in Early Infancy The purpose of this study was to examine the relationship between plasma cortisol levels and behavioral states in the infant at birth and during the first three months of life . Convincing evid. 1973;35:121–8.

62. Tornhage C. Reference values for morning salivary cortisol concentrations in healthy school-aged children. J Pediatr Endocrinol Metab. 2002;15:197–204.

63. Tout K, de Haan M, Campbell EK, Gunnar MR. Social Behavior Correlates of Cortisol Activity in Child Care: Gender Differences and Time-Of-Day Effects. Child Dev. 1998;69:1247–62.

64. Tsvetkova V. Adrenocortical function after stimulation with synthetic ACTH. Curr Med Res Opin. 1977;4:635–9.

65. Turan B, Tackett JL, Lechtreck MT, Browning WR. Coordination of the cortisol and testosterone responses: A dual axis approach to understanding the response to social status threats. Psychoneuroendocrino. 2015;62:59–68.

66. Tzortzi C, Proff P, Redlich M, Aframian DJ, Palmon A, Golan I, et al. Cortisol daily rhythm in saliva of healthy school children. Int Dent J. 2009;59:12–8.

67. Vaindirlis I, Peppa-Patrikiou M, Dracopoulou M, Manoli I, Voutetakis A, Dacou-Voutetakis C. “White coat hypertension” in adolescents: increased values of urinary cortisol and endothelin. J Pediatr 2000. 136:359–64.

68. West P, Sweeting H, Young R, Kelly S. The relative importance of family socioeconomic status and school-based peer hierarchies for morning cortisol in youth: An exporatory study. Soc Sci Med. 2010;70:1246–53.

69. Wudy SA, Hartmann MF, Remer T. Sexual dimorphism in cortisol secretion starts after age 10 in healthy children: urinary cortisol metabolite excretion rates during growth. Am J Physiol Endocrinol Metab. 2007;293:E970–6.

70. Kroke A, Manz F, Kersting M, Remer T, Sichert-Hellert W, Alexy U, et al. The DONALD Study History, current status and future perspectives. Eur J Nutr. 2004;43:45–54.

71. Remer T, Boye KR, Hartmann MF, Wudy SA. Urinary Markers of Adrenarche : Reference Values in Healthy Subjects, Aged 3 – 18 Years. J Clin Endocrinol Metab. 2005;90:2015–21.

72. Yu YZ, Shi JX. Relationship between levels of testosterone and cortisol in saliva and aggressive behaviors of adolescents. Biomed Environ Sci. 2009;22:44–9.
