# Supplementary material for: Gender-specific differences in hypothalamus–pituitary–adrenal axis activity during childhood: a systematic review and meta-analysis
Source: Biol Sex Differ. 2017 Jan 19;8:3. doi: 10.1186/s13293-016-0123-5 (PMC5244584; doi:10.1186/s13293-016-0123-5)
Supplement: Additional file 4: — Comparison fixed-effect analysis vs. random-effects analysis. (DOCX 202 kb) [file 13293_2016_123_MOESM4_ESM.docx]

**Forest plots of gender differences per subgroup: fixed-effect analysis (1) vs. random-effects analysis (2).**

**A.** Salivary cortisol (nmol/L) <8 yr of age **B.** Salivary cortisol (nmol/L) 8–18 yr of age **C.** Serum cortisol (nmol/L) <8 yr of age **D.** Serum cortisol (nmol/L) 8–18 yr of age **E.** 24h-urine cortisol (µg/24h) <8 yr of age **F.** 24h-urine cortisol (µg/24h) 8–18 yr of age.

**A1.
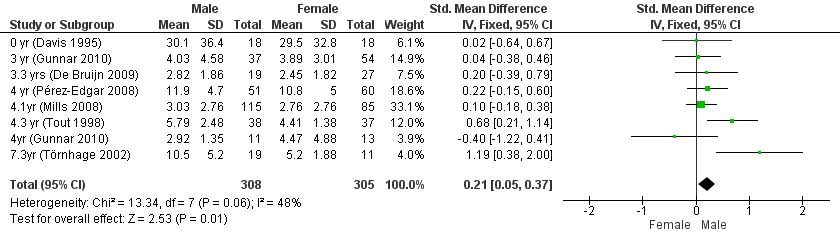
**

**A2.**

**
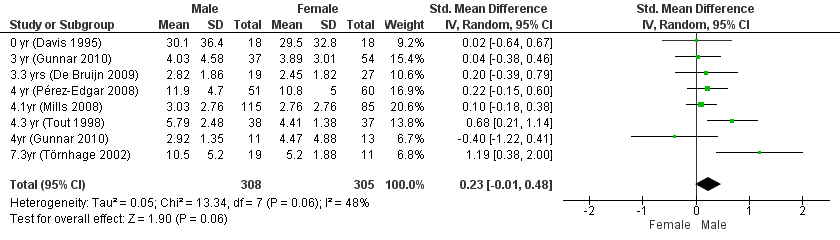
**

**B1.
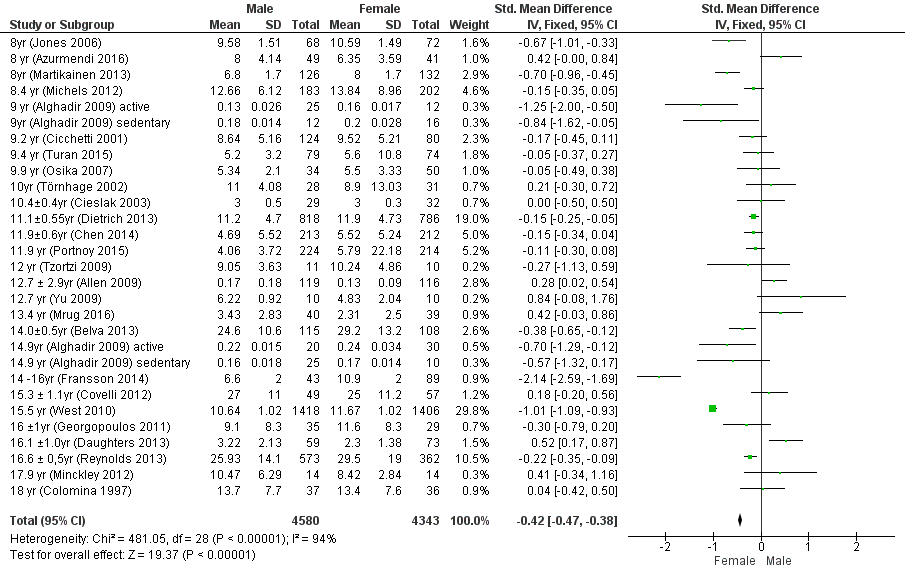
**

**B2.**

**
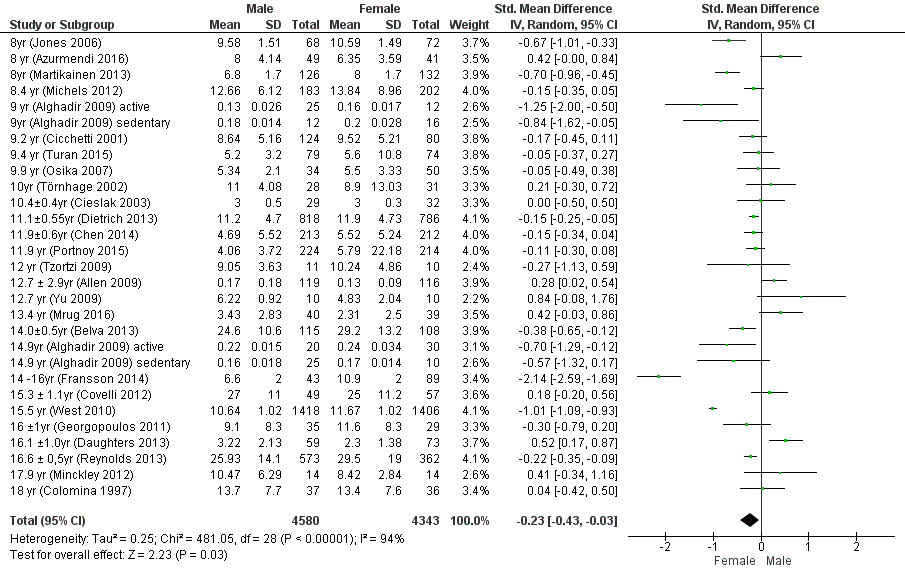
**

**C1.**

**
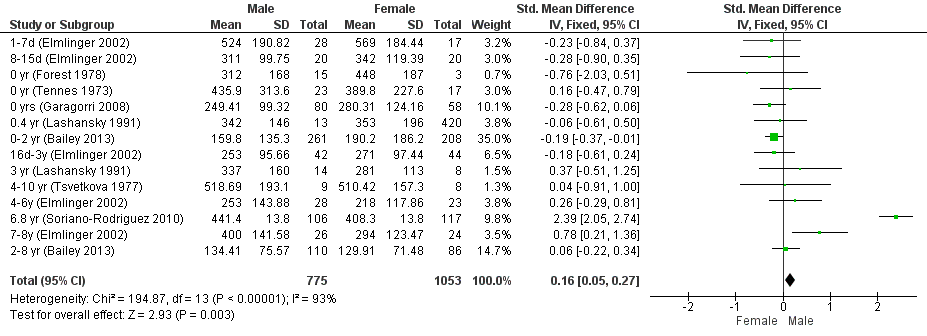
**

**C2.
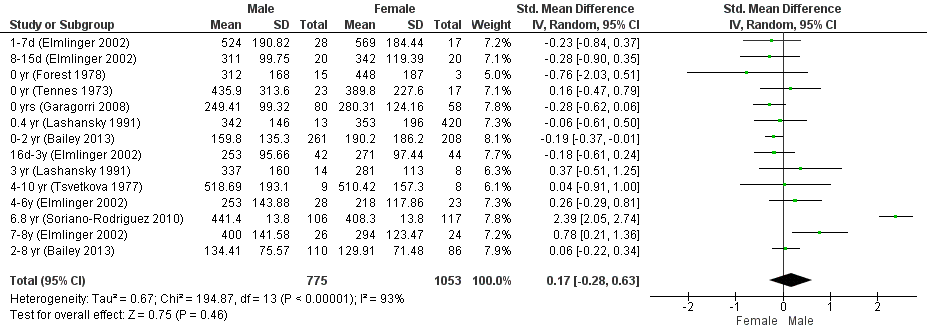
**

**D1.**

**
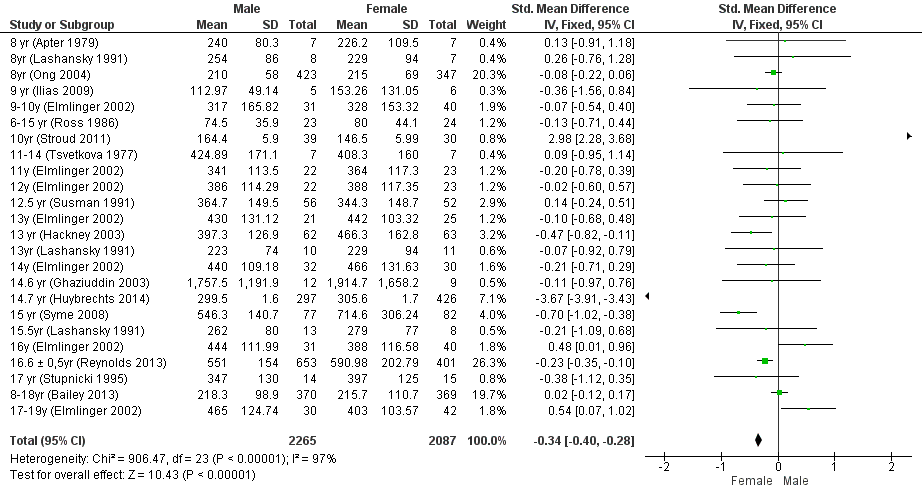
**

**D2.**

**
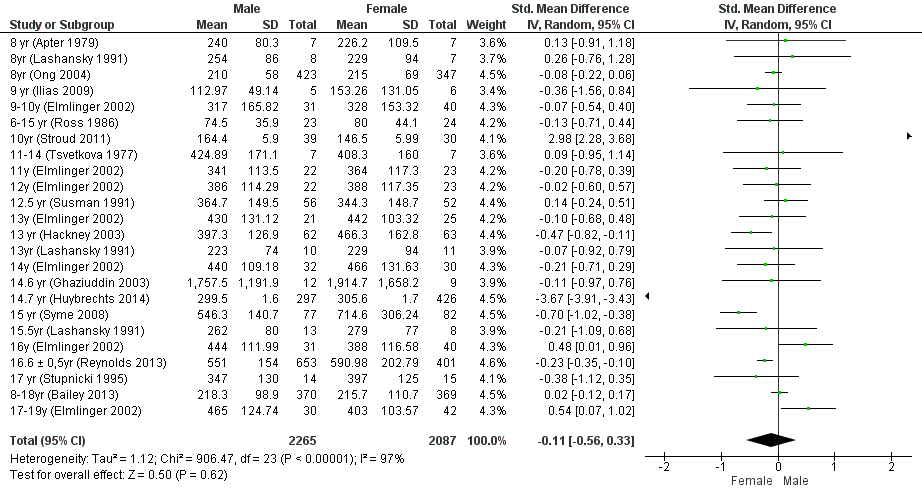
**

**E1.
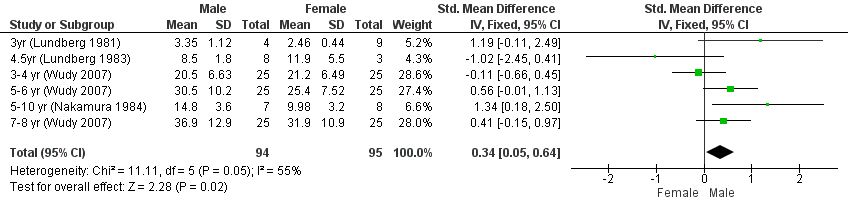
**

**E2.**

**
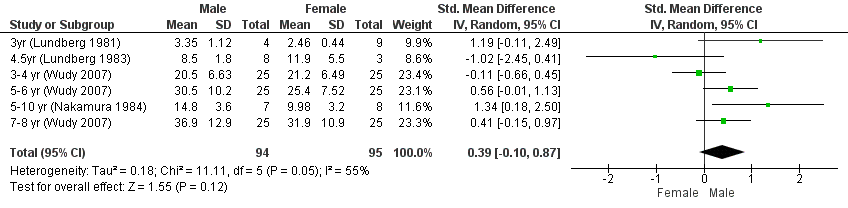
**

**F1.
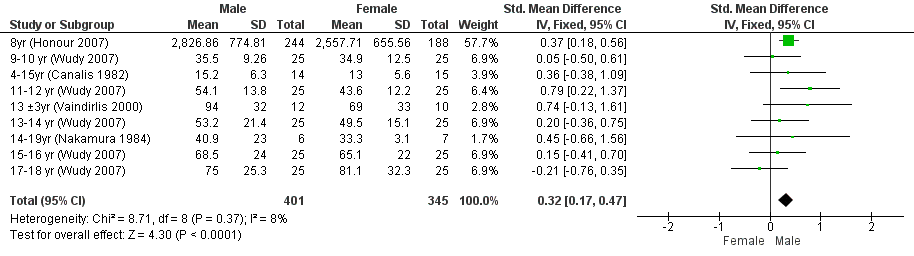
**

**F2.**

**
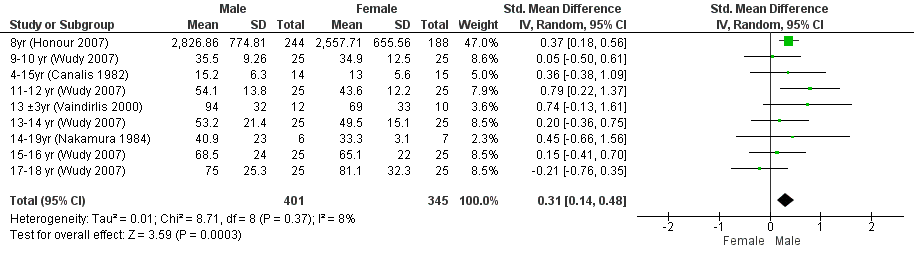
**
