# Supplementary material for: Gender-specific differences in hypothalamus–pituitary–adrenal axis activity during childhood: a systematic review and meta-analysis
Source: Biol Sex Differ. 2017 Jan 19;8:3. doi: 10.1186/s13293-016-0123-5 (PMC5244584; doi:10.1186/s13293-016-0123-5)

**Funnel plots A.** Saliva cortisol <8 yr **B.** Saliva cortisol between 8–18 yr **C.** Serum cortisol <8 yr **D.** Serum cortisol between 8–18 yr **E.** 24h-urine cortisol <8 yr **F.** 24h-urine cortisol between 8–18 yr

**A.**


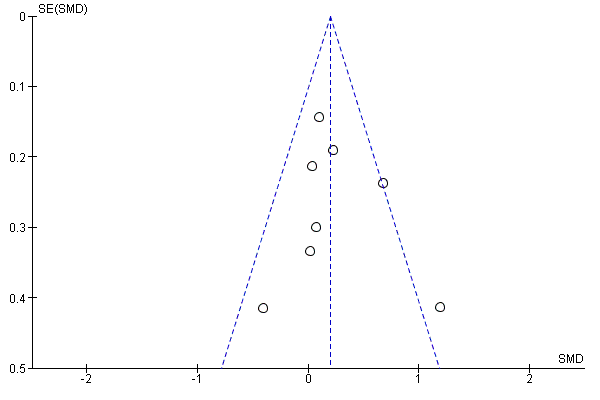


**B**.


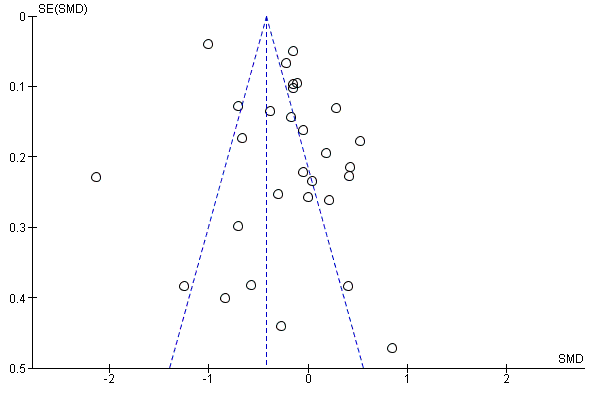


**C.**


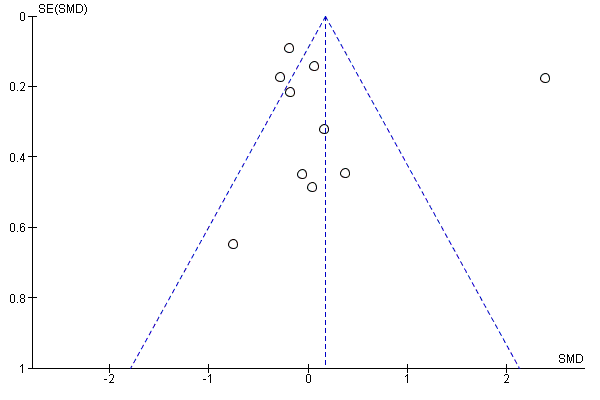


**D.**

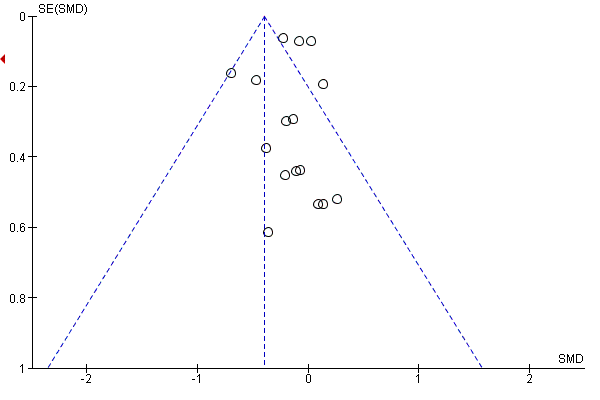


**E.**


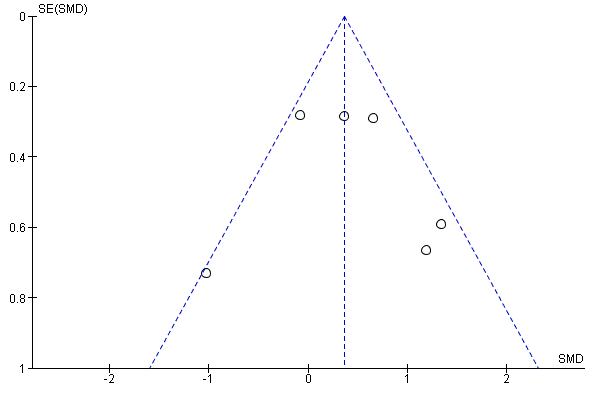


**F.**


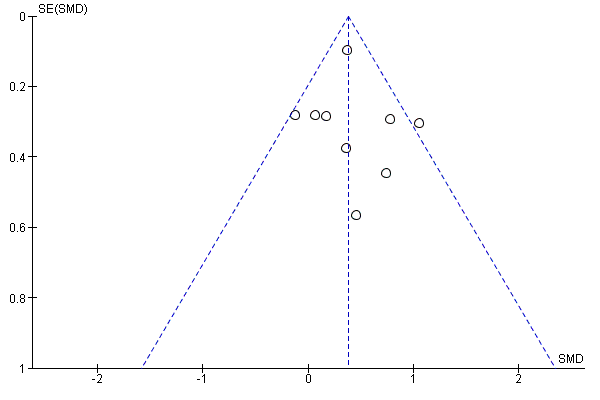

Supplement: Additional file 5: — Funnel plots. (DOCX 65 kb) [file 13293_2016_123_MOESM5_ESM.docx]
