# Supplementary material for: Gender-specific differences in hypothalamus–pituitary–adrenal axis activity during childhood: a systematic review and meta-analysis
Source: Biol Sex Differ. 2017 Jan 19;8:3. doi: 10.1186/s13293-016-0123-5 (PMC5244584; doi:10.1186/s13293-016-0123-5)
Supplement: Additional file 6: — Overview of age ranges of studies included in meta-analysis. (DOCX 20 kb) [file 13293_2016_123_MOESM6_ESM.docx]

| **Study** | **Mean age ± SD*** |  | **Study** | **Mean age ± SD*** |
| --- | --- | --- | --- | --- |
| Davis 1995 | 2 days |  | Mrug 2016 | 13.36 ± 0.95 yr |
| Forest 1978 | 115.3 ± 120.1 days |  | Vaindirlis 2000 | 13 yr ± 3.5 yr |
| Tennes 1973 | 3 days |  | Hackney 2003 | 13.4 ± 0.9 yr |
| Garagorri 2008 | 3 days |  | Belva 2013 | girls 14.0 ± 0.5 yr, boys 14.0 ± 0.4 yr |
| De Bruijn 2009 | 38.61 ± 9.4 months |  | Ghaziuddin 2003 | 14.6 ± 1.5 yr |
| Lundberg 1981 | 3 yr |  | Huybrechts 2014 | 14.7 ± 1.2 yr |
| Gunnar 2010 | 3.81 ± 0.23 yr |  | Fransson 2014 | ranges 14 - 16 yr |
| Mills 2008 | 4.14 ± 0.24 yr |  | Nakamura 1984 | 5 – 10 yr and 14 - 19 yr |
| Tout 1998 | mean 4.3 yr |  | Covelli 2012 | 15.3 ± 1.1 yr |
| Lundberg 1983 | boys mean 52.3 months, girls 54.9 months |  | West 2010 | 15.4 ± 0.4 yr |
| Soriano-Rodriguez 2010 | 6.8 ± 0.19 yr |  | Syme 2008 | boys 14.4 ± 1.7 yr, girls 14.4 ± 1.9 yr |
| Michels 2012 | boys 8.44 ± 1.18 yr, girls 8.39 ± 1.20 yr |  | Daughters 2013 | 16.1 yr ± 1.0 yr |
| Apter 1979 | range 7.5 – 8.5 yr |  | Reynolds 2013 | 16.6 yr ± 0.5 yr |
| Azurmendi 2016 | 8 yr |  | Georgopoulos 2011 | boys 15.3 ± 2.0 yr, girls 16.0 ± 1.4 yr |
| Honour 2007 | range 8.2 – 8.4 yr |  | Minckley 2012 | 17.86 ± (S.E.M.) 0.096 yr |
| Jones 2006 | range 7- 9 yr |  | Stupnicki 1995 | boys 17.3 ± 0.8 yr, girls 16.4 ± 0.6 yr |
| Martikainen 2013 | boys 8.2 ± 0.3 yr, girls 8.1 ± 0.3 yr |  | Colomina 1997 | range 17.5 – 18.5 yr |
| Ong 2004 | 8.2 ± 0.1 yr |  | Elmlinger 2002 | ranges 16 days – 3 yr, 11 yr |
| Cicchetti 2001 | 9.24 ± 2.33 yr |  | Tsvetkova 1977 | ranges 4-10 yr and 11-14 yr |
| Turan 2015  Osika 2007  Ilias 2009  Cieslak 2003 | 9.38 ± 0.62 yr  9.9 ± 0.6 yr  boys 9.5 ± 1.9 yr, girls 9.1 ± 1.3 yr  10.4 ± 0.4 yr |  | Lashansky 1991 | Boys 0.42 ± 0.24, 3.2 ± 1.6, 7.4 ± 1.8, 13.1 ± 1.2, 15.2 ± 1.4 yr  Girls 0.42 ± 0.2,2.5 ± 1.5, 9.3 ± 2.2, 12.5 ± 0.9, 15.9 ± 0.7 yr |
| Stroud 2011 | 10.5 ± 1.7 yr |  | Bailey 2013 | 0.41 ± 0.37, 5.29 ± 1.74, 13.48 ± 3.03 yr |
| Dietrich 2013  Chen 2014 | 11.1 yr ± 0.55 yr  11.87 ± 0.60 yr |  | Wudy 2007 | ranges 3-4, 5-6, 7-8, 9-10, 11-12, 13-14, 15-16, 17-18 yr |
| Portnoy 2015  Susman 1991 | 11.92 ± 0.59 yr  mean boys 12.72 yr, girls 11.99 yr |  | Alghadir 2009 | boys 9.3 ± 1.5 and 14.9 ± 3.7 yr  girls 8.96 ± 1.8 and 14.82 ± 4.6 yr |
| Allen 2009  Yu 2009 | 12.7 yr ± 2.9 yr  12.6 ± 1.8 yr |  | Törnhage 2002 | median girls 7.4 and 10.3 yr,  boys 7.1 and 10.2 yr |
| Ross 1986  Canalis 1982 | range 6-15 yr  range 4-15 yr |  | Tzortzi 2009 | ranges boys 10 yr and 3 months – 13 yr and 7 months, girls 10 yr and 3 months – 13 yr and 3 months |

* unless otherwise indicated
